# Supplementary material for: Clinical efficacy and safety of drug interventions for primary and secondary prevention of osteoporotic fractures in postmenopausal women: Network meta-analysis followed by factor and cluster analysis
Source: PLoS One. 2020 Jun 3;15(6):e0234123. doi: 10.1371/journal.pone.0234123 (PMC7269244; doi:10.1371/journal.pone.0234123)
Supplement: S2 Appendix — (PDF) [file pone.0234123.s002.pdf]

The full search strategy:

| | |
|---|---|
| #1 | "Osteoporosis, Postmenopausal"[Mesh] OR "Postmenopausal Osteoporosis" OR "Postmenopausal Osteoporoses" OR "Postmenopausal Osteopenia" OR "Postmenopausal Bone Loss*" OR "Post-Menopausal Osteoporos*" OR "Post-Menopausal Osteopenia" OR "Post-Menopausal Bone Loss" OR "Perimenopausal Osteoporos*" OR "Perimenopausal Osteopenia" OR "Perimenopausal Bone Loss*" |
| #2 | ("Postmenopausal Women" OR "Women") AND ("Osteoporosis"[Mesh] OR "Fractures, Bone"[Mesh] OR "Osteoporosis" OR "Osteoporoses" OR "fracture*" OR "Bone Loss*" OR "Osteopenia" OR "Low Bone Mass" OR "Low Bone Mineral Density" OR "Low Bone Density" OR "Low Bone Mineral Densities" OR "Low Bone Densities" OR "Low Bone Mineral Content" OR "Low Bone Mineral Contents") |
| #3 | "zoledronic acid"[Supplementary Concept] OR "zoledronic acid" OR "zoledronate" OR "2-(imidazol-1-yl)-1-hydroxyethylidene-1,1-bisphosphonic acid" OR "zoledronic acid anhydrous" OR "Zometa" OR "CGP 42446A" OR "CGP-42446" OR "CGP-42'446" OR "CGP 42'446" OR "1 hydroxy 2 (1 imidazolyl) 1, 1 ethanebisphosphonic acid" OR "1 hydroxy 2 (1h imidazol 1 yl) ethylidenebisphosphonic acid" OR "1 hydroxy 2 (imidazol 1 yl) ethylidenebisphosphonic acid" OR "aclasta" OR "cgp 42446" OR "cgp 42446a" OR "cgp42446" OR "cgp42446a" OR "orazol" OR "reclast" OR "zol 446" OR "zol446" OR "zoledronate" OR "zoledronate disodium" OR "zoledronate trisodium" OR "zoledronic acid disodium salt hydrate" OR "zoledronic acid hydrate" OR "zoledronic acid monohydrate" OR "zomera" OR "zometa" |
| #4 | "Alendronate"[Mesh] OR "Alendronate" OR "Aminohydroxybutane Bisphosphonate" OR "Bisphosphonate, Aminohydroxybutane" OR "4-Amino-1-Hydroxybutylidene 1,1-Biphosphonate" OR "4 Amino 1 Hydroxybutylidene 1,1 Biphosphonate" OR "Alendronate Sodium" OR "Sodium, Alendronate" OR "Alendronate Monosodium Salt, Trihydrate" OR "Fosamax" OR "MK-217" OR "MK 217" OR "MK217" OR "4 amino 1 hydroxy 1, 1 butanebisphosphonic acid" OR "4 amino 1 hydroxy 1, 1 butanediphosphonic acid" OR "4 amino 1 hydroxybutane 1, 1 diphosphonate" OR "4 amino 1 hydroxybutane 1, 1 diphosphonic acid" OR "4 amino 1 hydroxybutylidene 1, 1 bisphosphonate" OR "4 amino 1 hydroxybutylidene 1, 1 bisphosphonic acid" OR "4 amino 1 hydroxybutylidene 1, 1 diphosphonate" OR "4 amino 1 hydroxybutylidene 1, 1 diphosphonic acid" OR "alenato" OR "alend" OR "alendronate" OR "alendronate sodium" OR "alendronate sodium trihydrate" OR "alendros" OR "alovell" OR "arendal" OR "bifemelan" OR "bifosa" OR "binosto" OR "bonapex" OR "defixal" OR "dronal" OR "endronax" OR "eucalen" OR "fixopan" OR "fosalan" OR "fosamax" OR "fosmin" OR "fosval" OR "marvil" OR "maxibone" OR "maxibone 70" OR "mk 0217" OR "mk 217" OR "mk0217" OR "mk217" OR "neobon" OR "oncalst" OR "onclast" OR "osdron" OR "osdronat" OR "oseotenk" OR "osficar" OR "oslene" OR "osteofar" OR "osteofos" OR "osteopor" OR "osteosan" OR |

| | |
|---|---|
| | "osteovan" OR "osticalcin" OR "porosal" OR "sodium alendronate" OR "teiroc" OR "tibolene" OR "voroste" |
| #5 | "Risedronic Acid"[Mesh] OR "Risedronate Sodium" OR "Risedronate" OR "Sodium, Risedronate" OR "2-(3-pyridinyl)-1-hydroxyethylidene-bisphosphonate" OR "1-Hydroxy-2-(3-pyridyl)ethylidene diphosphonate" OR "2-(3-pyridinyl)-1-hydroxyethylidenebisphosphonate" OR "Risedronic Acid, Monosodium Salt" OR "Bisphosphate Risedronate Sodium" OR "Risedronate Sodium, Bisphosphate" OR "Sodium, Bisphosphate Risedronate" OR "Risedronic Acid" OR "Acid, Risedronic" OR "Actonel" OR "Atelvia" OR "1 hydroxy 2 (3 pyridinyl) ethylidene 1, 1 bisphosphonic acid" OR "1 hydroxy 2 (3 pyridyl) 1, 1 ethanebisphosphonic acid" OR "1 hydroxy 2 (3 pyridyl) ethylidene 1, 1 bisphosphonate" OR "1 hydroxy 2 (3 pyridyl) ethylidene 1, 1 bisphosphonic acid" OR "2 (3 pyridinyl) 1 hydroxyethylidene 1, 1 bisphosphonic acid" OR "actonel" OR "actonel once a week" OR "atelvia" OR "benet" OR "ne 58095" OR "ne58095" OR "optinate" OR "ribastamin" OR "risedronate" OR "risedronate monosodium hemipentahydrate" OR "risedronate sodium" |
| #6 | "Ibandronic Acid" [mh] OR "Ibandronate" OR "Ibandronic Acid" OR "1-Hydroxy-3-(methylpentylamino)propylidenebisphosphonate" OR "(1-Hydroxy-3-(methylpentylamino)propylidene)bisphosphonate" OR "Ibandronate Sodium Anhydrous" OR "Boniva" OR "Bonviva" OR "Ibandronate Sodium" OR "RPR 102289A" OR "Bondronat" OR "BM 21.0955" OR "BM 210955" OR "BM-210955" |
| #7 | "Teriparatide"[Mesh] OR "Teriparatide" OR "hPTH (1-34)" OR "Human Parathyroid Hormone (1-34)" OR "Parathar" OR "Teriparatide Acetate" OR "Forteo" OR "bovine parathyroid hormone [1-34]" OR "chs 13340" OR "chs13340" OR "forsteo" OR "forteo" OR "ly 333334" OR "ly333334" OR "movymia" OR "parathar" OR "parathormone 1 34" OR "parathormone 1-34" OR "parathormone (1-34)" OR "parathormone [1-34]" OR "parathyroid hormone 1 34" OR "parathyroid hormone 1-34" OR "parathyroid hormone [1 34]" OR "parathyroid hormone [1-34]" OR "parathyroid hormone (1-34)" OR "parathyroid hormone [1 34]" OR "parathyroid hormone [1-34] peptide" OR "pth [1-34]" OR "sun e3001" OR "sune3001" OR "teriparatide" OR "teriparatide (rDNA origin)" OR "teriparatide acetate" OR "teriparatide recombinant human" OR "terrosa" |
| #8 | "abaloparatide"[Supplementary Concept] OR "abaloparatide" OR "Parathyroid Hormone-Related Protein" OR "ba 058" OR "ba058" OR "bim 44058" OR "bim44058" OR "eladynos" OR "tymlos" |
| #9 | "AMG 785" [Supplementary Concept] OR "AMG 785" OR "AMG785" OR "AMG-785" OR "CDP 7851" OR "CDP7851" OR "CDP-7851" OR "romosozumab" OR "amg 785" OR "amg785" OR "cdp 7851" OR "cdp7851" |
| #10 | "Parathyroid Hormone" [MH] OR "Parathyroid Hormone (1-84)" OR "PTH (1-84)" OR "PTH" OR "Parathormone" OR "Parathyroid Hormone Peptide (1-84)" |
| #11 | "Denosumab" [Mesh] OR "Denosumab" OR "Xgeva" OR "AMG 162" OR "Prolia" OR "amg 162" OR "amg162" OR "amgiva" OR "prolia" OR "xgeva" |
| #12 | "Raloxifene Hydrochloride" [Mesh] OR "Raloxifene Hydrochloride" OR |

| | |
|---|---|
| | "Keoxifene Hydrochloride" OR "Raloxifene HCl" OR "LY-139481" OR "LY139481" OR "LY 139481" OR "Raloxifene" OR "Keoxifene" OR "Evista" OR "LY-156758" OR "LY156758" OR "LY 156758" OR "6 hydroxy 2 (4 hydroxyphenyl) 3 [4 [2 (1 piperidyl) ethoxy] benzoyl] benzo [b] thiophene" OR "6 hydroxy 2 (4 hydroxyphenyl) benzo [b] thien 3 yl 4 [2 (1 piperidinyl) ethoxy] phenyl ketone" OR "[6 hydroxy 2 (4 hydroxyphenyl) benzo [b] thien 3 yl] [4 [2 (1 piperidinyl) ethoxy] phenyl] methanone" OR "bonmax" OR "celvista" OR "evista" OR "keoxifene" OR "keoxifene hydrochloride" OR "loxar" OR "loxifen" OR "ly 139481" OR "ly 156758" OR "ly139481" OR "ly156758" OR "optruma" OR "raloxifene hydrochloride" OR "raxeto" |
| #13 | bazedoxifene[Supplementary Concept] OR "bazedoxifene" OR "TSE 424" OR "TSE424" OR "TSE-424" OR "WAY-140424" OR "bazedoxifene acetate" |
| #14 | Lasofoxifene[Supplementary Concept] OR "Lasofoxifene" OR "(-)-cis-5,6,7,8-tetrahydro-6-phenyl-5-(p-(2-(1-pyrrolidinyl)ethoxy)phenyl)-2-naphthol" OR "CP 336156" OR "CP-336,156" OR "lasofoxifene hydrochloride" OR "cis-1R-(4'-pyrrolidinoethoxyphenyl)-2S-phenyl-6-hydroxy-1,2,3,4-tetrahydronaphthalene, tartrate salt" OR "LAS estrogen receptor modulator" OR "1 [4 (2 pyrrolidinoethoxy) phenyl] 2 phenyl 6 hydroxy 1, 2, 3, 4 tetrahydronaphthalene" OR "5, 6, 7, 8 tetrahydro 6 phenyl 5 [4 [2 (1 pyrrolidinyl) ethoxy] phenyl] 2 naphthol" OR "cp 336156" OR "cp336156" OR "fablyn" OR "lasofoxifene tartrate" OR "oporia" |
| #15 | strontium ranelate[Supplementary Concept] OR "strontium ranelate" OR "protelos" OR "S12911-5" OR "S12911-0" OR "S12911-2" OR "3-(3-cyano-4-carboxymethyl-5-carboxy-2-thienyl)-3-azapentanedioic distrontium salt" OR "S 12911" OR "S12911" OR "S-12911" OR "2 [2 carboxy 4 cyano 5 [n, n bis (carboxymethyl) amino] thiophen 3 yl] acetic acid distrontium salt" OR "5 [bis (carboxymethyl) amino] 2 carboxy 4 cyanothiophen 3 ylacetic acid distrontium salt" OR "fk 481" OR "fk481" OR "osseor" OR "protelos" OR "protos" OR "ranelate strontium" OR "ranelic acid distrontium salt" OR "s 12911" OR "s 12911 2" OR "s12911" OR "s12911 2" |
| #16 | randomized controlled trial[pt] OR controlled clinical trial[pt] OR randomized[tiab] OR placebo[tiab] OR clinical trials as topic[mesh:noexp] OR randomly[tiab] OR trial[ti] |
| #17 | animals[mh] NOT humans[mh] |
| #18 | #1 OR #2 |
| #19 | #3 OR #4 OR #5 OR #6 OR #7 OR #8 OR #9 OR #10 OR #11 OR #12 OR #13 OR #14 OR #15 |
| #20 | #16 NOT #17 |
| #21 | #18 AND #19 AND #20 |
