# Supplementary material for: Clinical efficacy and safety of drug interventions for primary and secondary prevention of osteoporotic fractures in postmenopausal women: Network meta-analysis followed by factor and cluster analysis
Source: PLoS One. 2020 Jun 3;15(6):e0234123. doi: 10.1371/journal.pone.0234123 (PMC7269244; doi:10.1371/journal.pone.0234123)
Supplement: S3 Appendix — (PDF) [file pone.0234123.s003.pdf]

Studies included for quantitative synthesis:

1. Saag KG, Petersen J, Brandi ML, Karaplis AC, Lorentzon M, Thomas T, et al. Romosozumab or Alendronate for Fracture Prevention in Women with Osteoporosis. N Engl J Med. 2017;377(15): 1417-1427. https://doi.org/10.1056/NEJMoa1708322
2. Cosman F, Crittenden DB, Adachi JD, Binkley N, Czerwinski E, Ferrari S, et al. Romosozumab Treatment in Postmenopausal Women with Osteoporosis. N Engl J Med. 2016;375(16): 1532-1543. https://doi.org/10.1056/NEJMoa1607948
3. Miller PD, Hattersley G, Riis BJ, Williams GC, Lau E, Russo LA, et al. Effect of Abaloparatide vs Placebo on New Vertebral Fractures in Postmenopausal Women With Osteoporosis: A Randomized Clinical Trial. JAMA. 2016;316(7): 722-733. https://doi.org/10.1001/jama.2016.11136
4. Langdahl BL, Libanati C, Crittenden DB, Bolognese MA, Brown JP, Daizadeh NS, et al. Romosozumab (sclerostin monoclonal antibody) versus teriparatide in postmenopausal women with osteoporosis transitioning from oral bisphosphonate therapy: a randomised, open-label, phase 3 trial. LANCET. 2017;390(10102): 1585-1594. https://doi.org/10.1016/S0140-6736(17)31613-6
5. Kendler DL, Marin F, Zerbini C, Russo LA, Greenspan SL, Zikan V, et al. Effects of teriparatide and risedronate on new fractures in post-menopausal women with severe osteoporosis (VERO): a multicentre, double-blind, double-dummy, randomised controlled trial. LANCET. 2018;391(10117): 230-240. https://doi.org/10.1016/S0140-6736(17)32137-2
6. Cummings SR, Ensrud K, Delmas PD, LaCroix AZ, Vukicevic S, Reid DM, et al. Lasofoxifene in postmenopausal women with osteoporosis. N Engl J Med. 2010;362(8): 686-696. https://doi.org/10.1056/NEJMoa0808692
7. Meunier PJ, Roux C, Seeman E, Ortolani S, Badurski JE, Spector TD, et al. The effects of strontium ranelate on the risk of vertebral fracture in women with postmenopausal osteoporosis. N Engl J Med. 2004;350(5): 459-468. https://doi.org/10.1056/NEJMoa022436
8. Reginster JY, Felsenberg D, Boonen S, Diez-Perez A, Rizzoli R, Brandi ML, et al. Effects of long-term strontium ranelate treatment on the risk of nonvertebral and vertebral fractures in postmenopausal osteoporosis: Results of a five-year, randomized, placebo-controlled trial. Arthritis Rheum. 2008;58(6): 1687-1695. https://doi.org/10.1002/art.23461
9. Roux C, Fechtenbaum J, Kolta S, Isaia G, Andia JB, Devogelaer JP. Strontium ranelate reduces the risk of vertebral fracture in young postmenopausal women with severe osteoporosis. ANN RHEUM DIS. 2008;67(12): 1736-1738. https://doi.org/10.1136/ard.2008.094516
10. Hadji P, Zanchetta JR, Russo L, Recknor CP, Saag KG, McKiernan FE, et al. The effect of teriparatide compared with risedronate on reduction of back pain in postmenopausal women with osteoporotic vertebral fractures. Osteoporos Int. 2012;23(8): 2141-2150. https://doi.org/10.1007/s00198-011-1856-y
11. Reginster J, Minne HW, Sorensen OH, Hooper M, Roux C, Brandi ML, et al. Randomized trial of the effects of risedronate on vertebral fractures in women with

established postmenopausal osteoporosis. Vertebral Efficacy with Risedronate Therapy (VERT) Study Group. Osteoporos Int. 2000;11(1): 83-91.
12. Fogelman I, Ribot C, Smith R, Ethgen D, Sod E, Reginster JY. Risedronate reverses bone loss in postmenopausal women with low bone mass: results from a multinational, double-blind, placebo-controlled trial. BMD-MN Study Group. J Clin Endocrinol Metab. 2000;85(5): 1895-1900. https://doi.org/10.1210/jcem.85.5.6603
13. Cosman F, Eriksen EF, Recknor C, Miller PD, Guanabens N, Kasperk C, et al. Effects of intravenous zoledronic acid plus subcutaneous teriparatide [rhPTH(1-34)] in postmenopausal osteoporosis. J BONE MINER RES. 2011;26(3): 503-511. https://doi.org/10.1002/jbmr.238
14. Neer RM, Arnaud CD, Zanchetta JR, Prince R, Gaich GA, Reginster JY, et al. Effect of parathyroid hormone (1-34) on fractures and bone mineral density in postmenopausal women with osteoporosis. N Engl J Med. 2001;344(19): 1434-1441. https://doi.org/10.1056/NEJM200105103441904
15. McClung MR, San MJ, Miller PD, Civitelli R, Bandeira F, Omizo M, et al. Opposite bone remodeling effects of teriparatide and alendronate in increasing bone mass. Arch Intern Med. 2005;165(15): 1762-1768. https://doi.org/10.1001/archinte.165.15.1762
16. Panico A, Lupoli GA, Marciello F, Lupoli R, Cacciapuoti M, Martinelli A, et al. Teriparatide vs. alendronate as a treatment for osteoporosis: changes in biochemical markers of bone turnover, BMD and quality of life. Med Sci Monit. 2011;17(8): R442-R448. https://doi.org/10.12659/msm.881905
17. Liberman UA, Weiss SR, Broll J, Minne HW, Quan H, Bell NH, et al. Effect of oral alendronate on bone mineral density and the incidence of fractures in postmenopausal osteoporosis. The Alendronate Phase III Osteoporosis Treatment Study Group. N Engl J Med. 1995;333(22): 1437-1443. https://doi.org/10.1056/NEJM199511303332201
18. Black DM, Schwartz AV, Ensrud KE, Cauley JA, Levis S, Quandt SA, et al. Effects of continuing or stopping alendronate after 5 years of treatment: the Fracture Intervention Trial Long-term Extension (FLEX): a randomized trial. JAMA. 2006;296(24): 2927-2938. https://doi.org/10.1001/jama.296.24.2927
19. Black DM, Cummings SR, Karpf DB, Cauley JA, Thompson DE, Nevitt MC, et al. Randomised trial of effect of alendronate on risk of fracture in women with existing vertebral fractures. Fracture Intervention Trial Research Group. LANCET. 1996;348(9041): 1535-1541. https://doi.org/10.1016/s0140-6736(96)07088-2
20. Cummings SR, Black DM, Thompson DE, Applegate WB, Barrett-Connor E, Musliner TA, et al. Effect of alendronate on risk of fracture in women with low bone density but without vertebral fractures: results from the Fracture Intervention Trial. JAMA. 1998;280(24): 2077-2082. https://doi.org/10.1001/jama.280.24.2077
21. Harris ST, Watts NB, Genant HK, McKeever CD, Hangartner T, Keller M, et al. Effects of risedronate treatment on vertebral and nonvertebral fractures in women with postmenopausal osteoporosis: a randomized controlled trial. Vertebral Efficacy With Risedronate Therapy (VERT) Study Group. JAMA. 1999;282(14):

1344-1352.
22. McClung MR, Geusens P, Miller PD, Zippel H, Bensen WG, Roux C, et al. Effect of risedronate on the risk of hip fracture in elderly women. Hip Intervention Program Study Group. N Engl J Med. 2001;344(5): 333-340. https://doi.org/10.1056/NEJM200102013440503
23. Black DM, Delmas PD, Eastell R, Reid IR, Boonen S, Cauley JA, et al. Once-yearly zoledronic acid for treatment of postmenopausal osteoporosis. N Engl J Med. 2007;356(18): 1809-1822. https://doi.org/10.1056/NEJMoa067312
24. Reid IR, Horne AM, Mihov B, Stewart A, Garratt E, Wong S, et al. Fracture Prevention with Zoledronate in Older Women with Osteopenia. N Engl J Med. 2018;379(25): 2407-2416. https://doi.org/10.1056/NEJMoa1808082
25. Bone HG, Bolognese MA, Yuen CK, Kendler DL, Wang H, Liu Y, et al. Effects of denosumab on bone mineral density and bone turnover in postmenopausal women. J Clin Endocrinol Metab. 2008;93(6): 2149-2157. https://doi.org/10.1210/jc.2007-2814
26. Cummings SR, San MJ, McClung MR, Siris ES, Eastell R, Reid IR, et al. Denosumab for prevention of fractures in postmenopausal women with osteoporosis. N Engl J Med. 2009;361(8): 756-765. https://doi.org/10.1056/NEJMoa0809493
27. McClung MR, Lewiecki EM, Cohen SB, Bolognese MA, Woodson GC, Moffett AH, et al. Denosumab in postmenopausal women with low bone mineral density. N Engl J Med. 2006;354(8): 821-831. https://doi.org/10.1056/NEJMoa044459
28. Miller PD, Pannacciulli N, Brown JP, Czerwinski E, Nedergaard BS, Bolognese MA, et al. Denosumab or Zoledronic Acid in Postmenopausal Women With Osteoporosis Previously Treated With Oral Bisphosphonates. J Clin Endocrinol Metab. 2016;101(8): 3163-3170. https://doi.org/10.1210/jc.2016-1801
29. Greenspan SL, Perera S, Ferchak MA, Nace DA, Resnick NM. Efficacy and safety of single-dose zoledronic acid for osteoporosis in frail elderly women: a randomized clinical trial. JAMA INTERN MED. 2015;175(6): 913-921. https://doi.org/10.1001/jamainternmed.2015.0747
30. Recknor C, Czerwinski E, Bone HG, Bonnick SL, Binkley N, Palacios S, et al. Denosumab compared with ibandronate in postmenopausal women previously treated with bisphosphonate therapy: a randomized open-label trial. OBSTET GYNECOL. 2013;121(6): 1291-1299. https://doi.org/10.1097/AOG.0b013e318291718c
31. Miller PD, Epstein S, Sedarati F, Reginster JY. Once-monthly oral ibandronate compared with weekly oral alendronate in postmenopausal osteoporosis: results from the head-to-head MOTION study. CURR MED RES OPIN. 2008;24(1): 207-213. https://doi.org/10.1185/030079908X253889
32. McClung MR, Bolognese MA, Sedarati F, Recker RR, Miller PD. Efficacy and safety of monthly oral ibandronate in the prevention of postmenopausal bone loss. BONE. 2009;44(3): 418-422. https://doi.org/10.1016/j.bone.2008.09.011
33. Bock O, Borst H, Beller G, Armbrecht G, Degner C, Martus P, et al. Impact of oral ibandronate 150 mg once monthly on bone structure and density in post-

menopausal osteoporosis or osteopenia derived from in vivo muCT. BONE. 2012;50(1): 317-324. https://doi.org/10.1016/j.bone.2011.10.027

34. Greenspan SL, Bone HG, Ettinger MP, Hanley DA, Lindsay R, Zanchetta JR, et al. Effect of recombinant human parathyroid hormone (1-84) on vertebral fracture and bone mineral density in postmenopausal women with osteoporosis: a randomized trial. ANN INTERN MED. 2007;146(5): 326-339. https://doi.org/10.7326/0003-4819-146-5-200703060-00005
35. Fogelman I, Fordham JN, Fraser WD, Spector TD, Christiansen C, Morris SA, et al. Parathyroid hormone(1-84) treatment of postmenopausal women with low bone mass receiving hormone replacement therapy. Calcif Tissue Int. 2008;83(2): 85-92. https://doi.org/10.1007/s00223-008-9152-3
36. Ensrud KE, Stock JL, Barrett-Connor E, Grady D, Mosca L, Khaw KT, et al. Effects of raloxifene on fracture risk in postmenopausal women: the Raloxifene Use for the Heart Trial. J BONE MINER RES. 2008;23(1): 112-120. https://doi.org/10.1359/jbmr.070904
37. Ettinger B, Black DM, Mitlak BH, Knickerbocker RK, Nickelsen T, Genant HK, et al. Reduction of vertebral fracture risk in postmenopausal women with osteoporosis treated with raloxifene: results from a 3-year randomized clinical trial. Multiple Outcomes of Raloxifene Evaluation (MORE) Investigators. JAMA. 1999;282(7): 637-645.
38. Sambrook PN, Geusens P, Ribot C, Solimano JA, Ferrer-Barriendos J, Gaines K, et al. Alendronate produces greater effects than raloxifene on bone density and bone turnover in postmenopausal women with low bone density: results of EFFECT (Efficacy of FOSAMAX versus EVISTA Comparison Trial) International. J INTERN MED. 2004;255(4): 503-511. https://doi.org/10.1111/j.1365-2796.2004.01317.x
39. Recker RR, Kendler D, Recknor CP, Rooney TW, Lewiecki EM, Utian WH, et al. Comparative effects of raloxifene and alendronate on fracture outcomes in postmenopausal women with low bone mass. BONE. 2007;40(4): 843-851. https://doi.org/10.1016/j.bone.2006.11.001
40. Lufkin EG, Whitaker MD, Nickelsen T, Argueta R, Caplan RH, Knickerbocker RK, et al. Treatment of established postmenopausal osteoporosis with raloxifene: a randomized trial. J BONE MINER RES. 1998;13(11): 1747-1754. https://doi.org/10.1359/jbmr.1998.13.11.1747
41. Silverman SL, Chines AA, Kendler DL, Kung AW, Teglbjaerg CS, Felsenberg D, et al. Sustained efficacy and safety of bazedoxifene in preventing fractures in postmenopausal women with osteoporosis: results of a 5-year, randomized, placebo-controlled study. Osteoporos Int. 2012;23(1): 351-363. https://doi.org/10.1007/s00198-011-1691-1
42. Luckey M, Kagan R, Greenspan S, Bone H, Kiel RD, Simon J, et al. Once-weekly alendronate 70 mg and raloxifene 60 mg daily in the treatment of postmenopausal osteoporosis. MENOPAUSE. 2004;11(4): 405-415.
43. Greenspan SL, Schneider DL, McClung MR, Miller PD, Schnitzer TJ, Bonin R, et al. Alendronate improves bone mineral density in elderly women with osteoporosis

residing in long-term care facilities. A randomized, double-blind, placebo-controlled trial. ANN INTERN MED. 2002;136(10): 742-746. https://doi.org/10.7326/0003-4819-136-10-200205210-00009
44. Eastell R, Nickelsen T, Marin F, Barker C, Hadji P, Farrerons J, et al. Sequential treatment of severe postmenopausal osteoporosis after teriparatide: final results of the randomized, controlled European Study of Forsteo (EUROFORS). J BONE MINER RES. 2009;24(4): 726-736. https://doi.org/10.1359/jbmr.081215
45. Kendler DL, Roux C, Benhamou CL, Brown JP, Lillestol M, Siddhanti S, et al. Effects of denosumab on bone mineral density and bone turnover in postmenopausal women transitioning from alendronate therapy. J BONE MINER RES. 2010;25(1): 72-81. https://doi.org/10.1359/jbmr.090716
46. Black DM, Greenspan SL, Ensrud KE, Palermo L, McGowan JA, Lang TF, et al. The effects of parathyroid hormone and alendronate alone or in combination in postmenopausal osteoporosis. N Engl J Med. 2003;349(13): 1207-1215. https://doi.org/10.1056/NEJMoa031975
47. Tucci JR, Tonino RP, Emkey RD, Peverly CA, Kher U, Santora AN. Effect of three years of oral alendronate treatment in postmenopausal women with osteoporosis. AM J MED. 1996;101(5): 488-501. https://doi.org/10.1016/s0002-9343(96)00282-3
48. Silverman SL, Christiansen C, Genant HK, Vukicevic S, Zanchetta JR, de Villiers TJ, et al. Efficacy of bazedoxifene in reducing new vertebral fracture risk in postmenopausal women with osteoporosis: results from a 3-year, randomized, placebo-, and active-controlled clinical trial. J BONE MINER RES. 2008;23(12): 1923-1934. https://doi.org/10.1359/jbmr.080710
49. Pols HA, Felsenberg D, Hanley DA, Stepan J, Munoz-Torres M, Wilkin TJ, et al. Multinational, placebo-controlled, randomized trial of the effects of alendronate on bone density and fracture risk in postmenopausal women with low bone mass: results of the FOSIT study. Fosamax International Trial Study Group. Osteoporos Int. 1999;9(5): 461-468.
50. Meunier PJ, Slosman DO, Delmas PD, Sebert JL, Brandi ML, Albanese C, et al. Strontium ranelate: dose-dependent effects in established postmenopausal vertebral osteoporosis--a 2-year randomized placebo controlled trial. J Clin Endocrinol Metab. 2002;87(5): 2060-2066. https://doi.org/10.1210/jcem.87.5.8507
51. Bone HG, Downs RJ, Tucci JR, Harris ST, Weinstein RS, Licata AA, et al. Dose-response relationships for alendronate treatment in osteoporotic elderly women. Alendronate Elderly Osteoporosis Study Centers. J Clin Endocrinol Metab. 1997;82(1): 265-274. https://doi.org/10.1210/jcem.82.1.3682
52. McClung M, Miller P, Recknor C, Mesenbrink P, Bucci-Rechtweg C, Benhamou CL. Zoledronic acid for the prevention of bone loss in postmenopausal women with low bone mass: a randomized controlled trial. OBSTET GYNECOL. 2009;114(5): 999-1007. https://doi.org/10.1097/AOG.0b013e3181bdce0a
53. McClung MR, Siris E, Cummings S, Bolognese M, Ettinger M, Moffett A, et al. Prevention of bone loss in postmenopausal women treated with lasofoxifene compared with raloxifene. MENOPAUSE. 2006;13(3): 377-386.

https://doi.org/10.1097/01.gme.0000188736.69617.4f
54. Meunier PJ, Roux C, Ortolani S, Diaz-Curiel M, Compston J, Marquis P, et al. Effects of long-term strontium ranelate treatment on vertebral fracture risk in postmenopausal women with osteoporosis. Osteoporos Int. 2009;20(10): 1663-1673. https://doi.org/10.1007/s00198-008-0825-6
55. Miller PD, Chines AA, Christiansen C, Hoeck HC, Kendler DL, Lewiecki EM, et al. Effects of bazedoxifene on BMD and bone turnover in postmenopausal women: 2-yr results of a randomized, double-blind, placebo-, and active-controlled study. J BONE MINER RES. 2008;23(4): 525-535. https://doi.org/10.1359/jbmr.071206
56. Rosen CJ, Hochberg MC, Bonnick SL, McClung M, Miller P, Broy S, et al. Treatment with once-weekly alendronate 70 mg compared with once-weekly risedronate 35 mg in women with postmenopausal osteoporosis: a randomized double-blind study. J BONE MINER RES. 2005;20(1): 141-151. https://doi.org/10.1359/JBMR.040920
57. Valimaki MJ, Farrerons-Minguella J, Halse J, Kroger H, Maroni M, Mulder H, et al. Effects of risedronate 5 mg/d on bone mineral density and bone turnover markers in late-postmenopausal women with osteopenia: a multinational, 24-month, randomized, double-blind, placebo-controlled, parallel-group, phase III trial. CLIN THER. 2007;29(9): 1937-1949. https://doi.org/10.1016/j.clinthera.2007.09.017
