# Supplementary figures and images for: Clinical efficacy and safety of drug interventions for primary and secondary prevention of osteoporotic fractures in postmenopausal women: Network meta-analysis followed by factor and cluster analysis
Source: PLoS One. 2020 Jun 3;15(6):e0234123. doi: 10.1371/journal.pone.0234123 (PMC7269244; doi:10.1371/journal.pone.0234123)

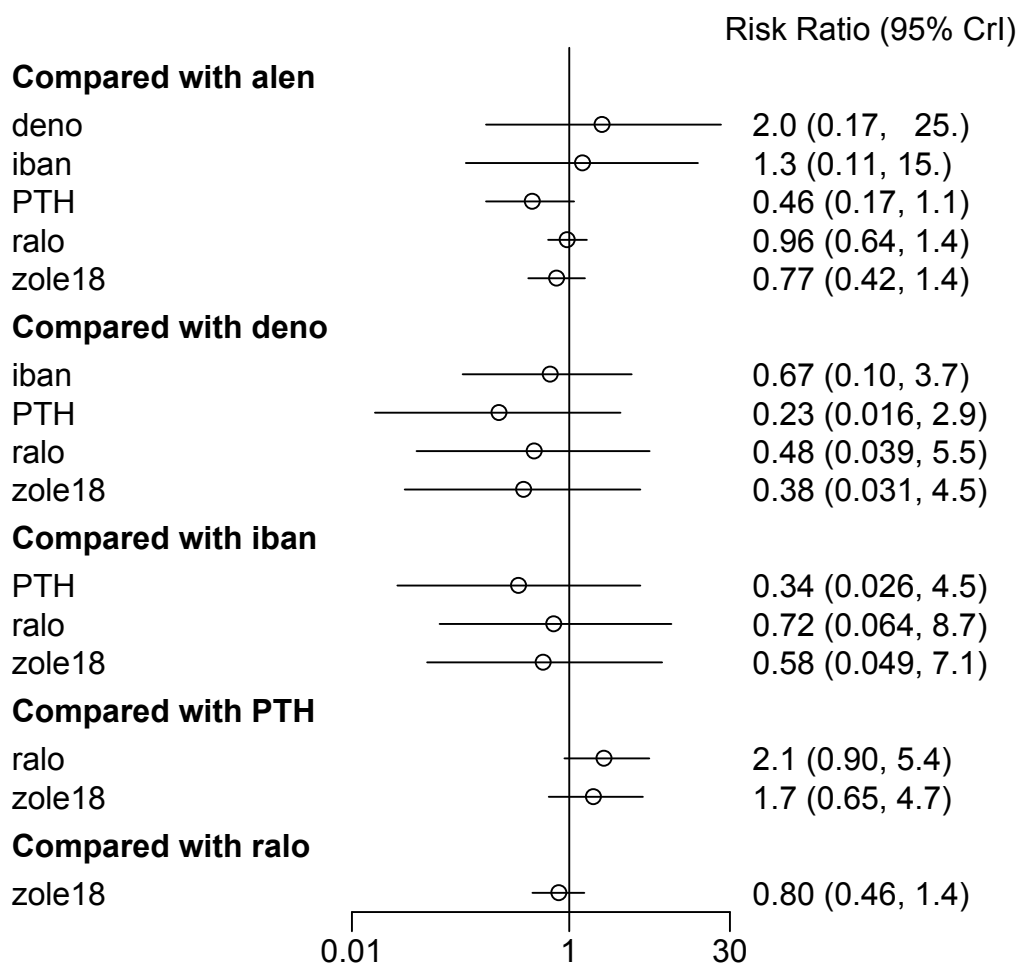

Supplement: S5 Appendix — (PDF) [file pone.0234123.s005.pdf]

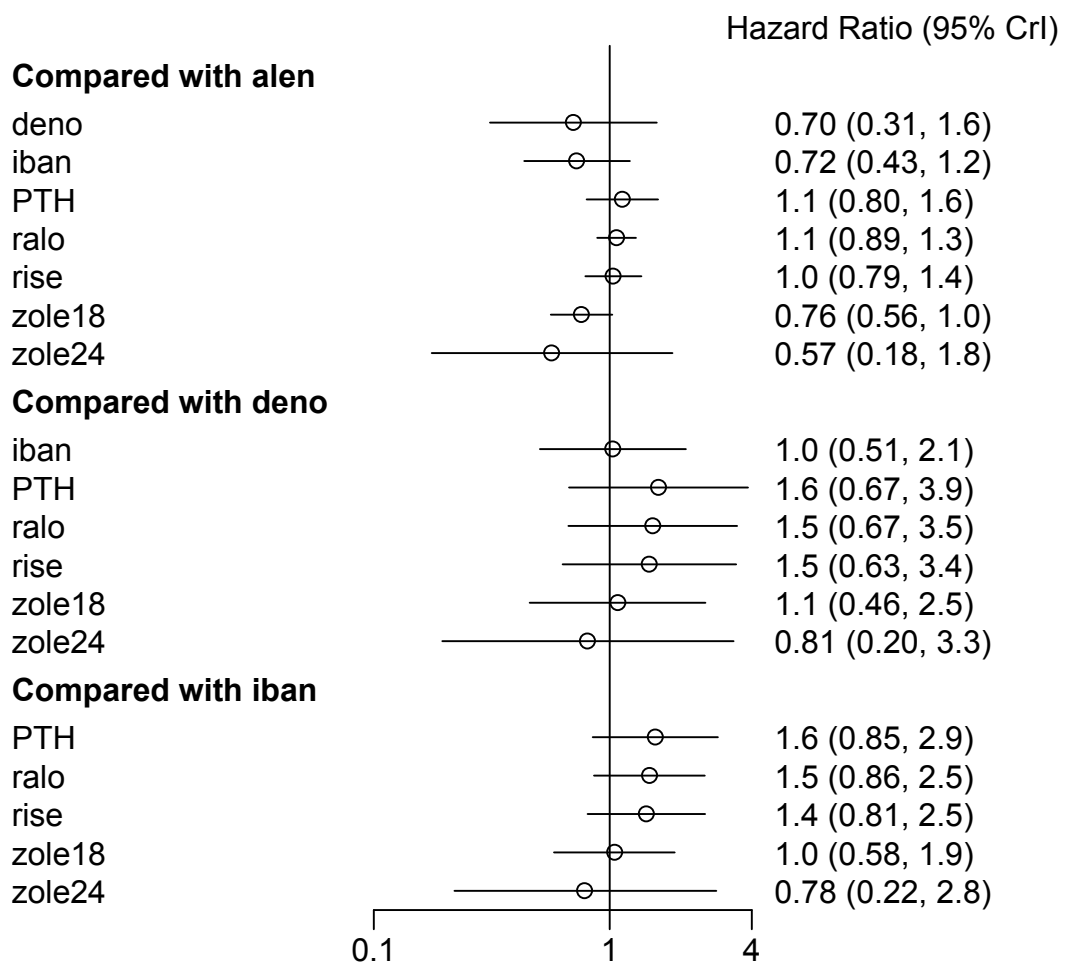

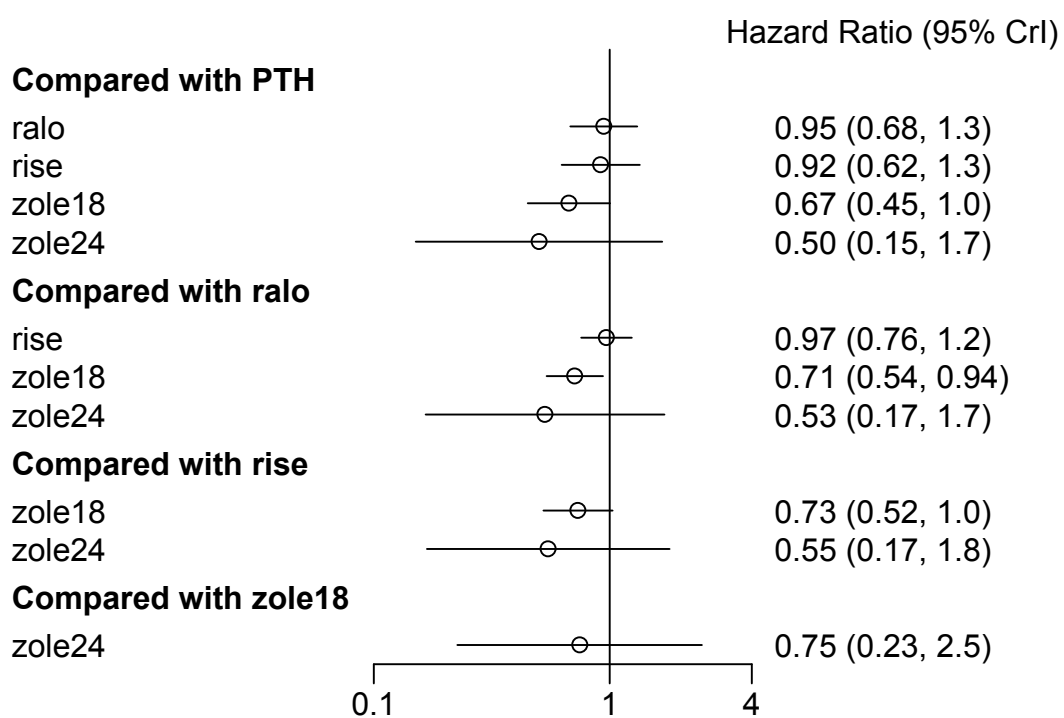

Supplement: S6 Appendix — (PDF) [file pone.0234123.s006.pdf]

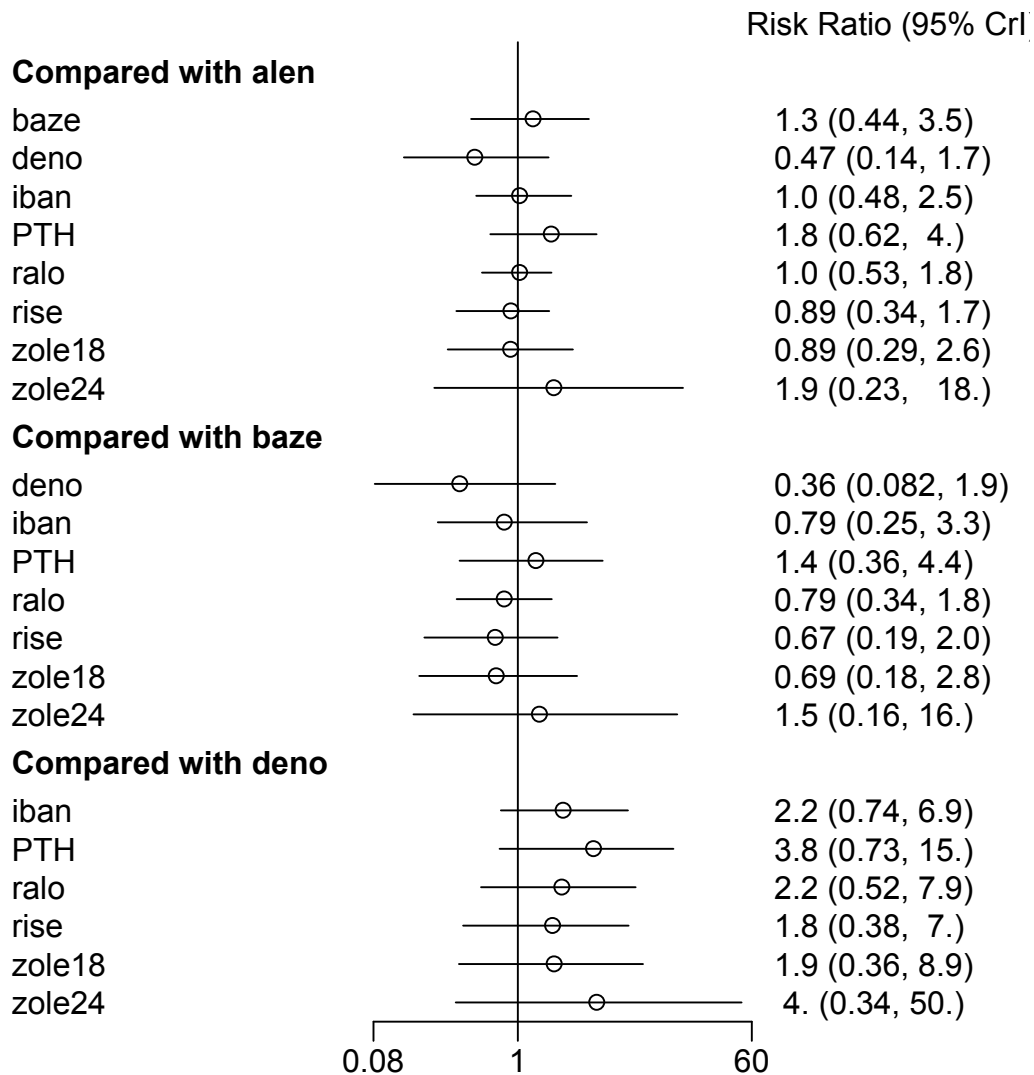

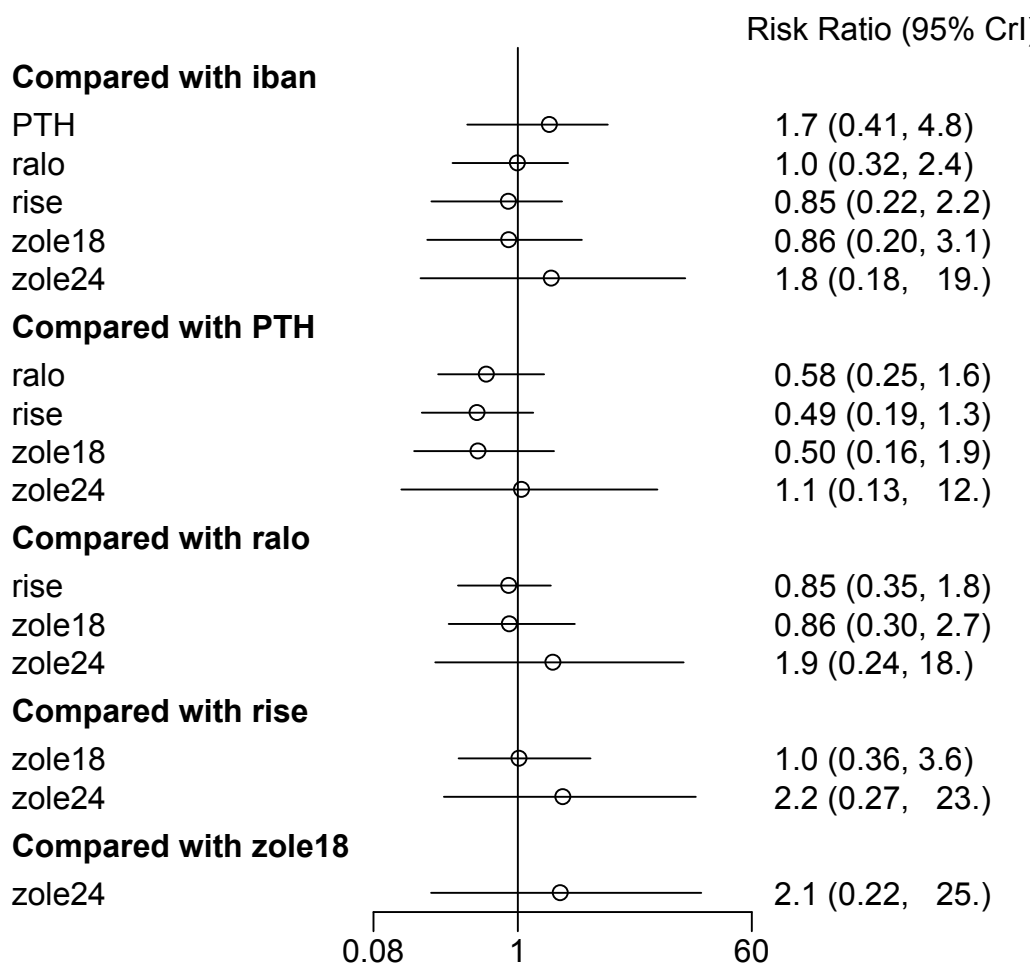

Supplement: S7 Appendix — (PDF) [file pone.0234123.s007.pdf]

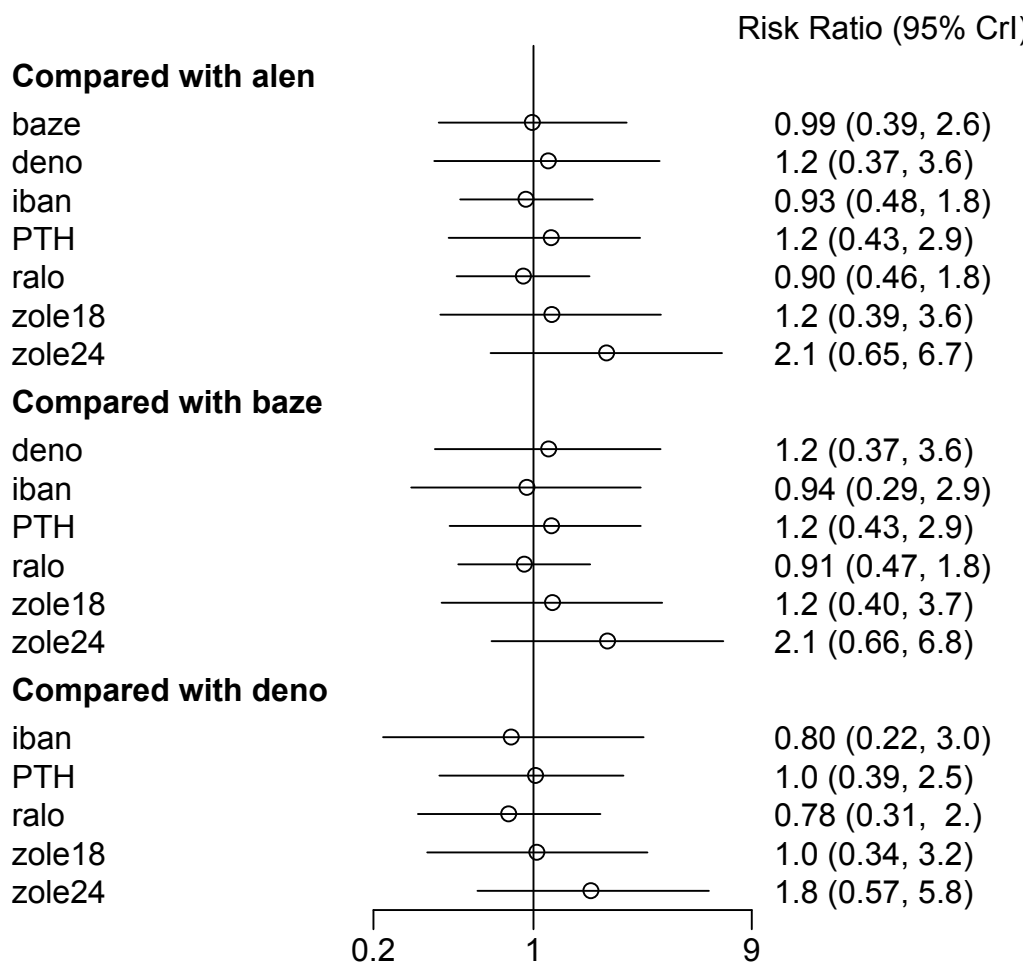

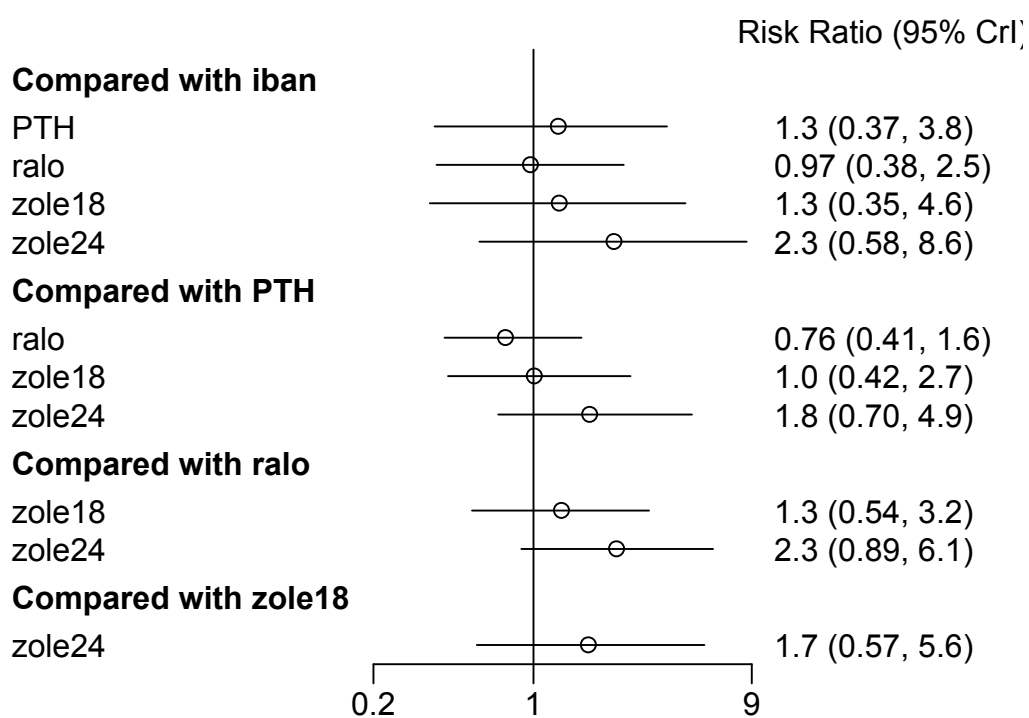

Supplement: S8 Appendix — (PDF) [file pone.0234123.s008.pdf]

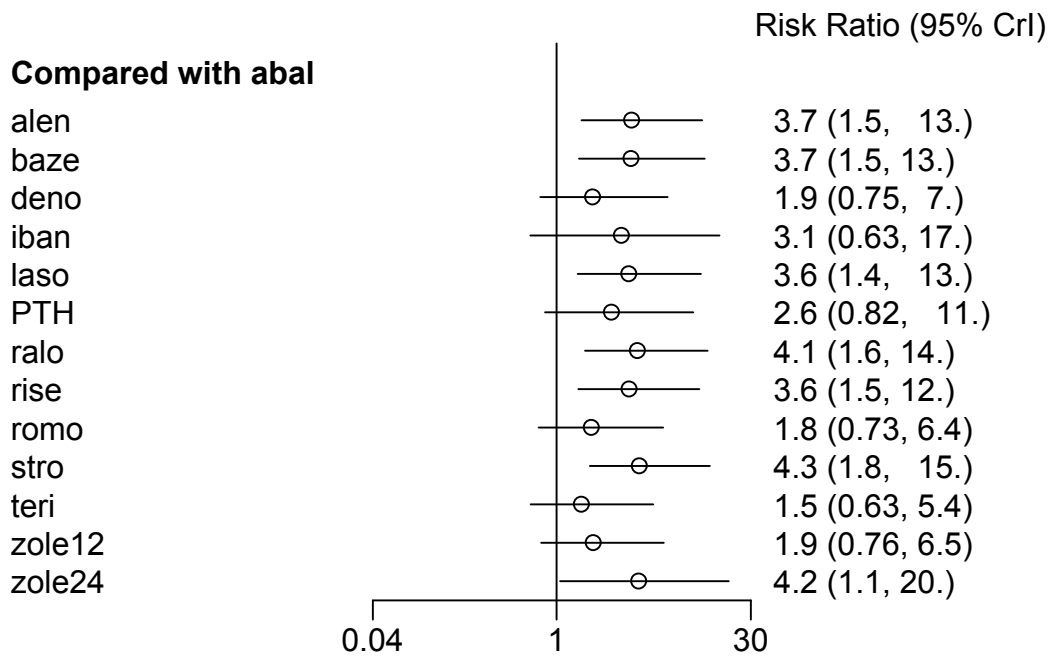

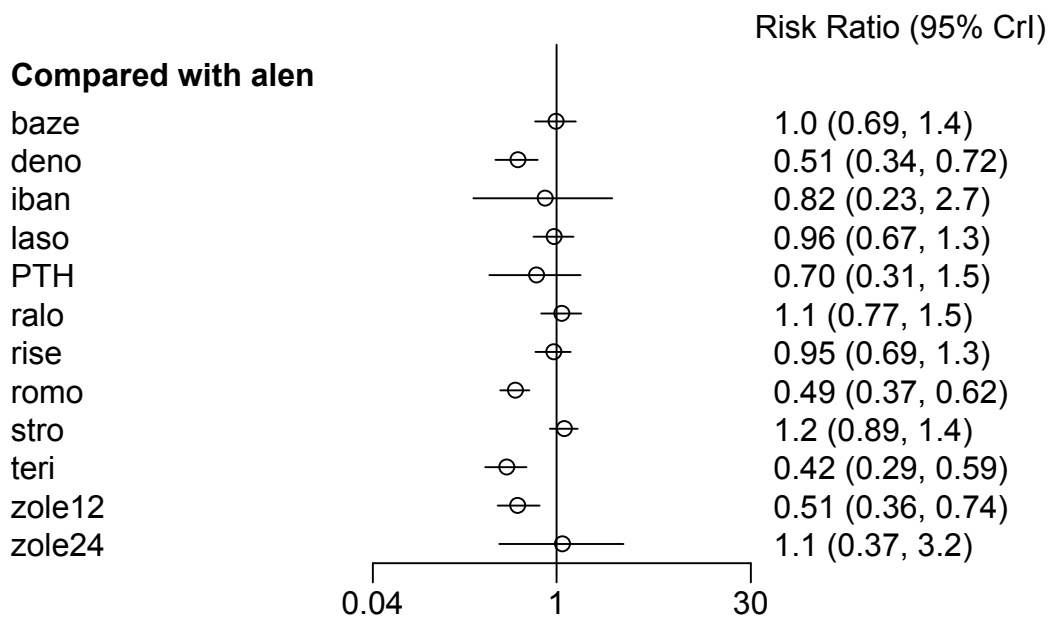

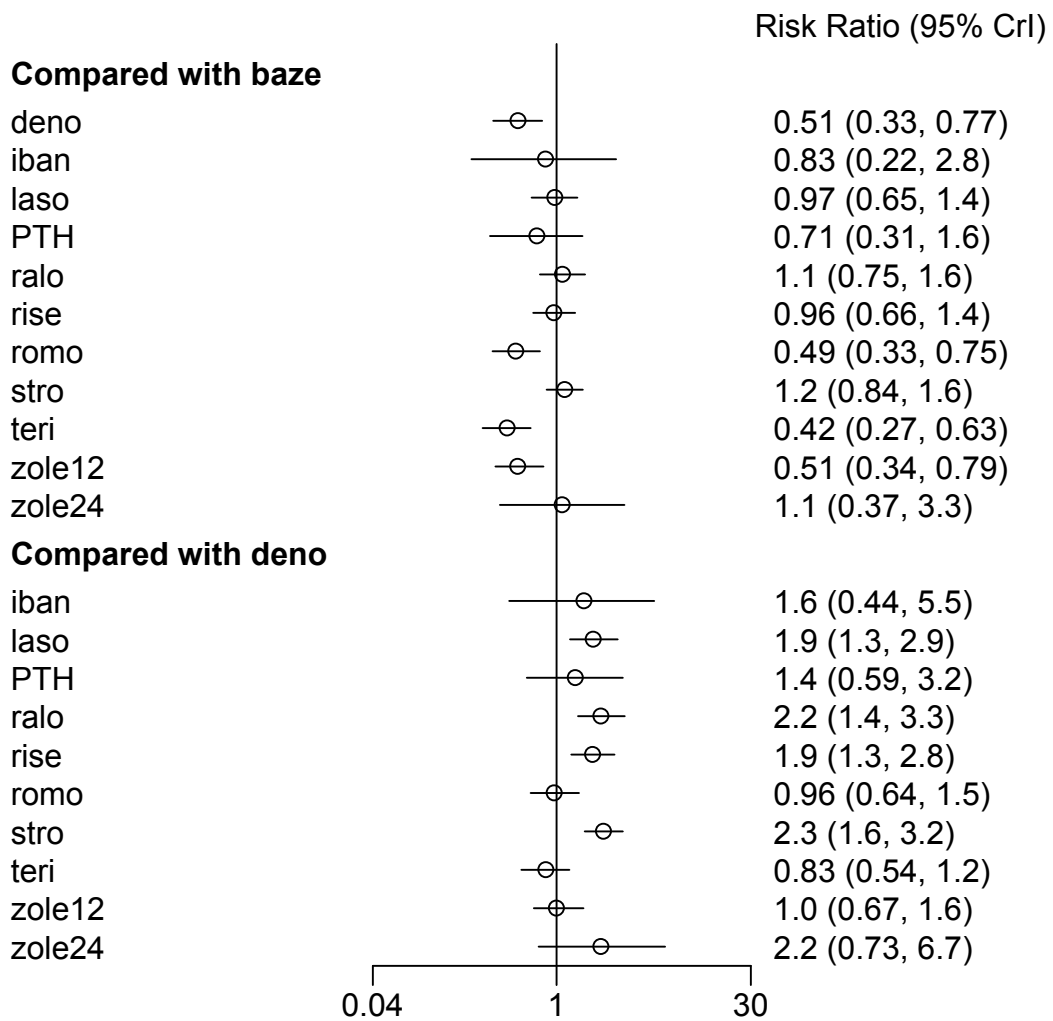

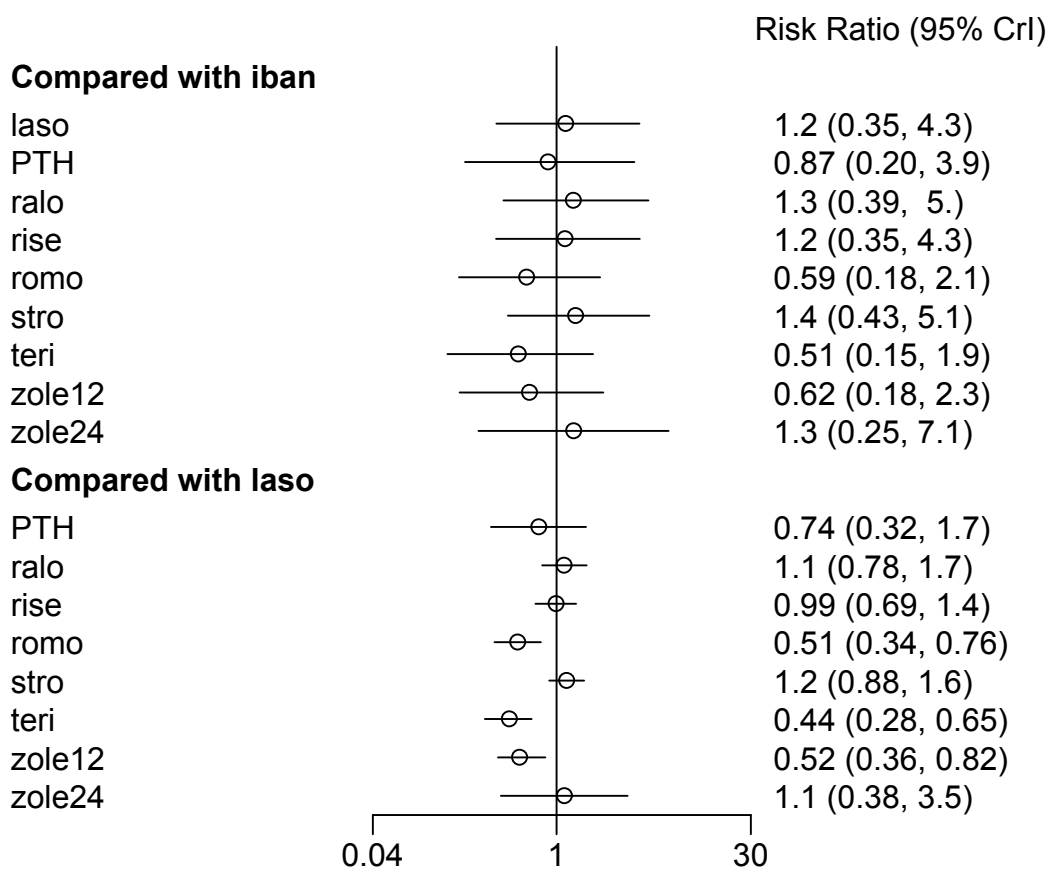

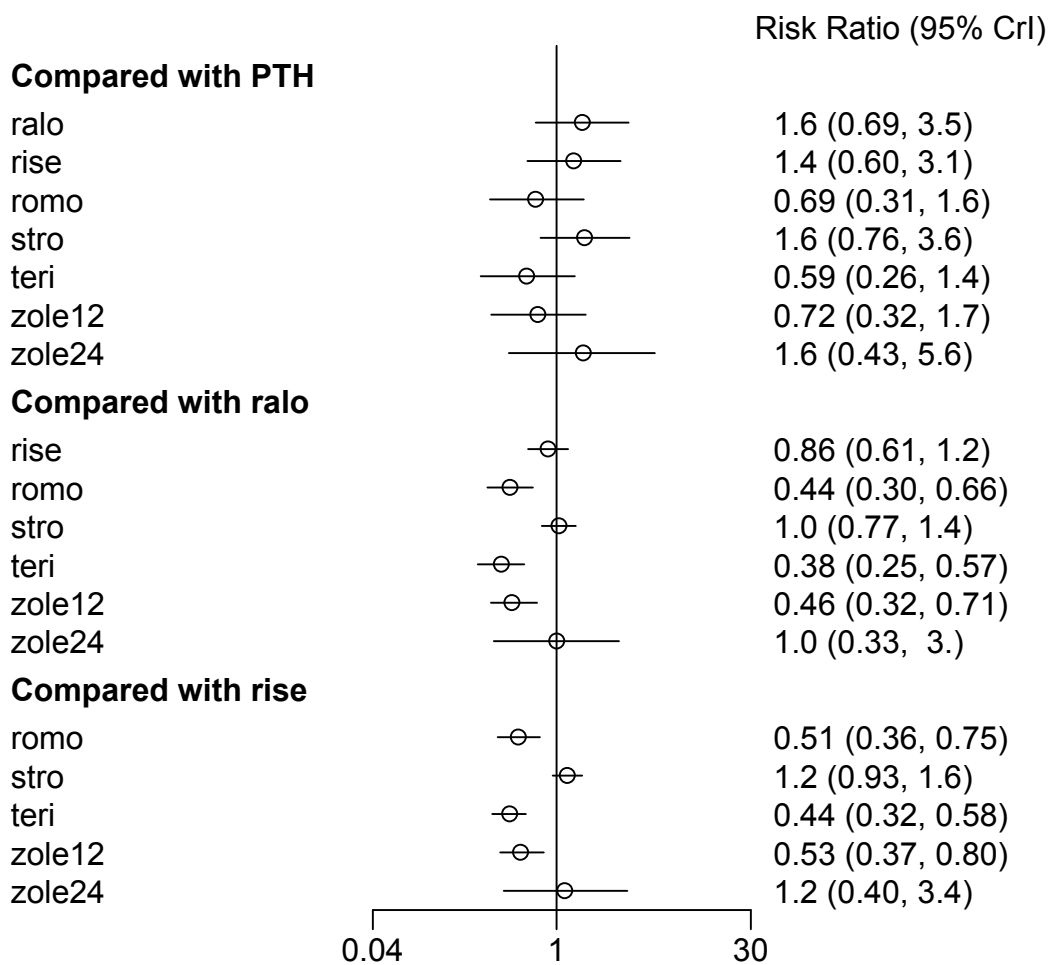

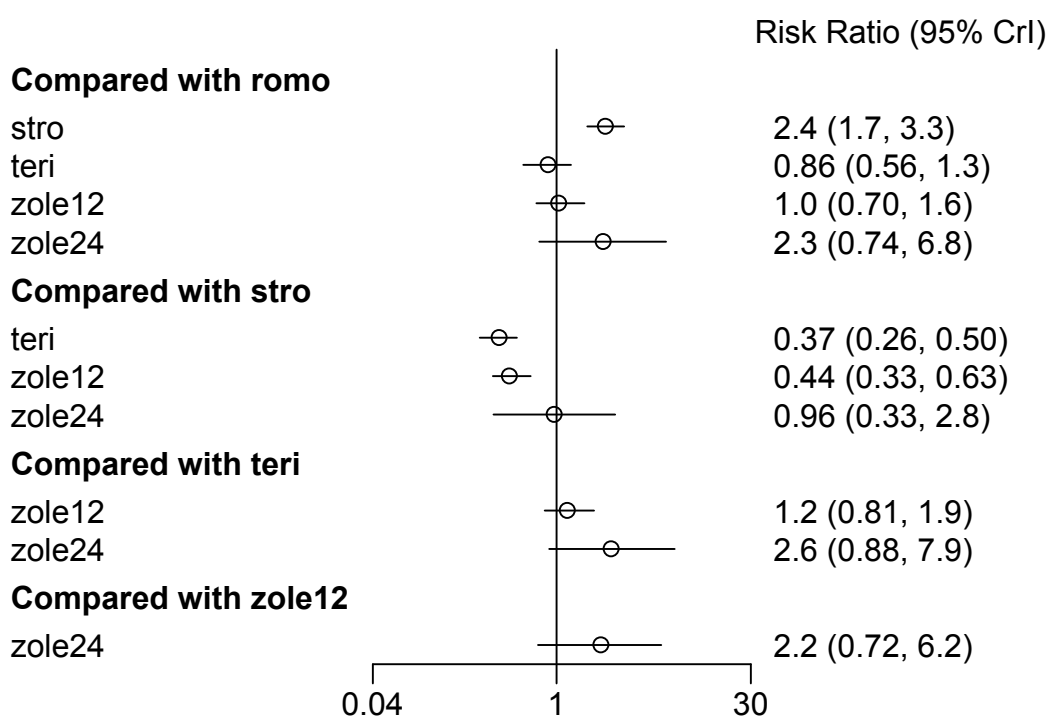

Supplement: S9 Appendix — (PDF) [file pone.0234123.s009.pdf]

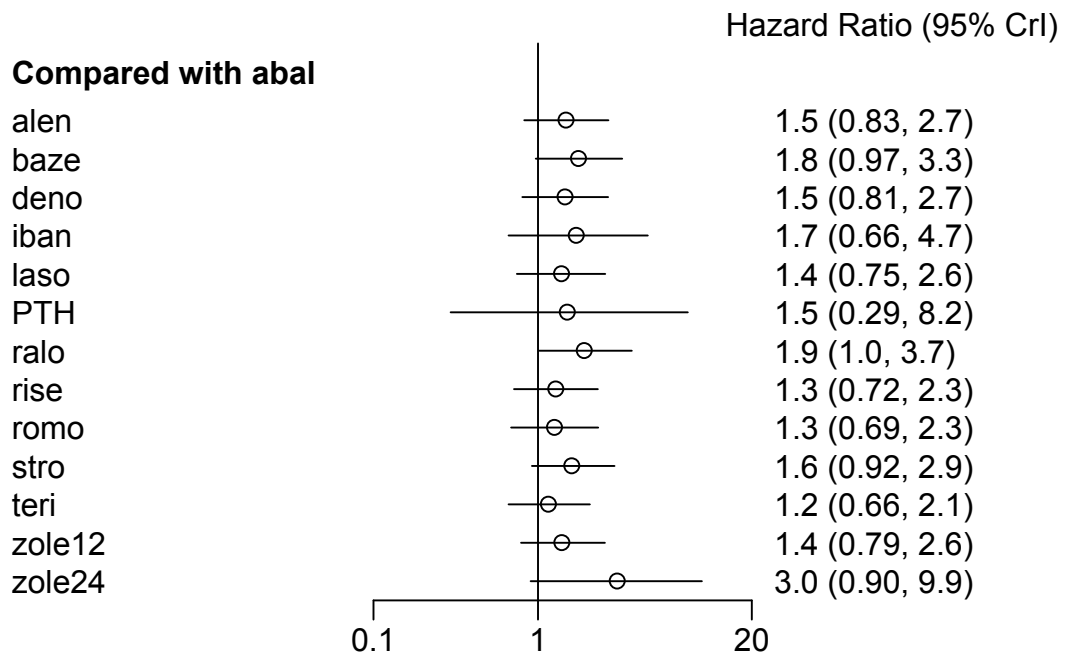

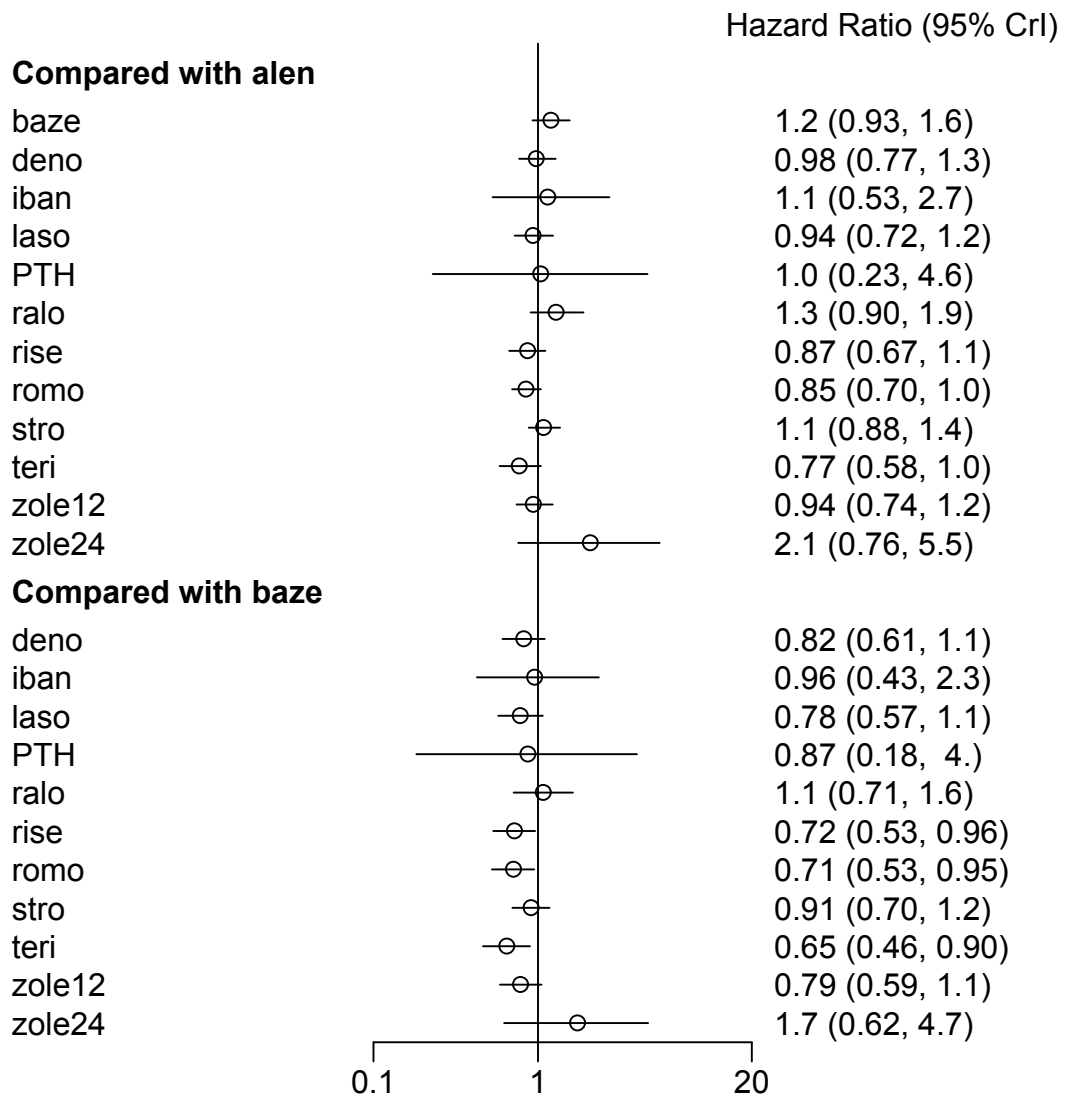

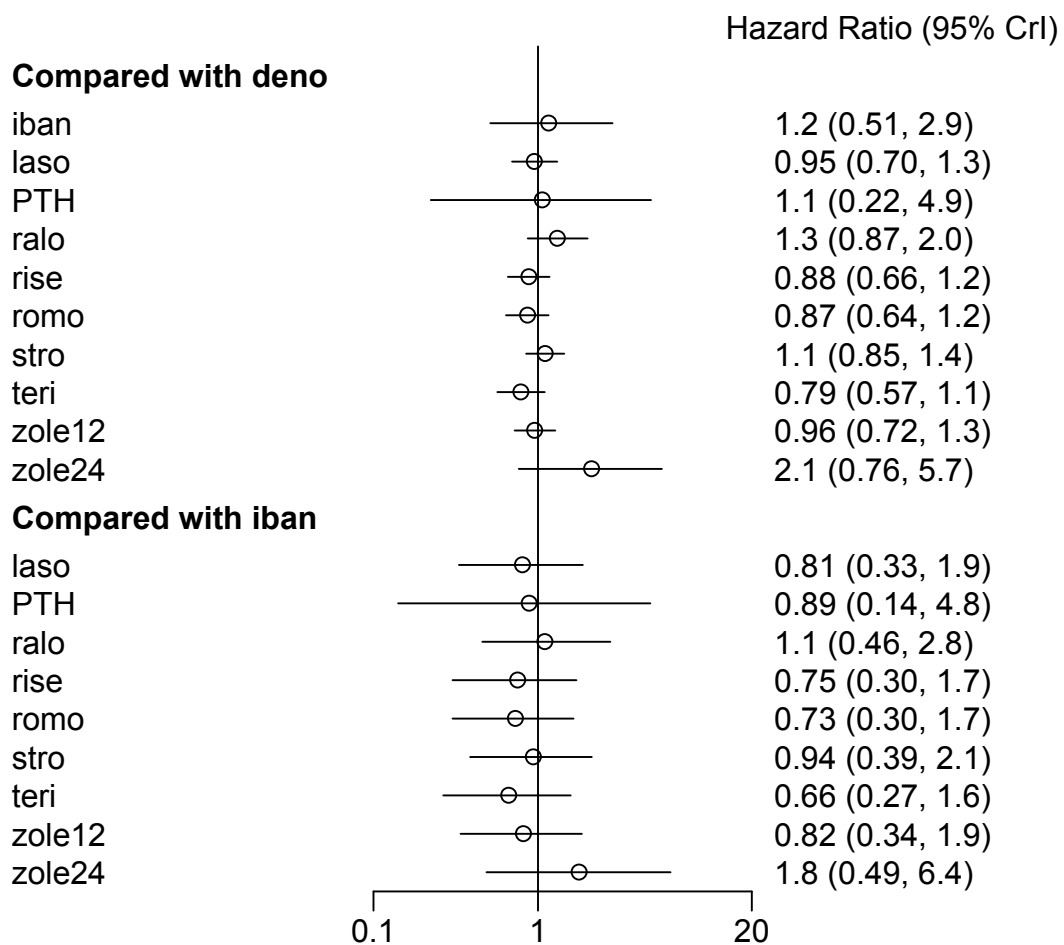

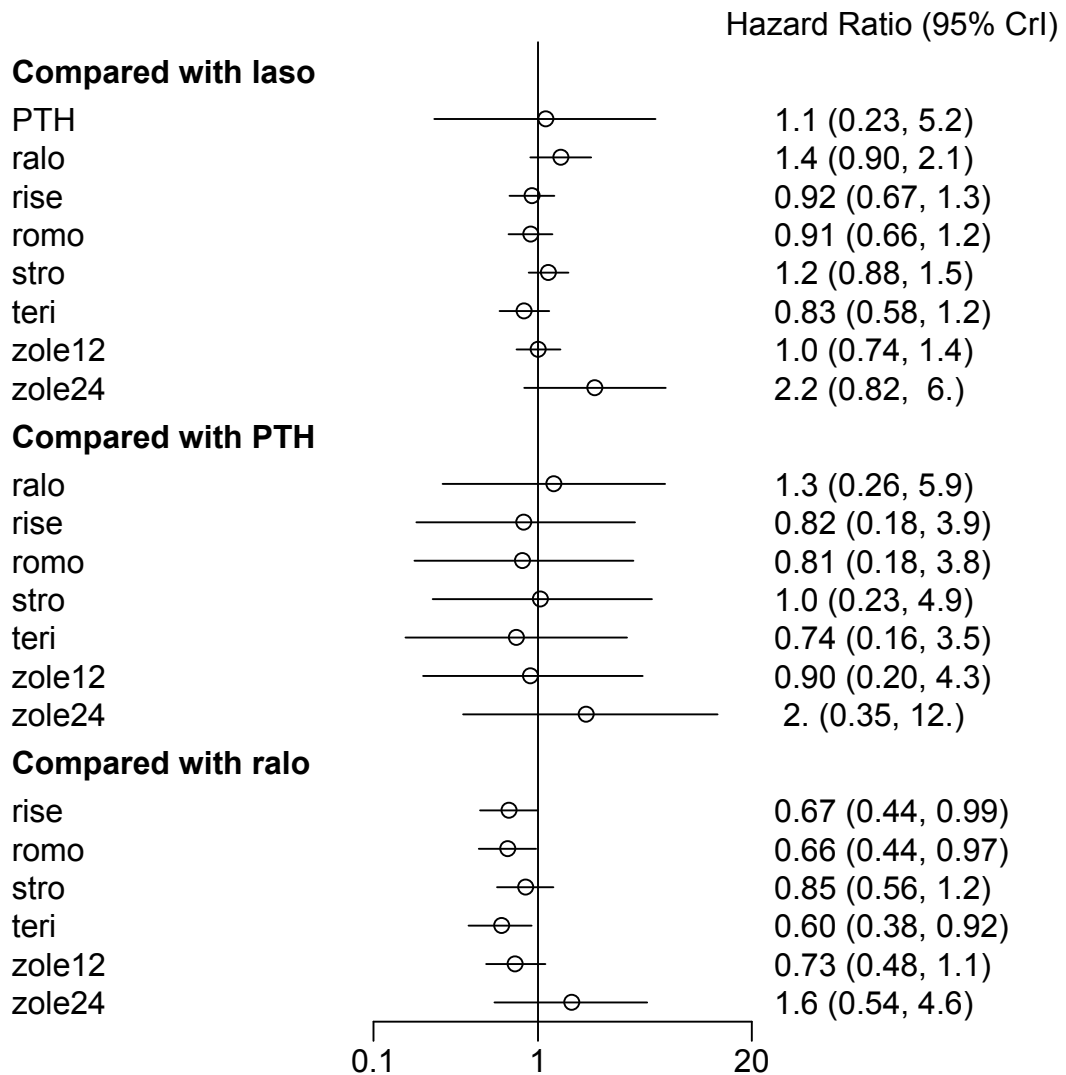

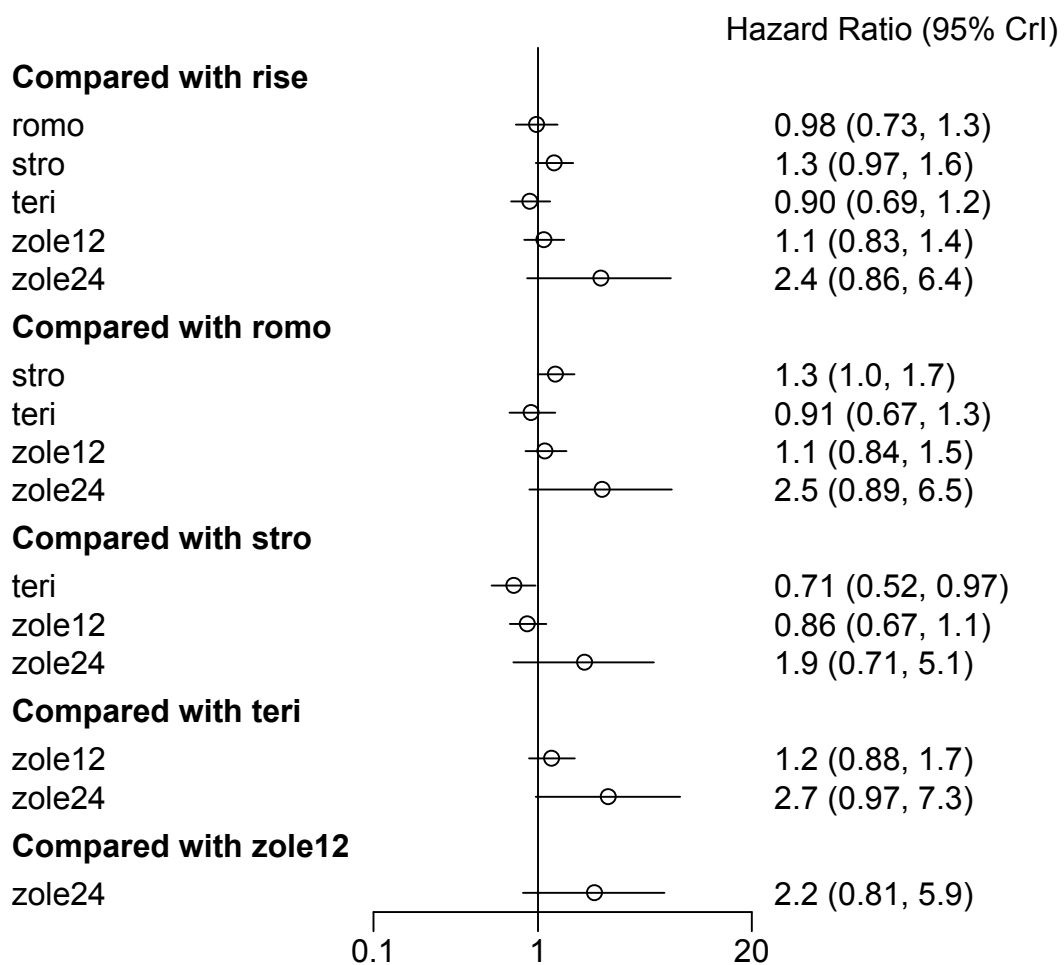

Supplement: S10 Appendix — (PDF) [file pone.0234123.s010.pdf]

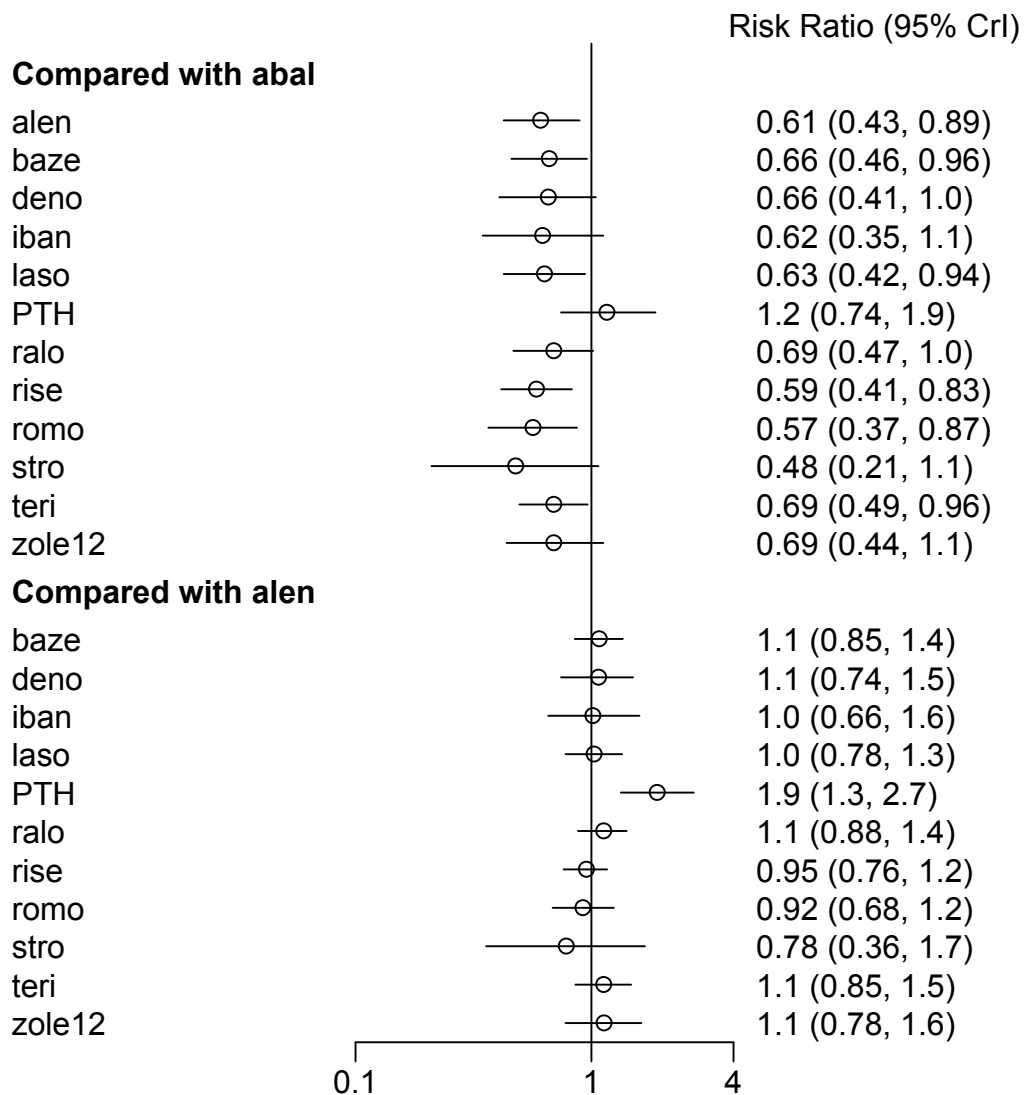

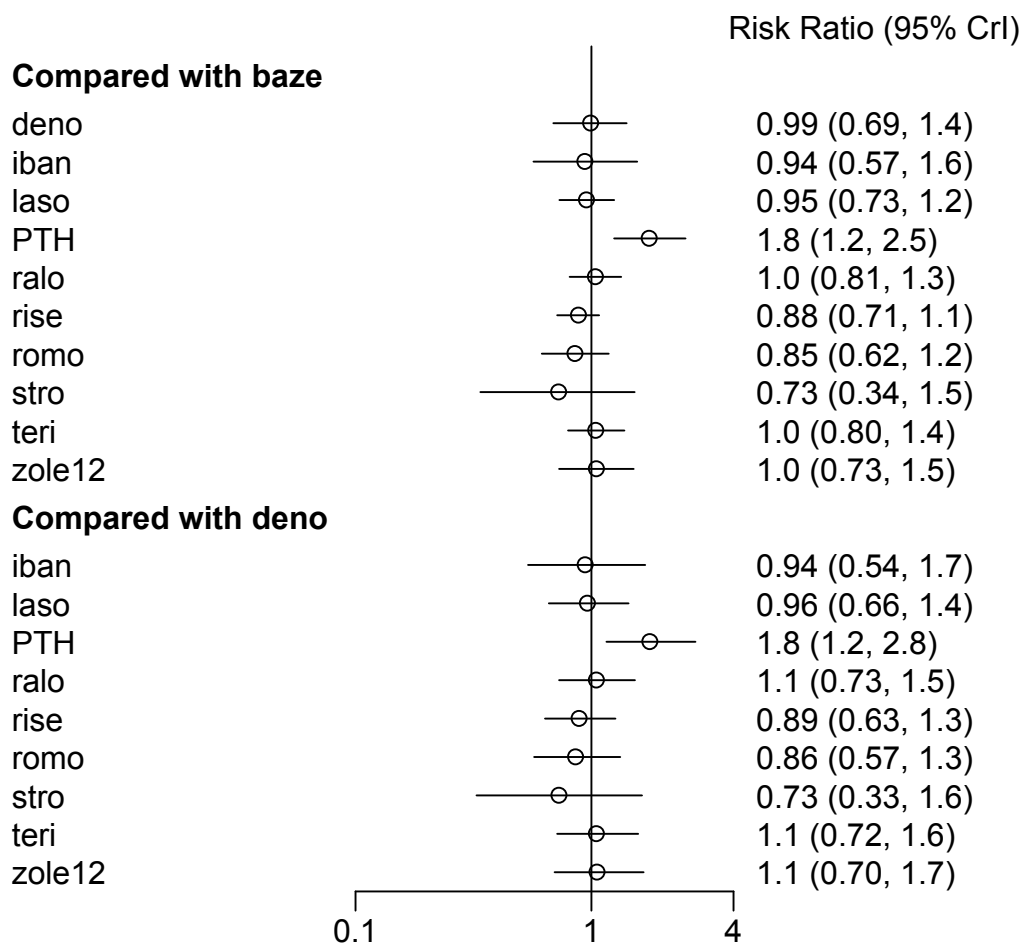

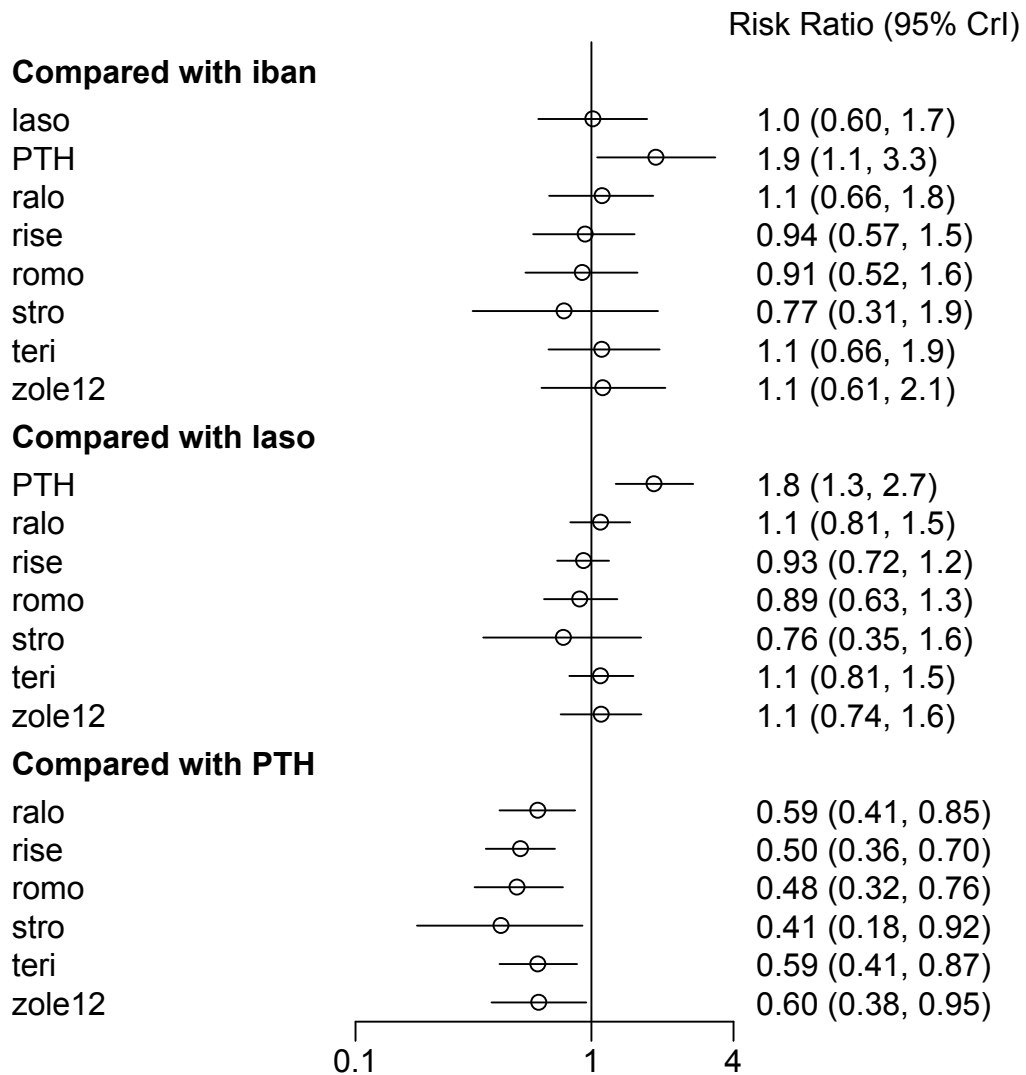

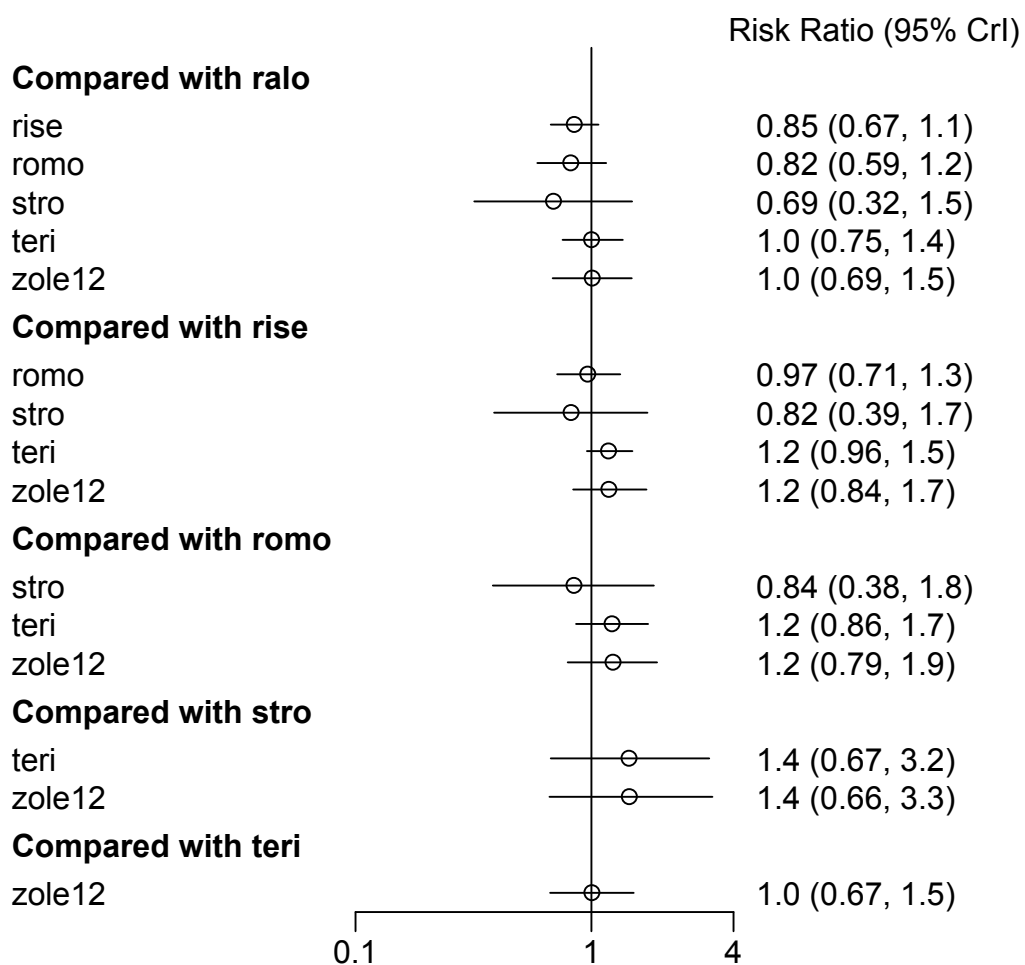

Supplement: S11 Appendix — (PDF) [file pone.0234123.s011.pdf]

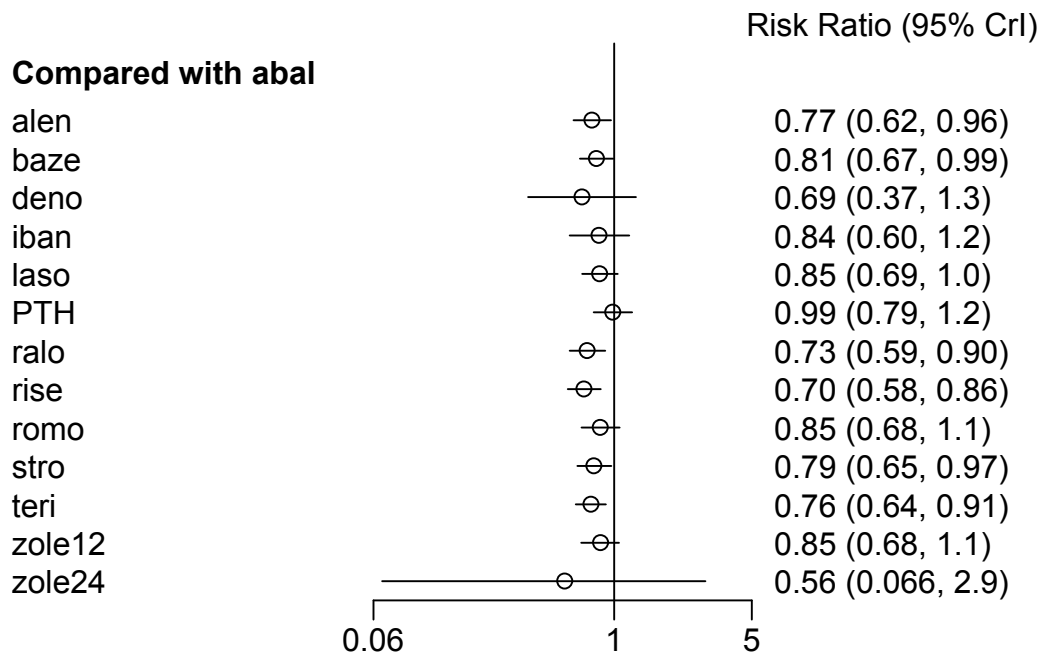

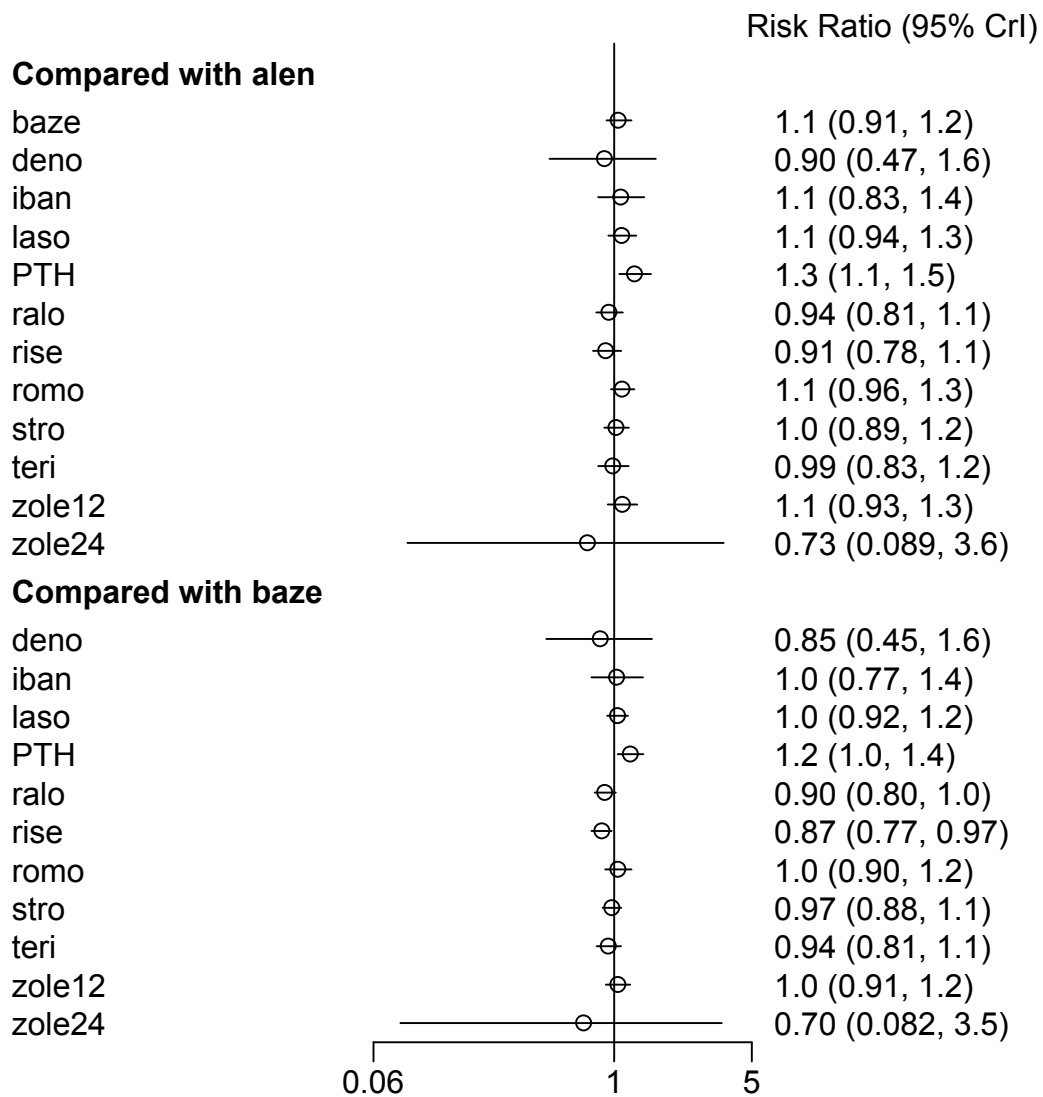

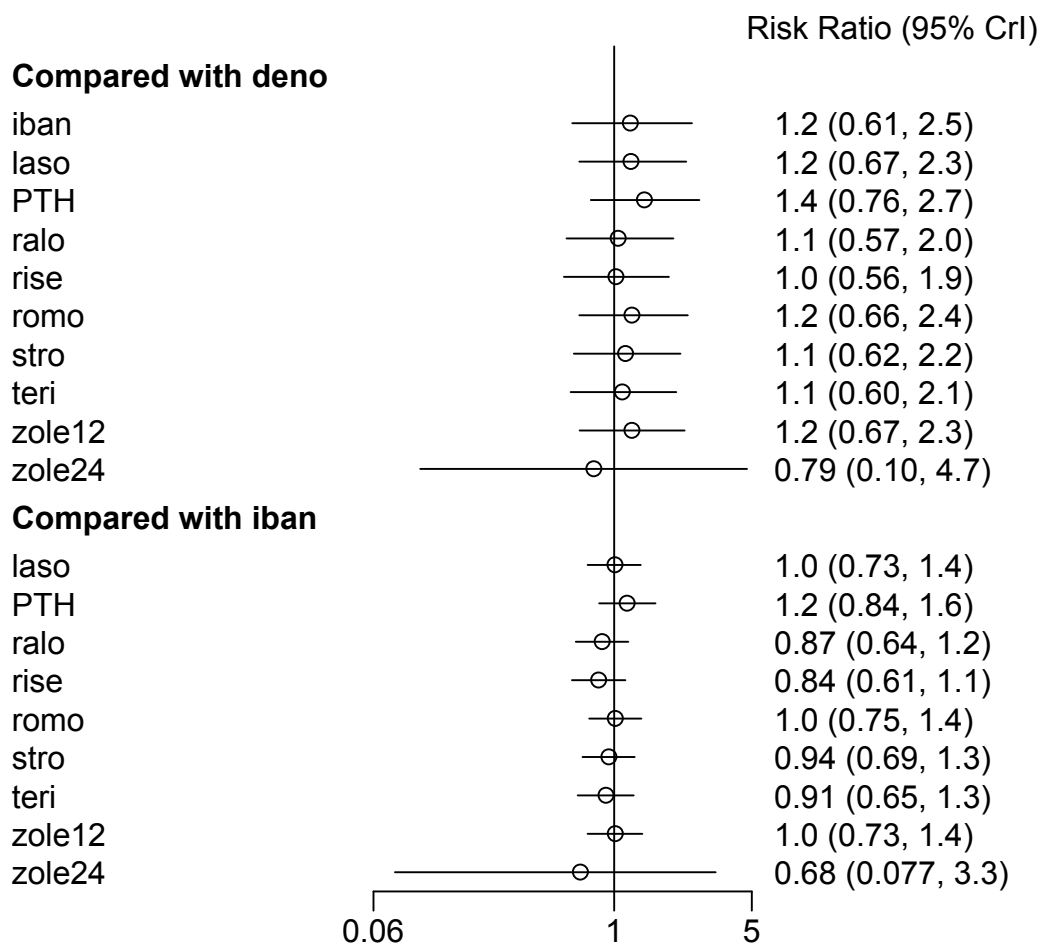

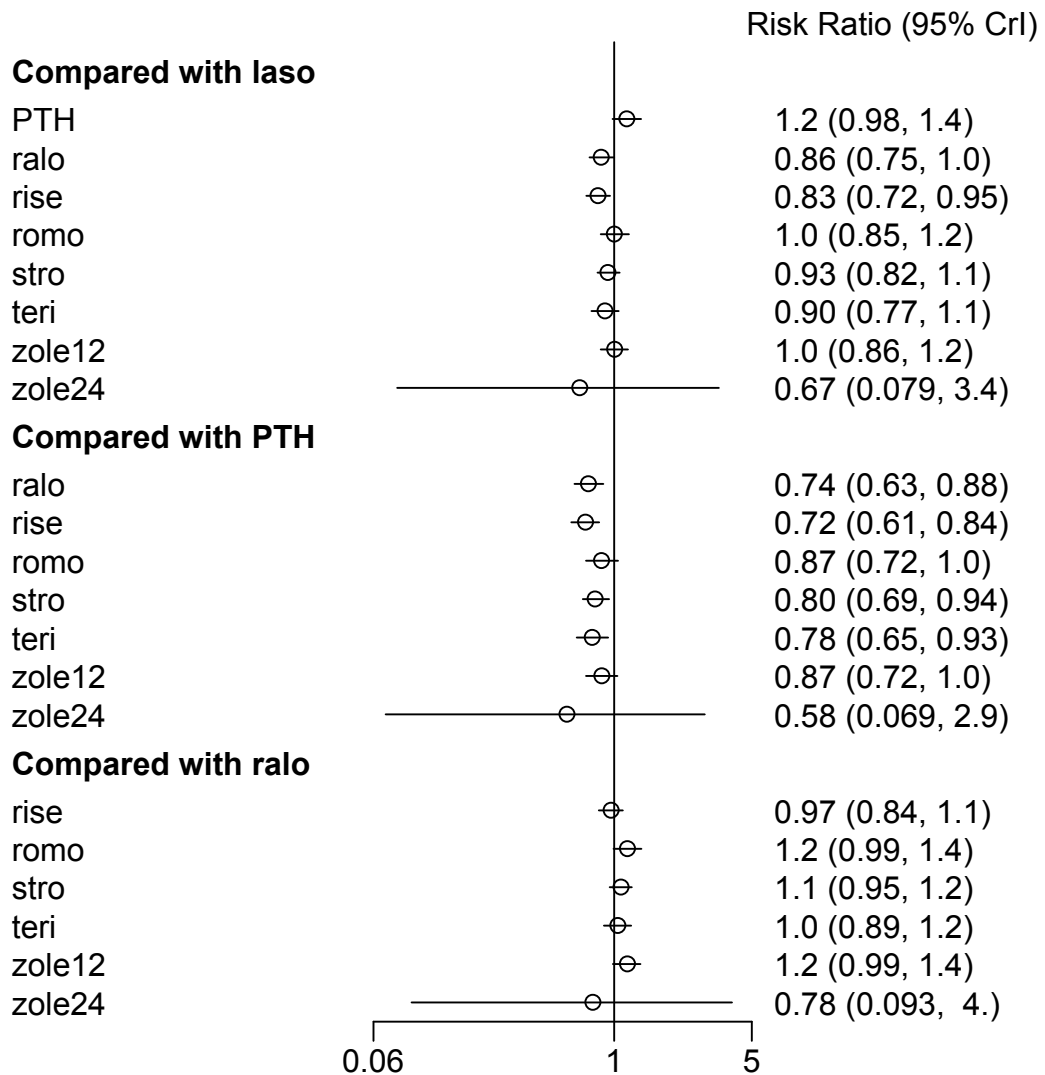

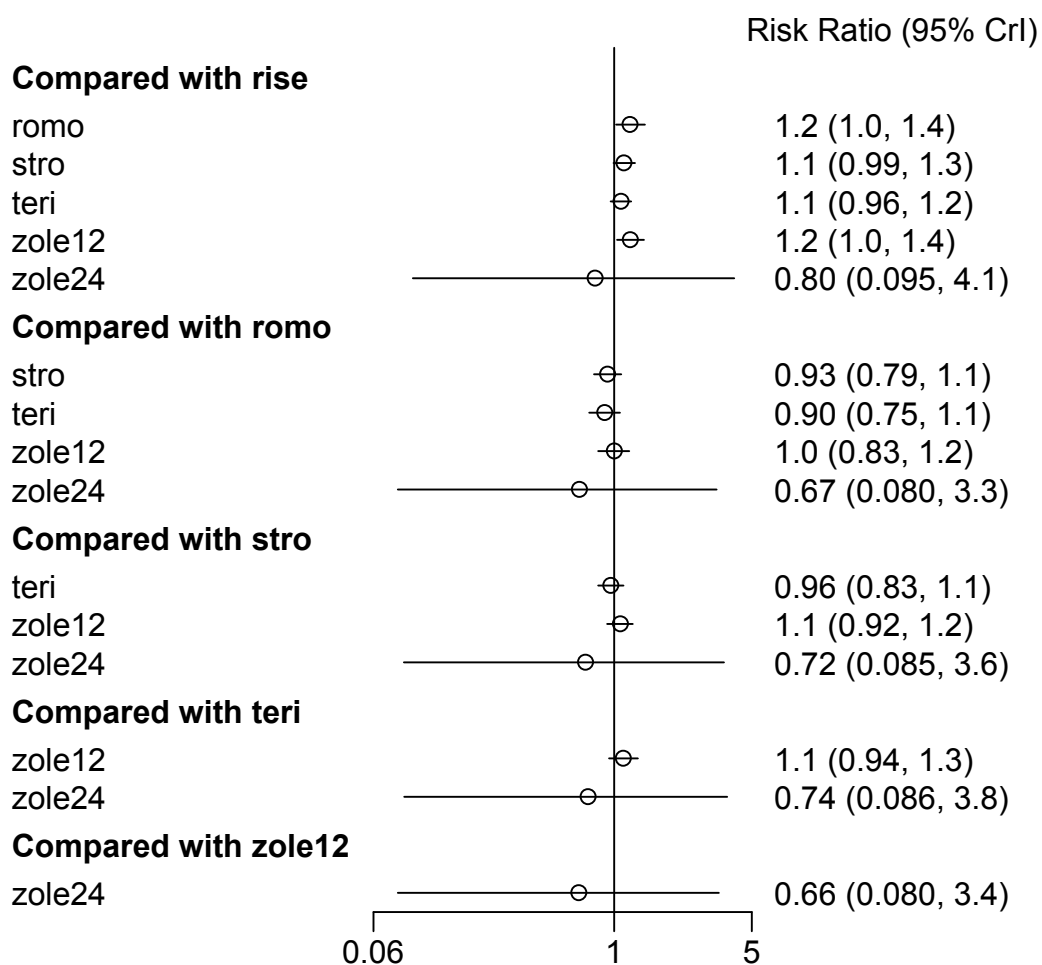

Supplement: S12 Appendix — (PDF) [file pone.0234123.s012.pdf]

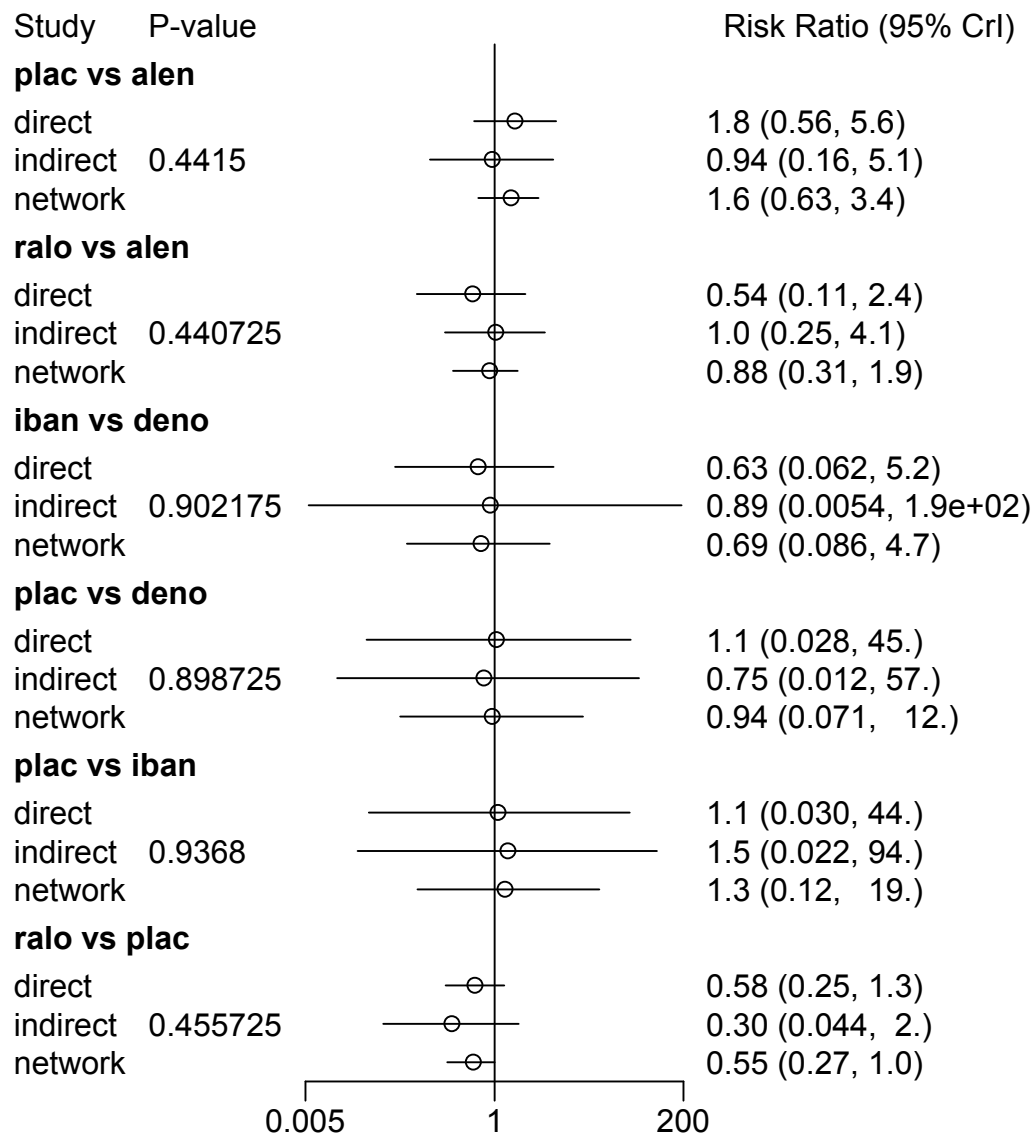

Supplement: S13 Appendix — (PDF) [file pone.0234123.s013.pdf]

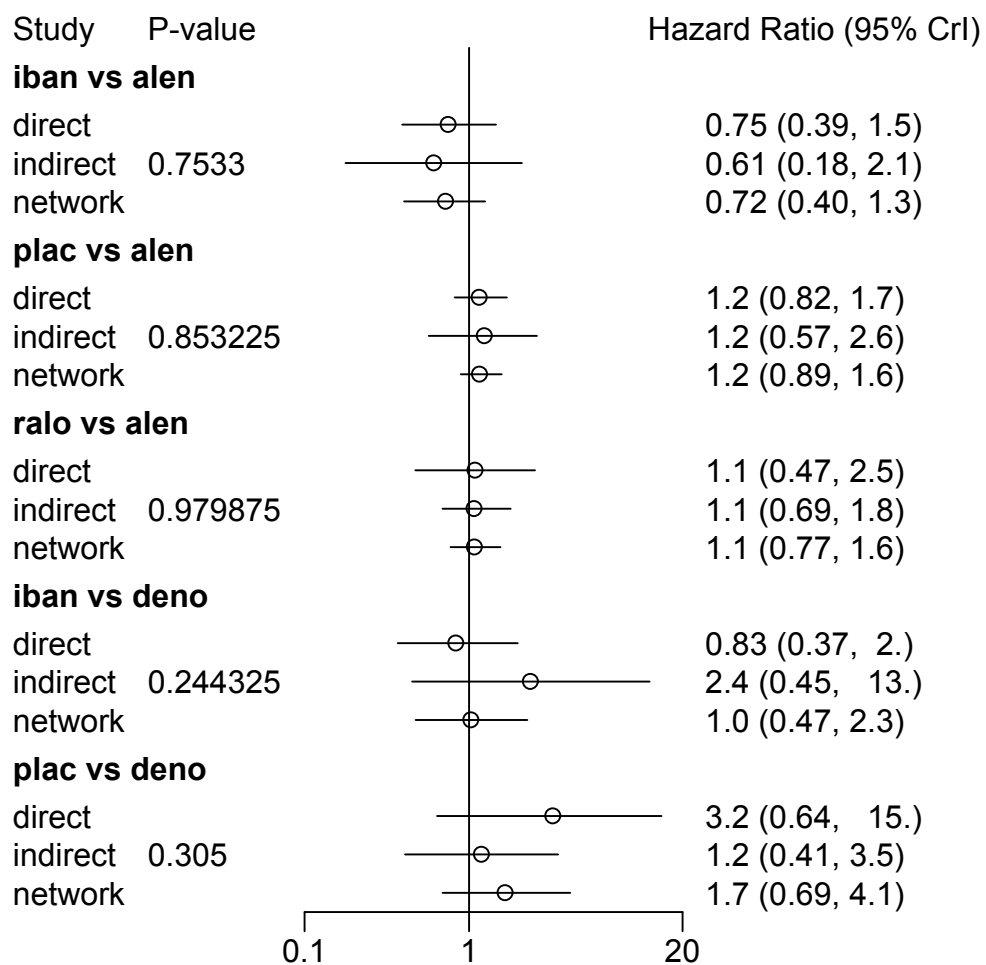

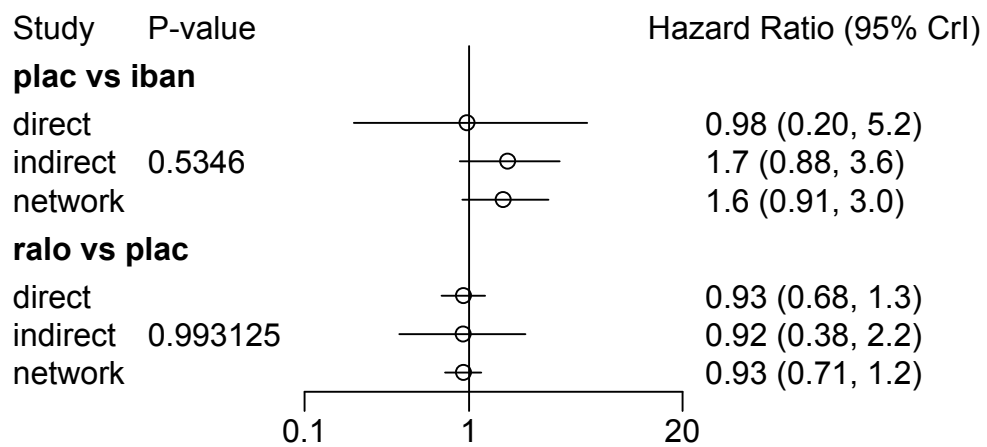

Supplement: S14 Appendix — (PDF) [file pone.0234123.s014.pdf]

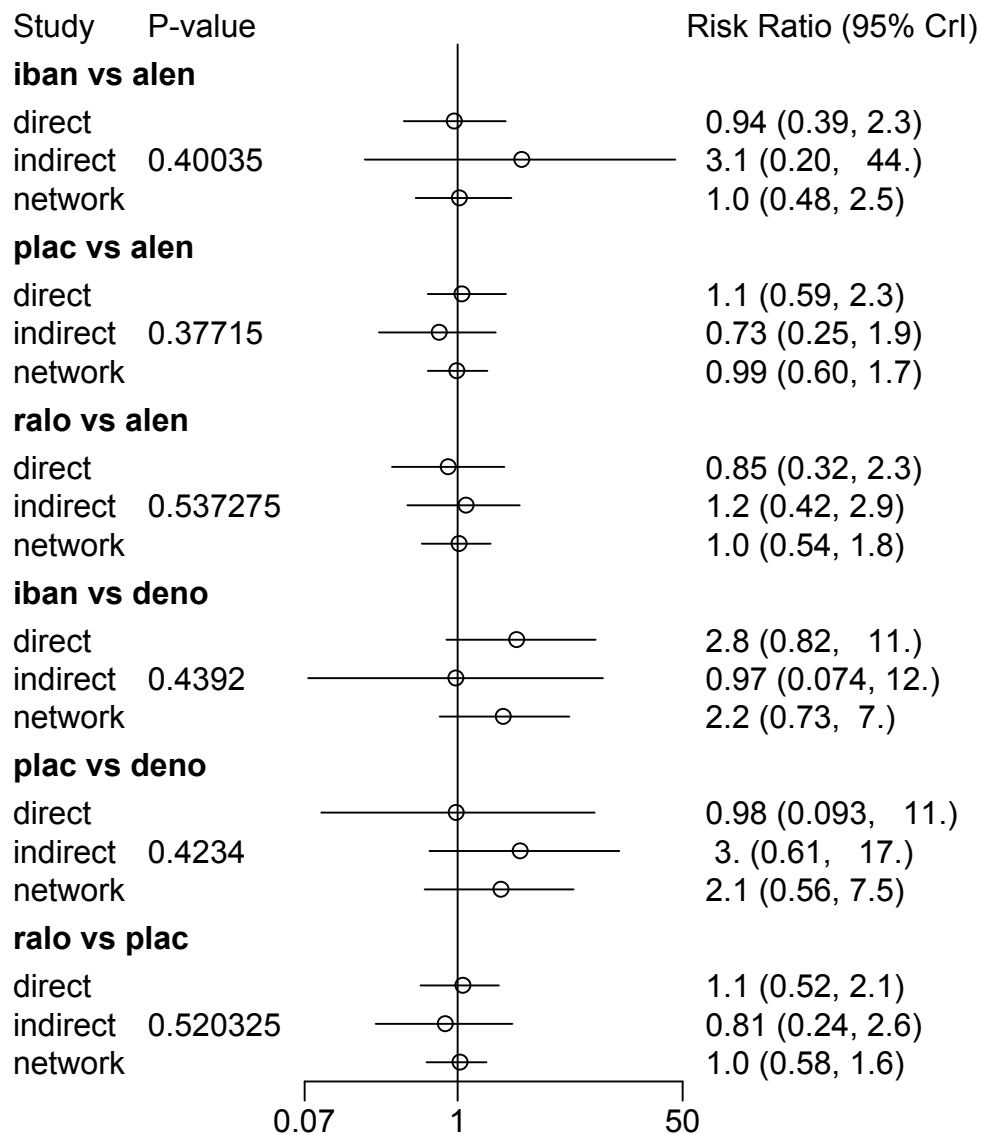

Supplement: S15 Appendix — (PDF) [file pone.0234123.s015.pdf]

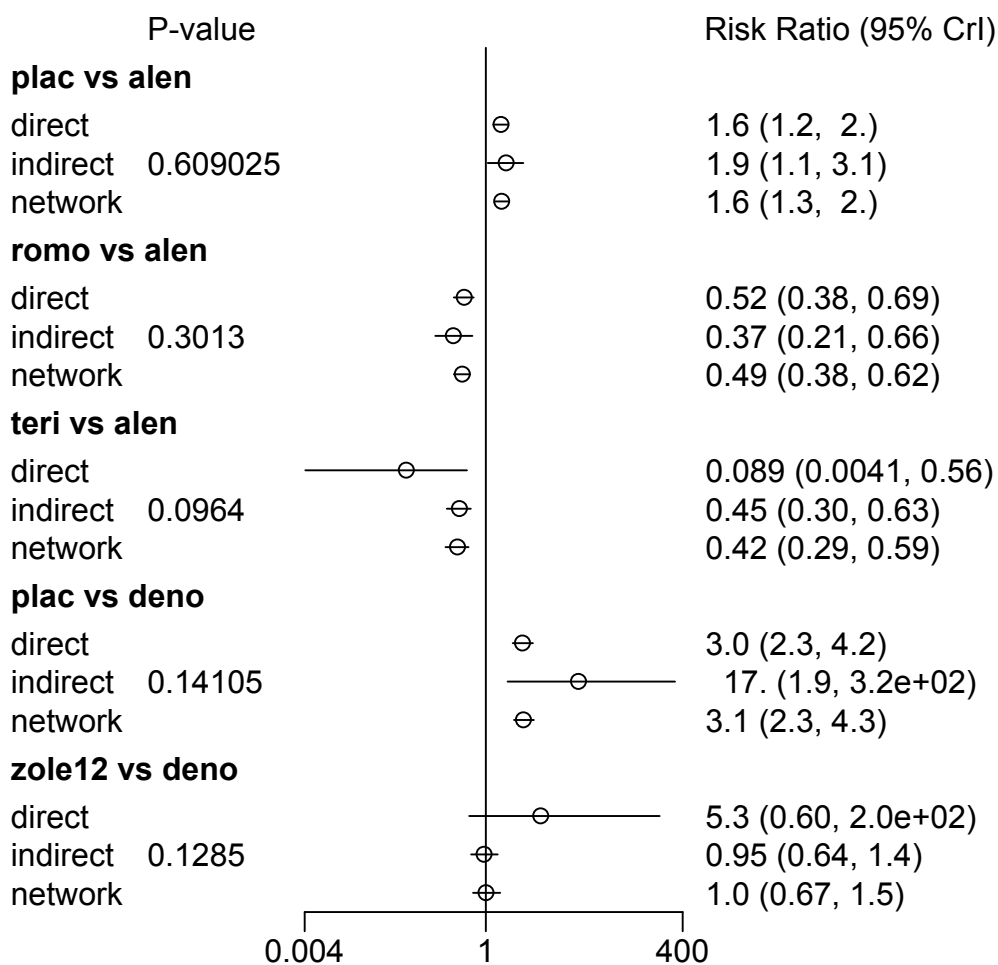

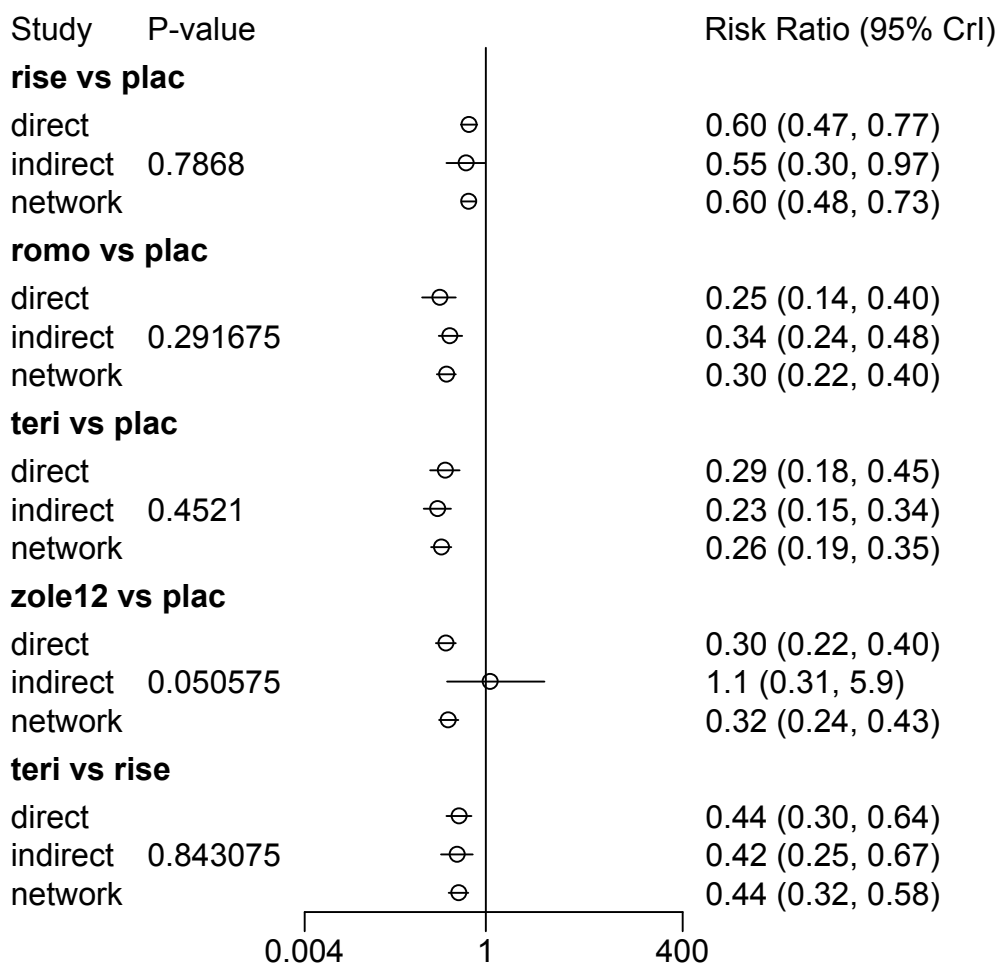

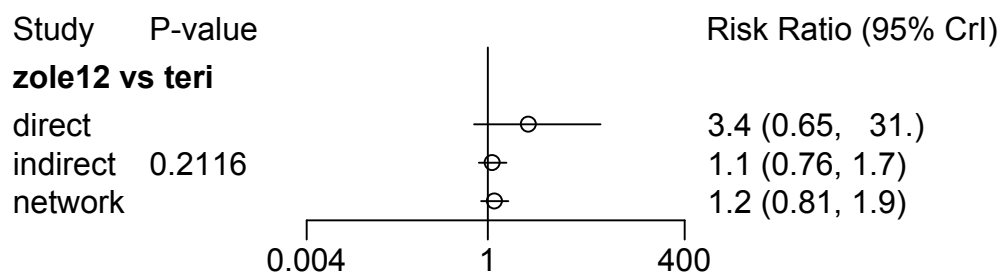

Supplement: S16 Appendix — (PDF) [file pone.0234123.s016.pdf]

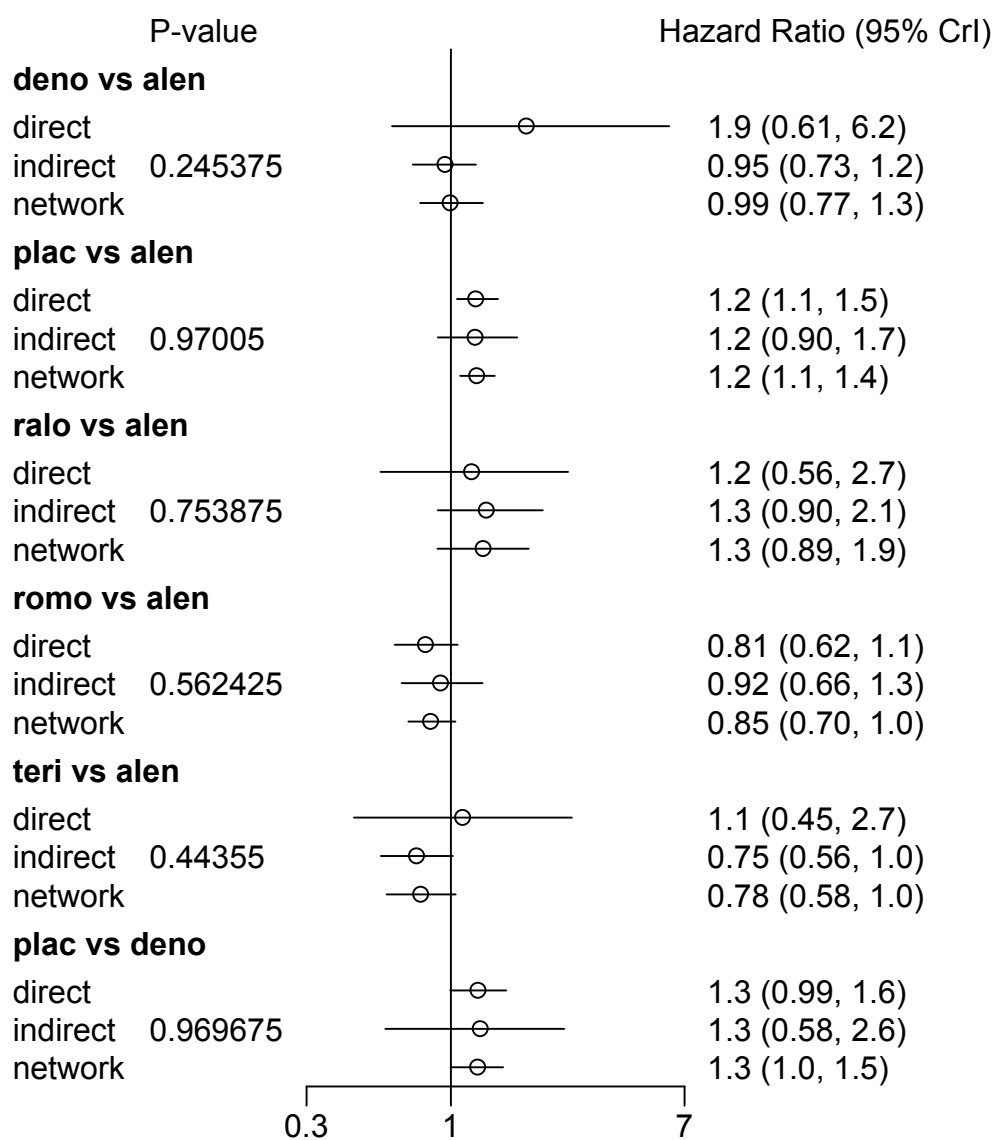

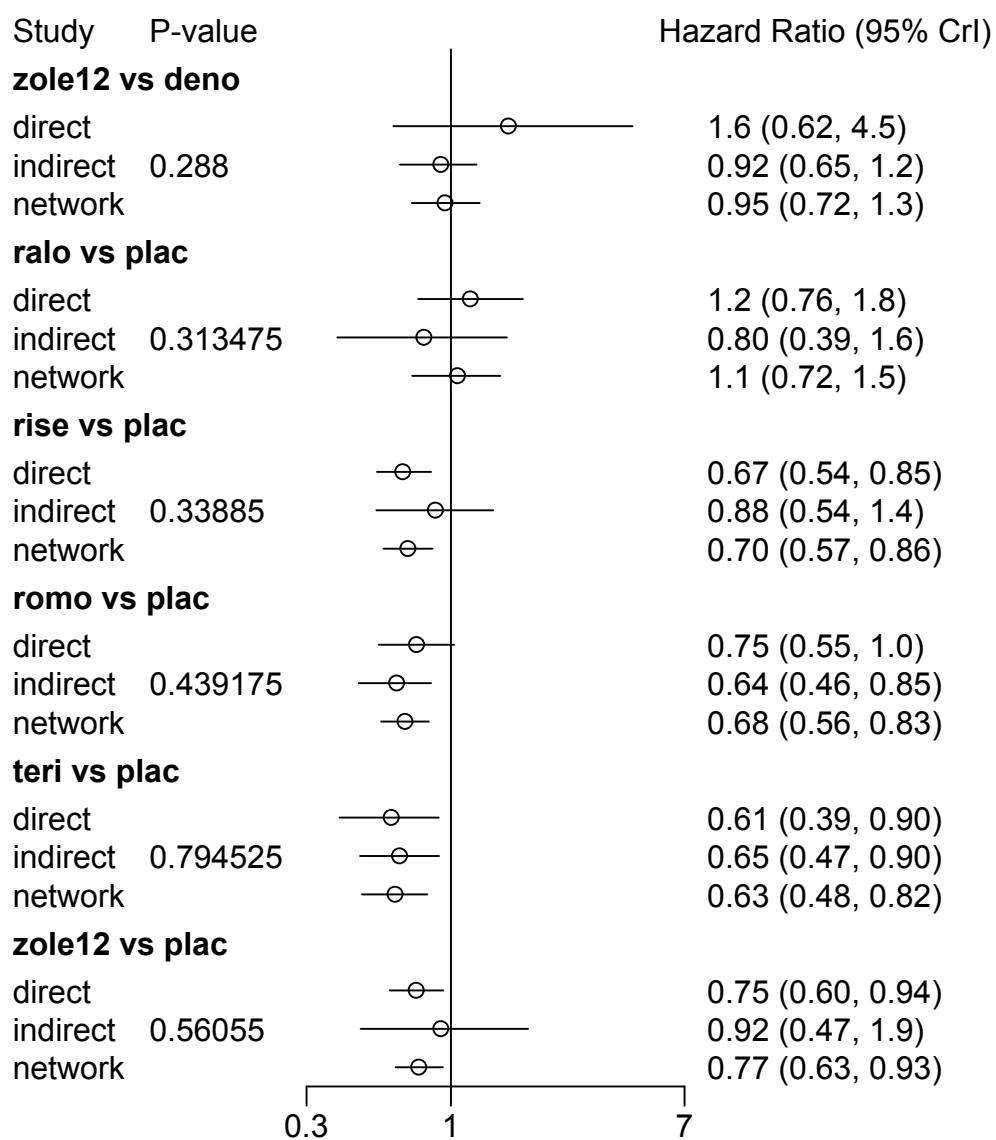

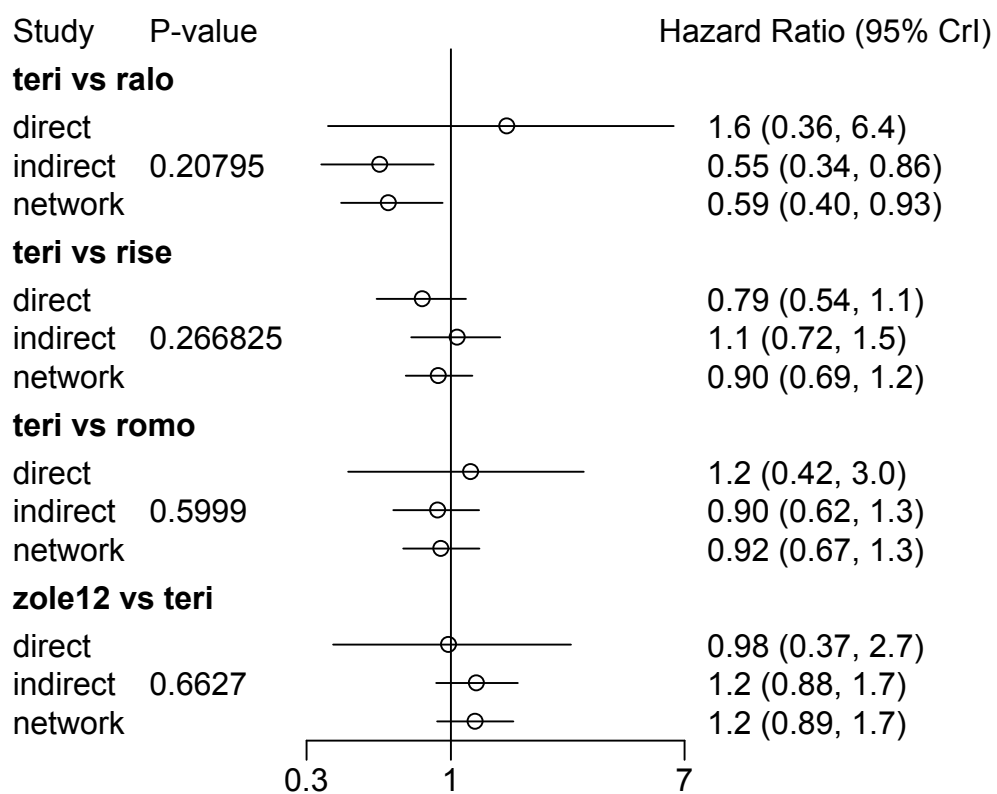

Supplement: S17 Appendix — (PDF) [file pone.0234123.s017.pdf]

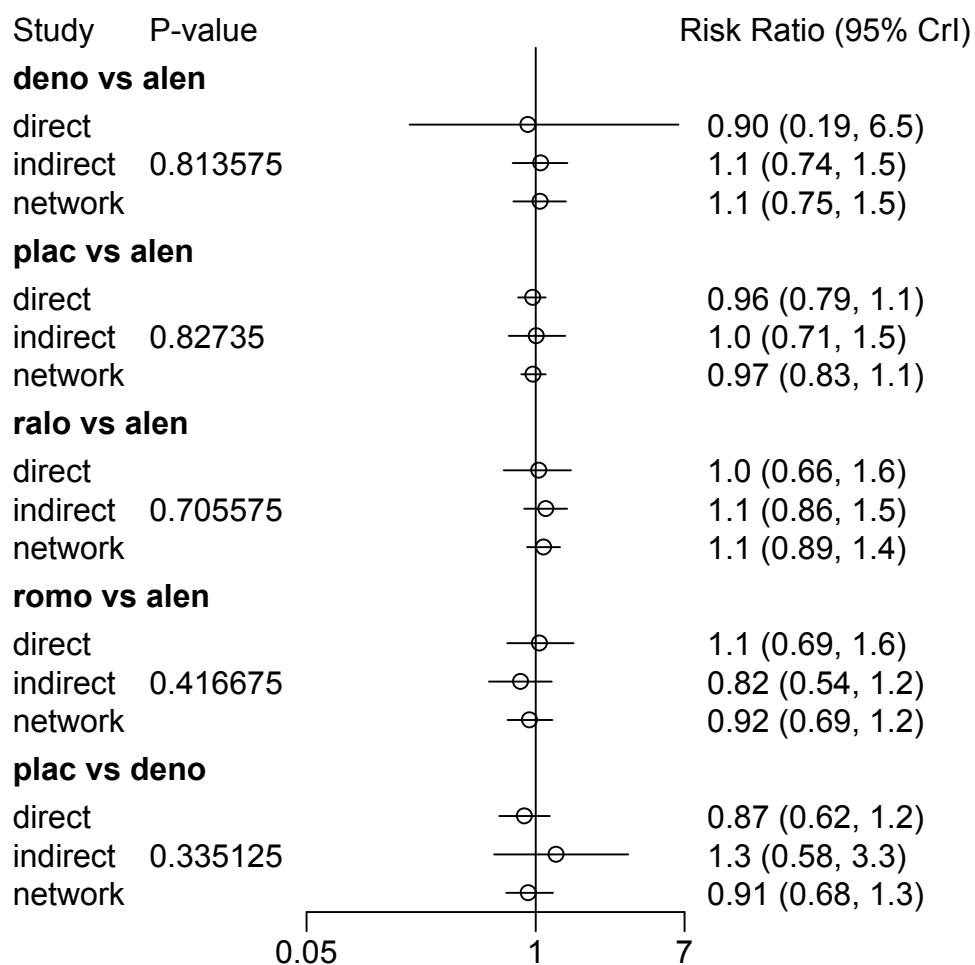

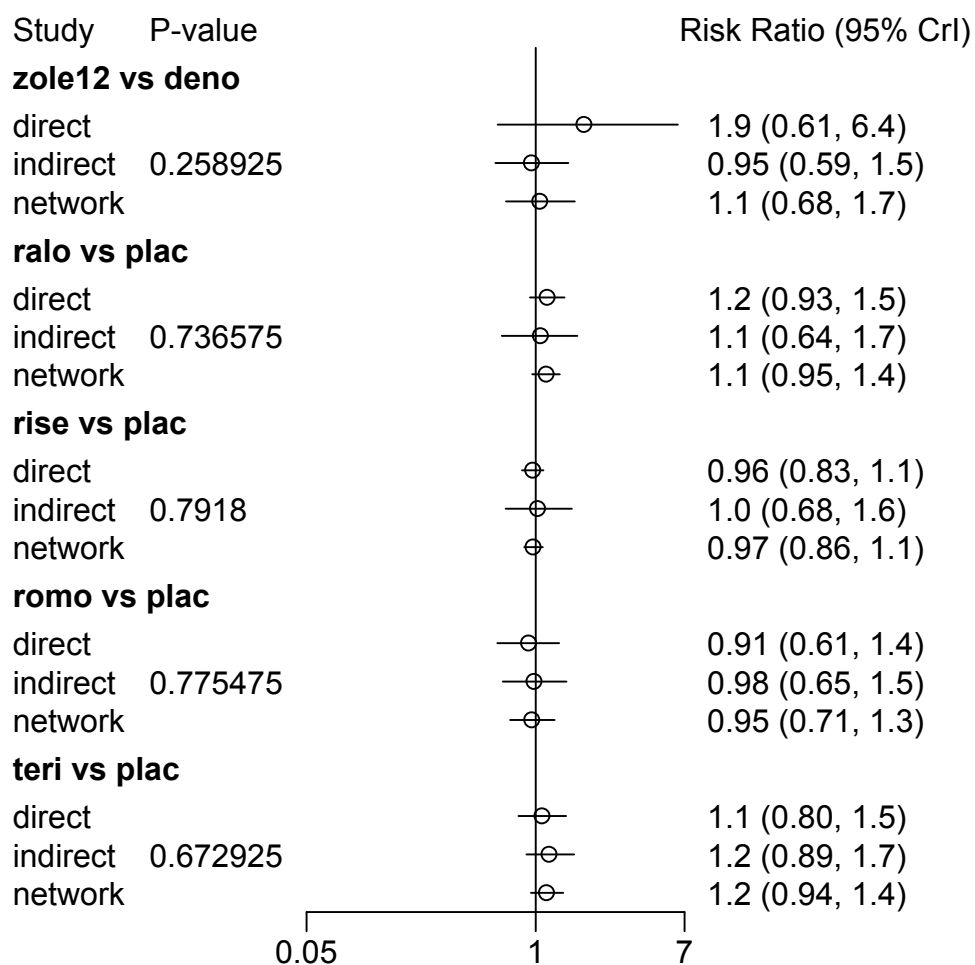

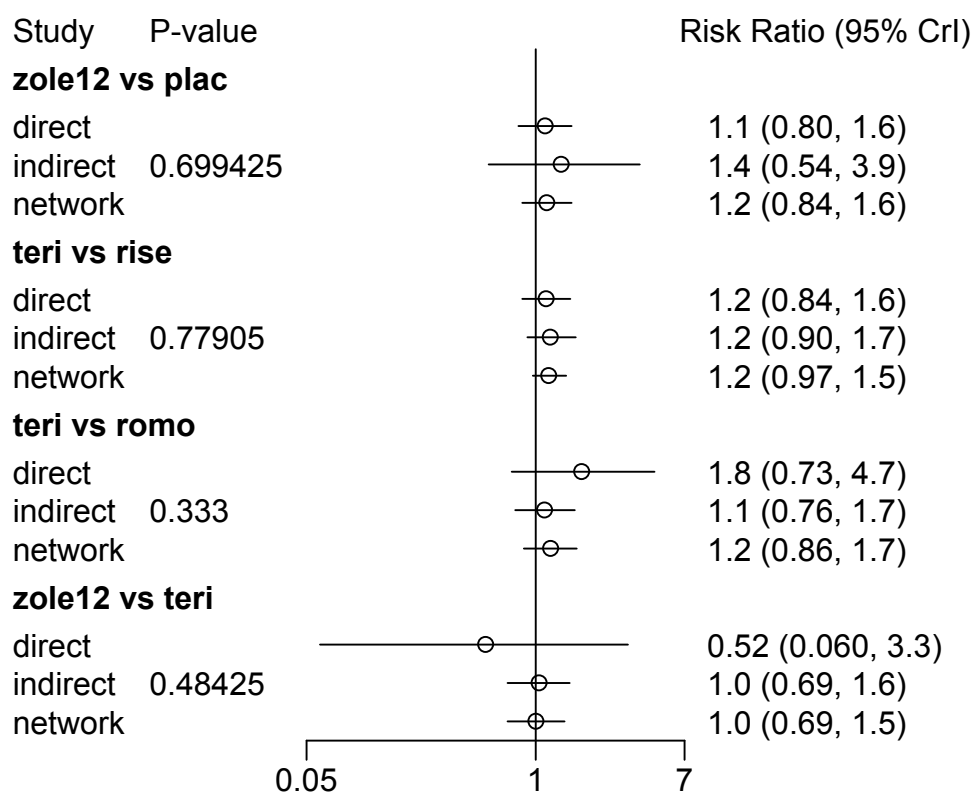

Supplement: S18 Appendix — (PDF) [file pone.0234123.s018.pdf]

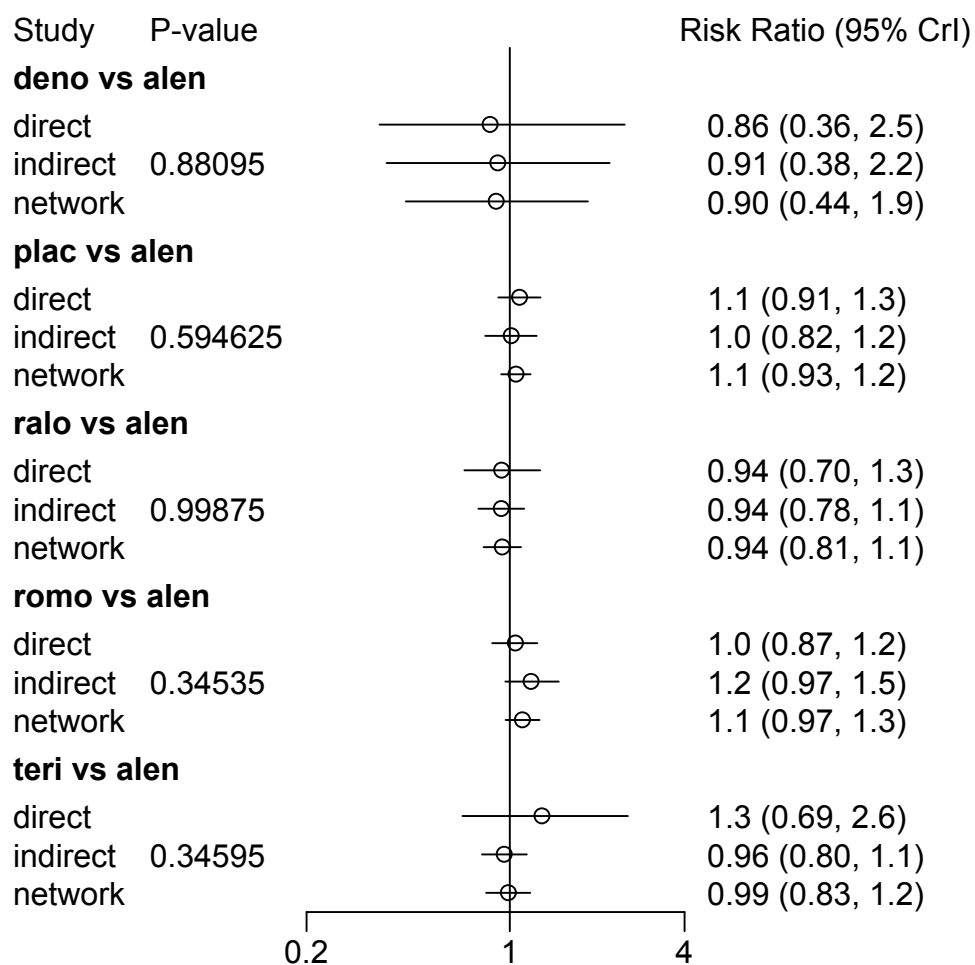

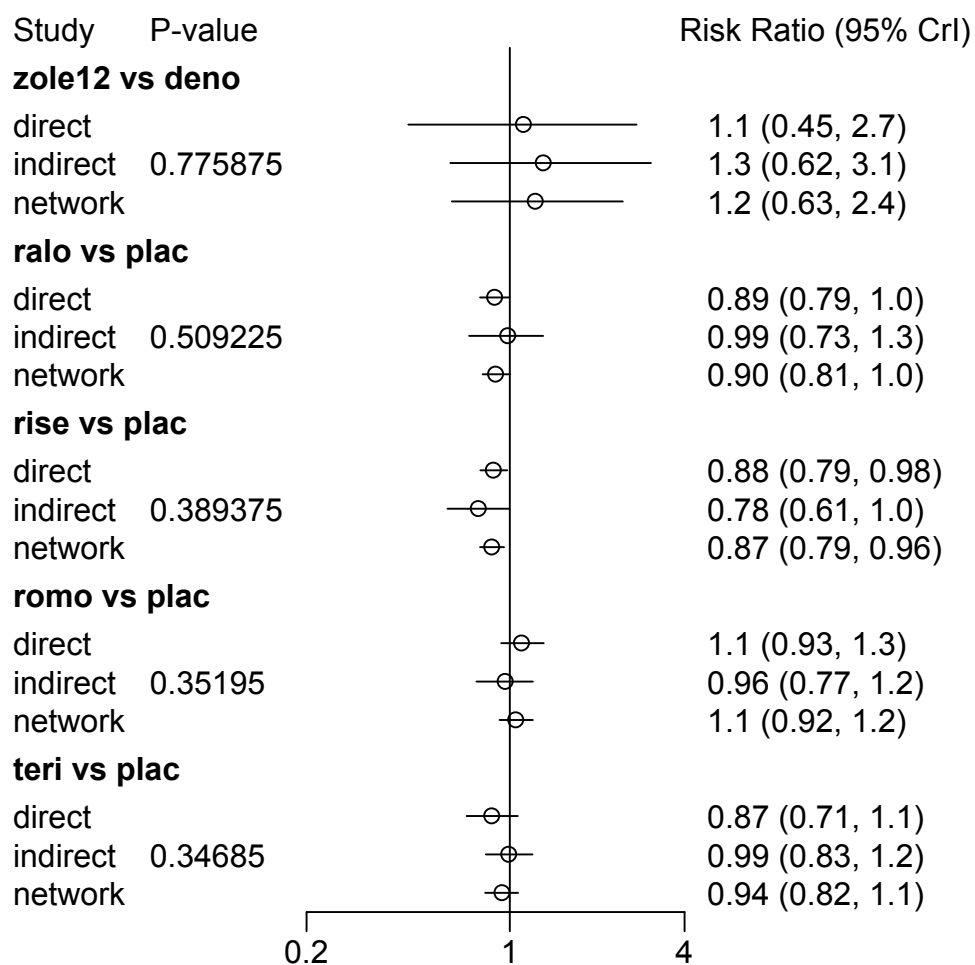

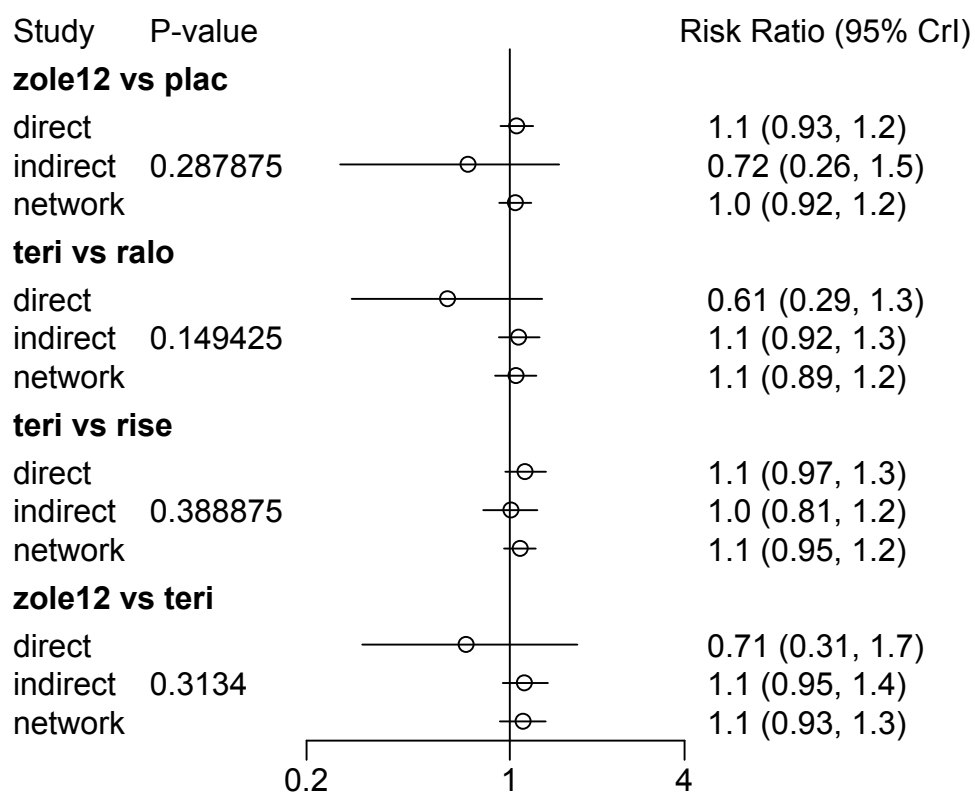

Supplement: S19 Appendix — (PDF) [file pone.0234123.s019.pdf]

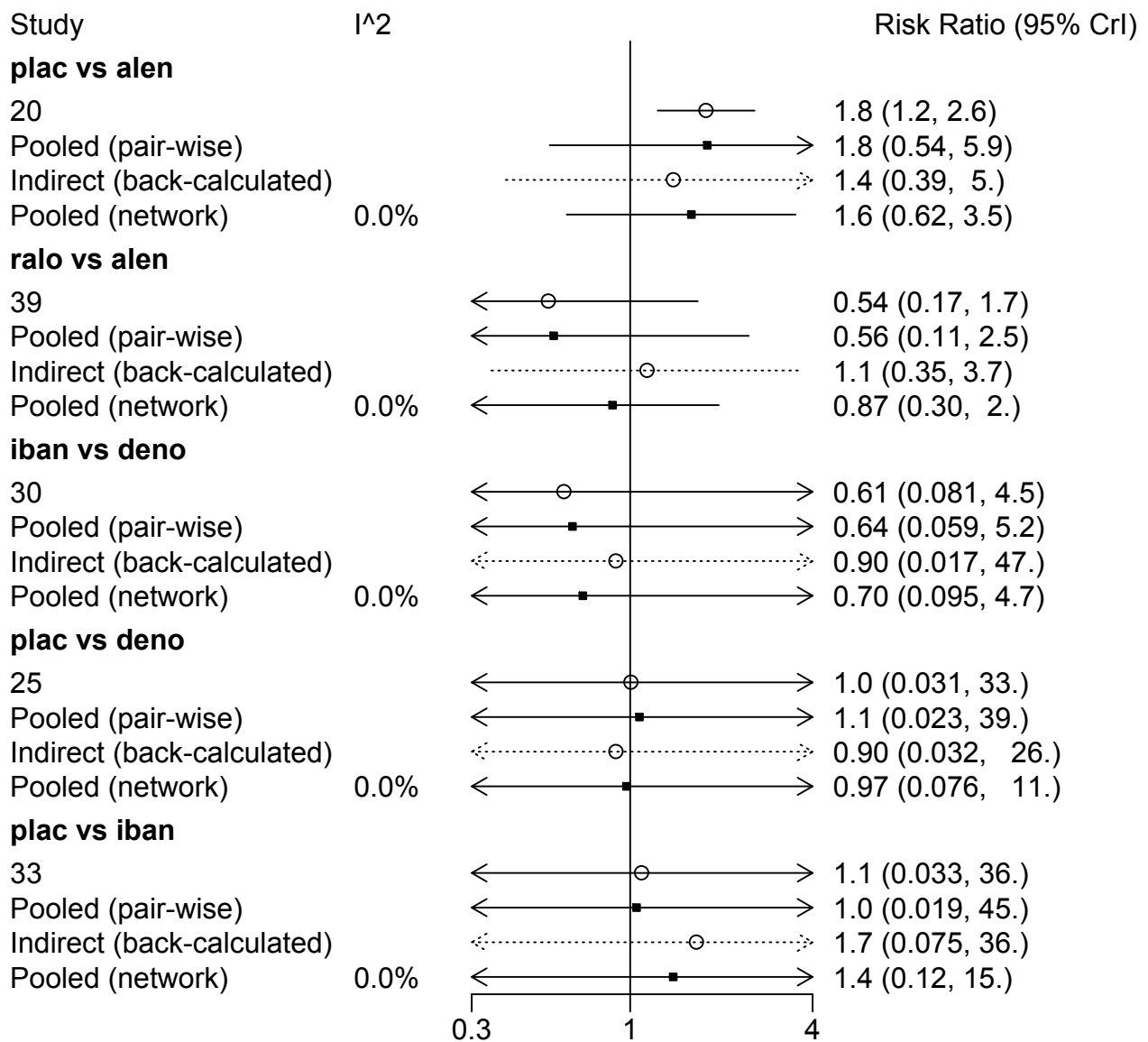

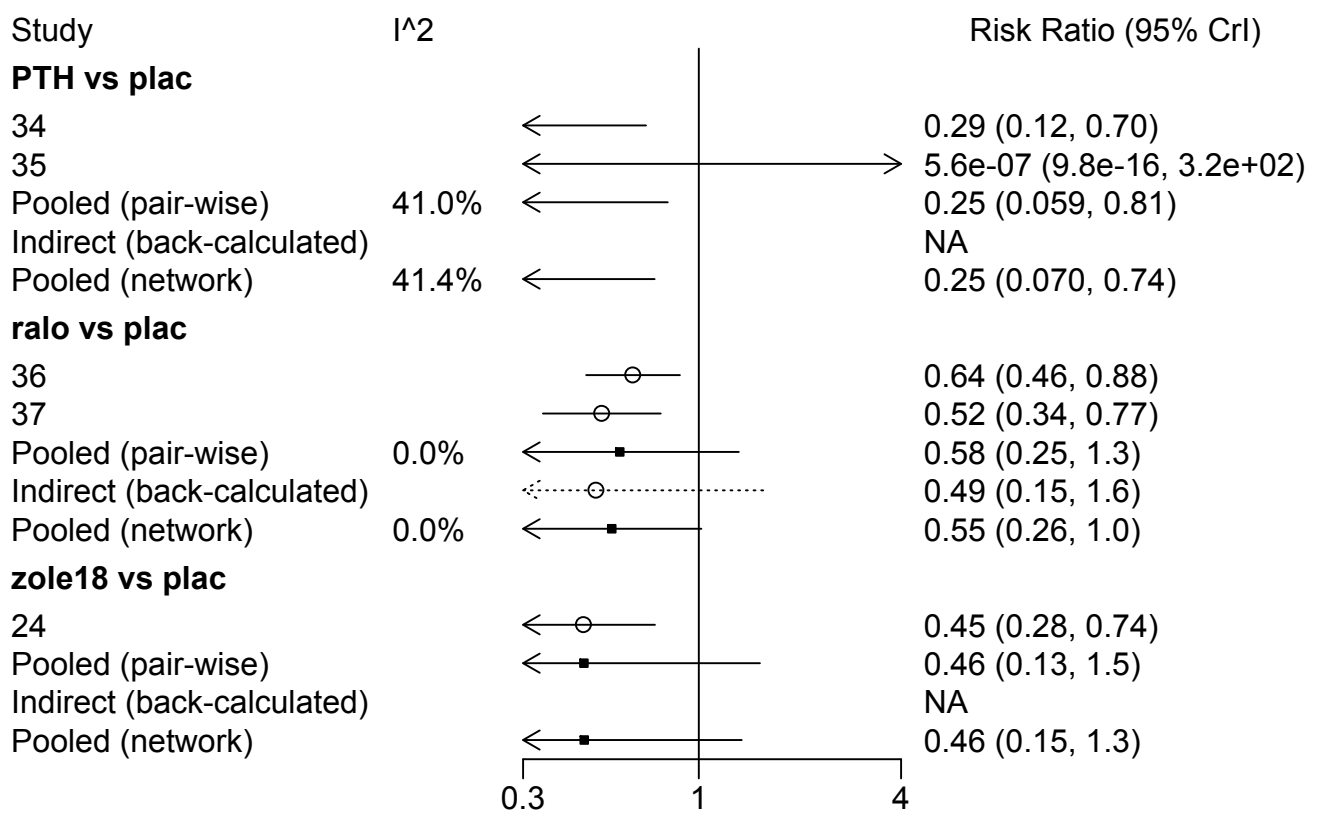

Supplement: S20 Appendix — (PDF) [file pone.0234123.s020.pdf]

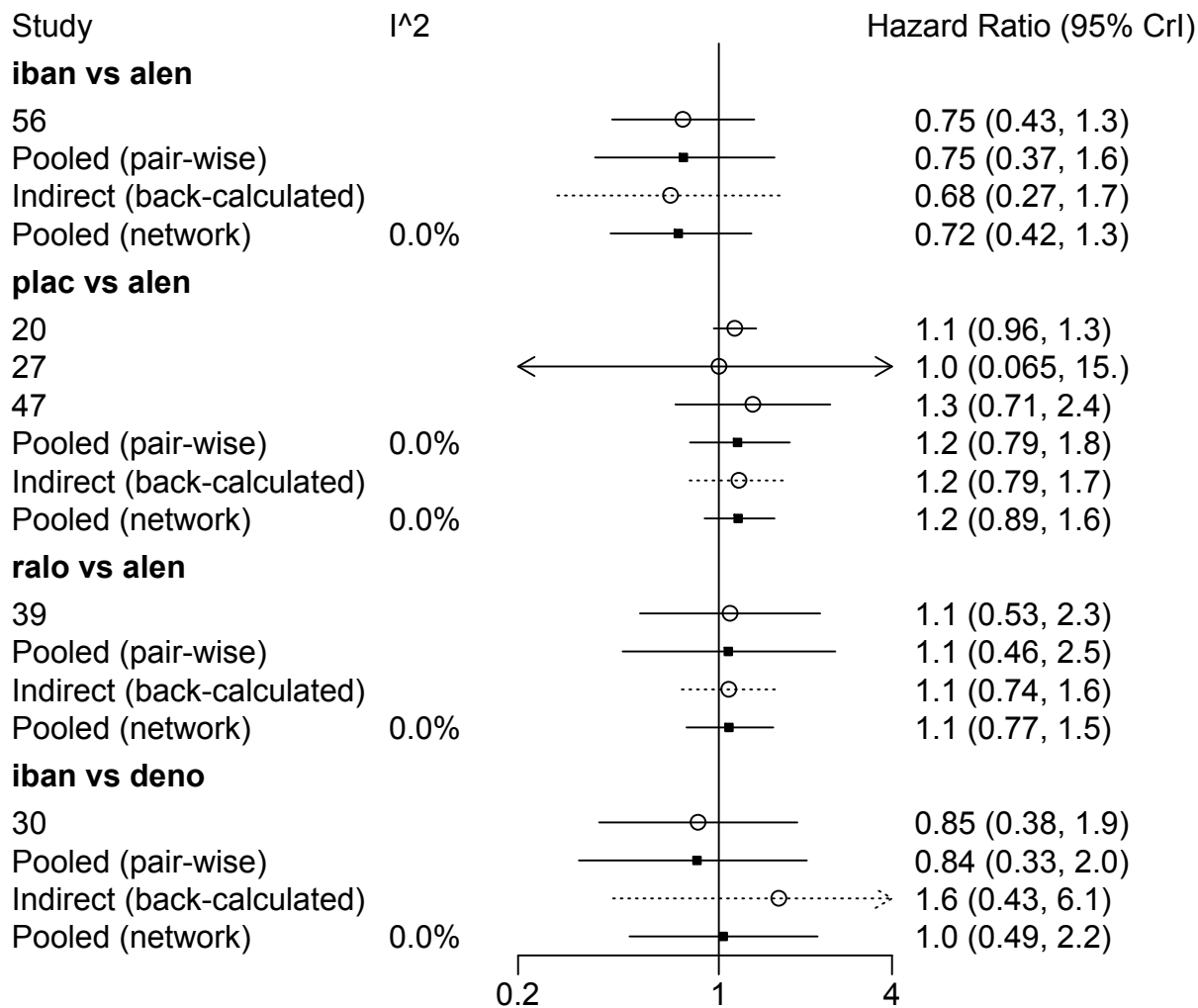

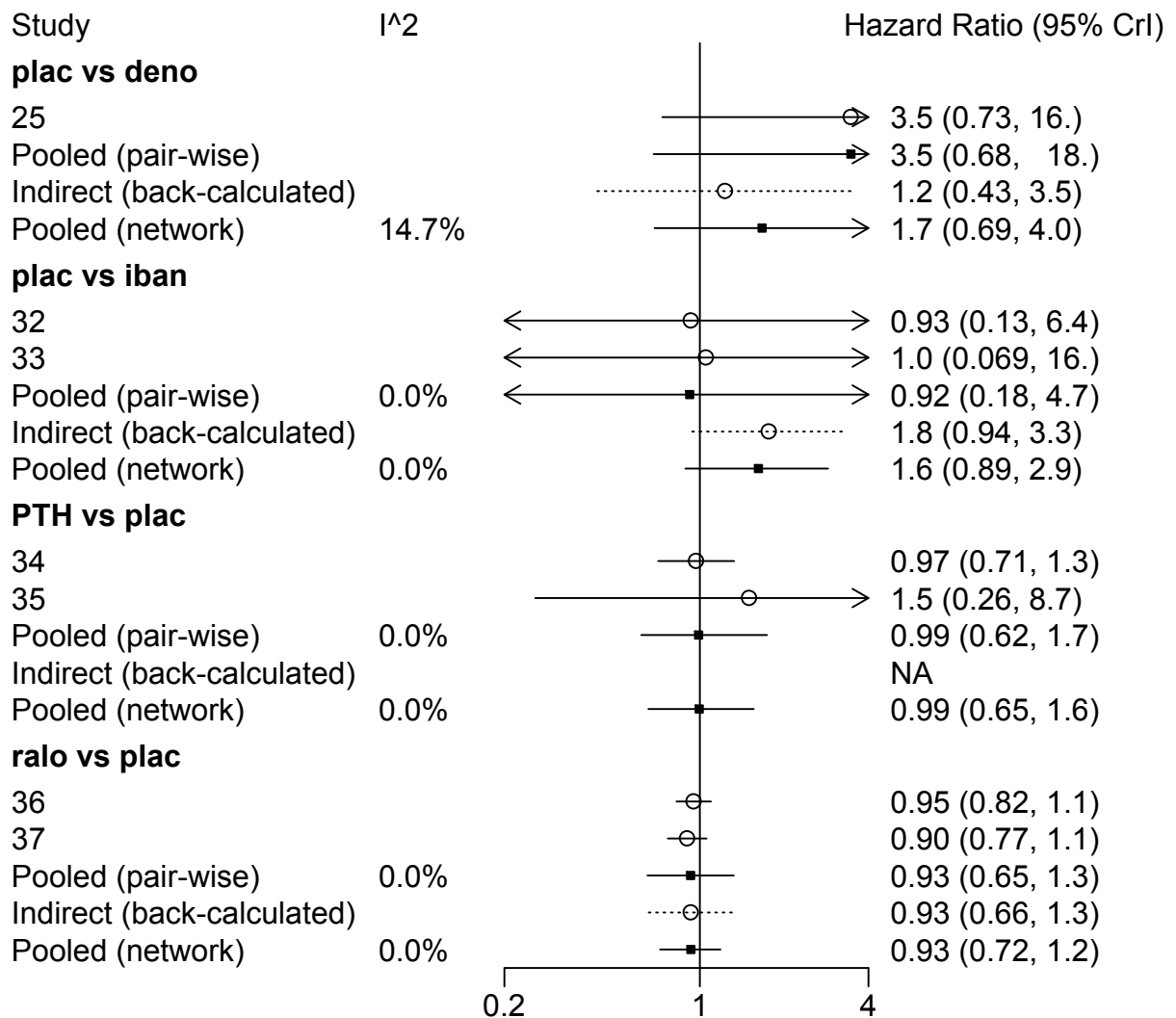

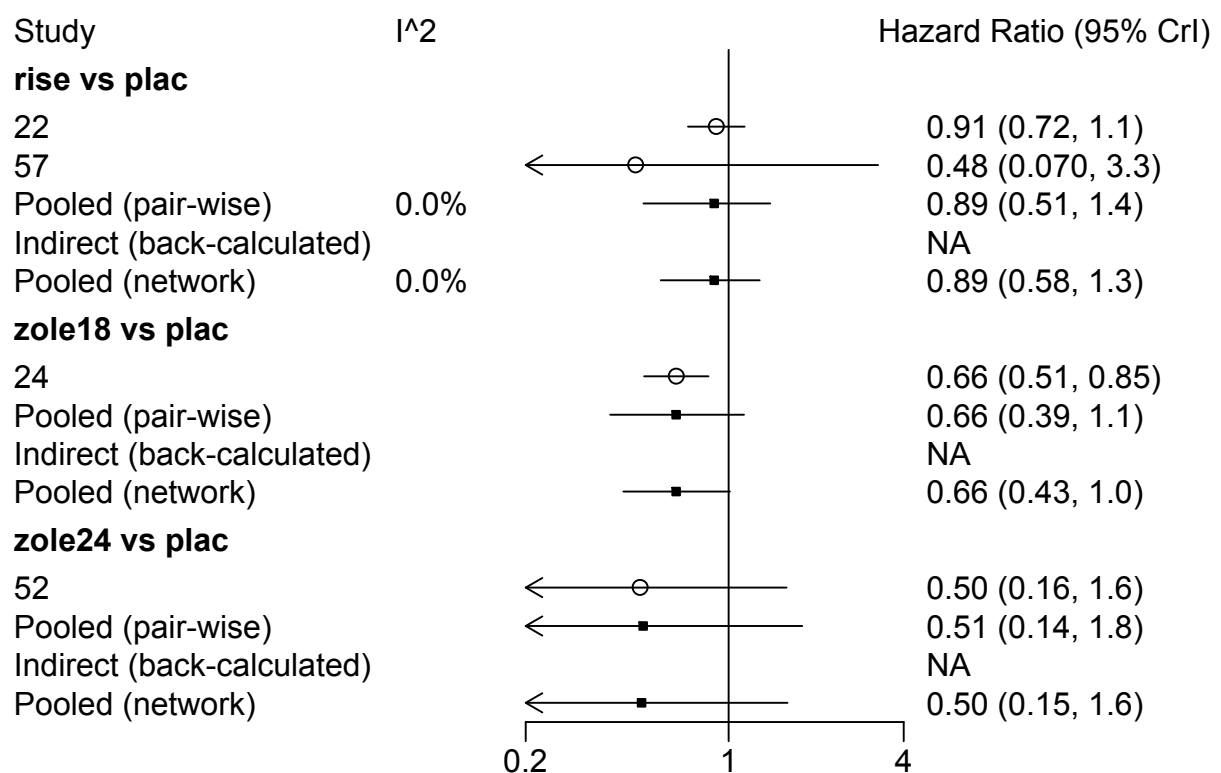

Supplement: S21 Appendix — (PDF) [file pone.0234123.s021.pdf]

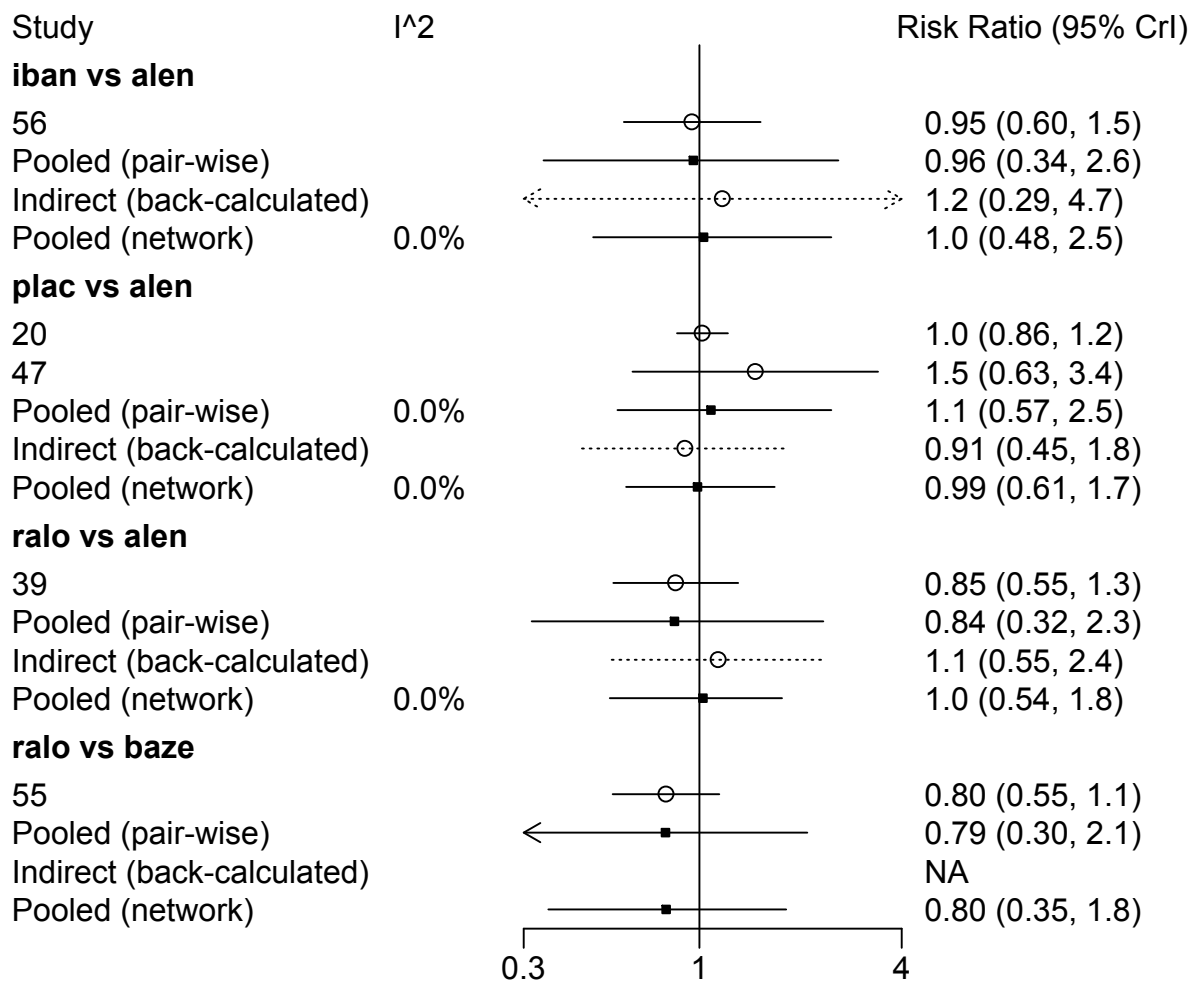

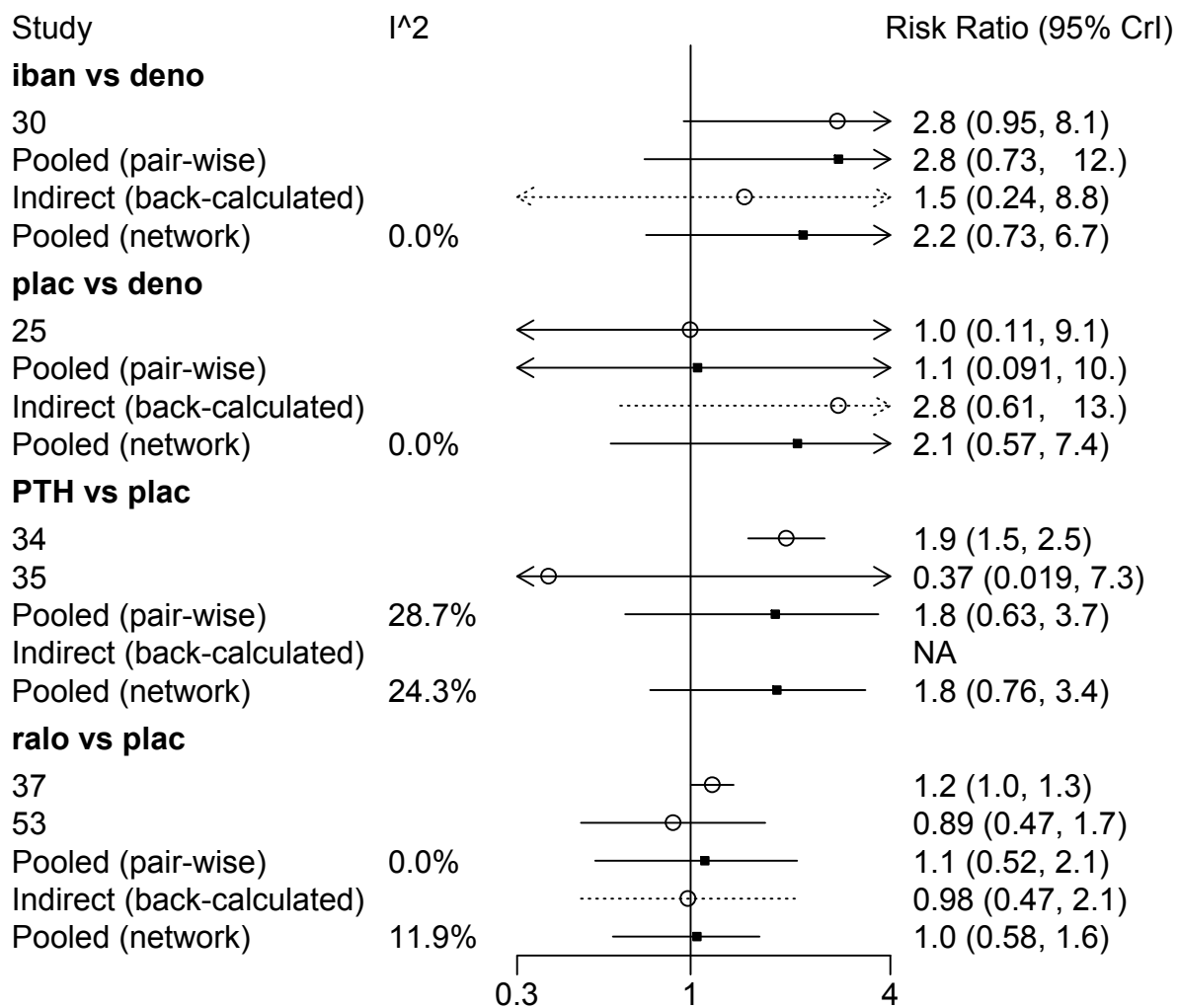

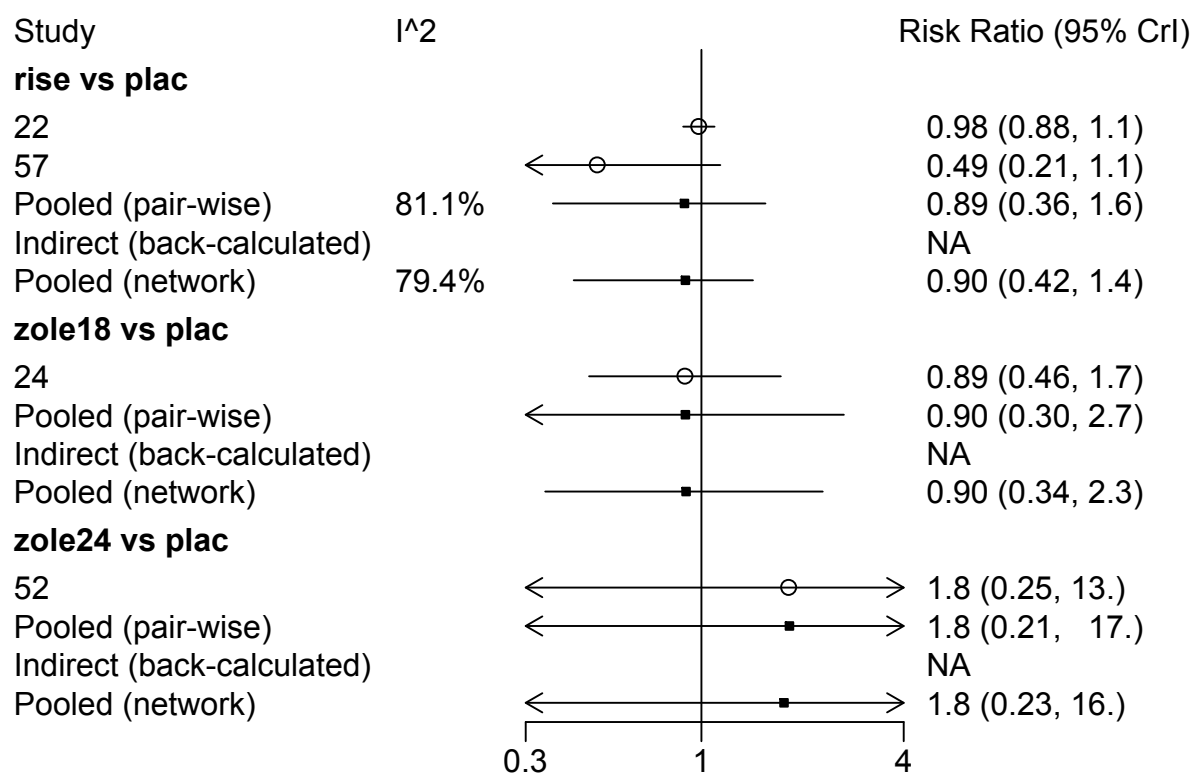

Supplement: S22 Appendix — (PDF) [file pone.0234123.s022.pdf]

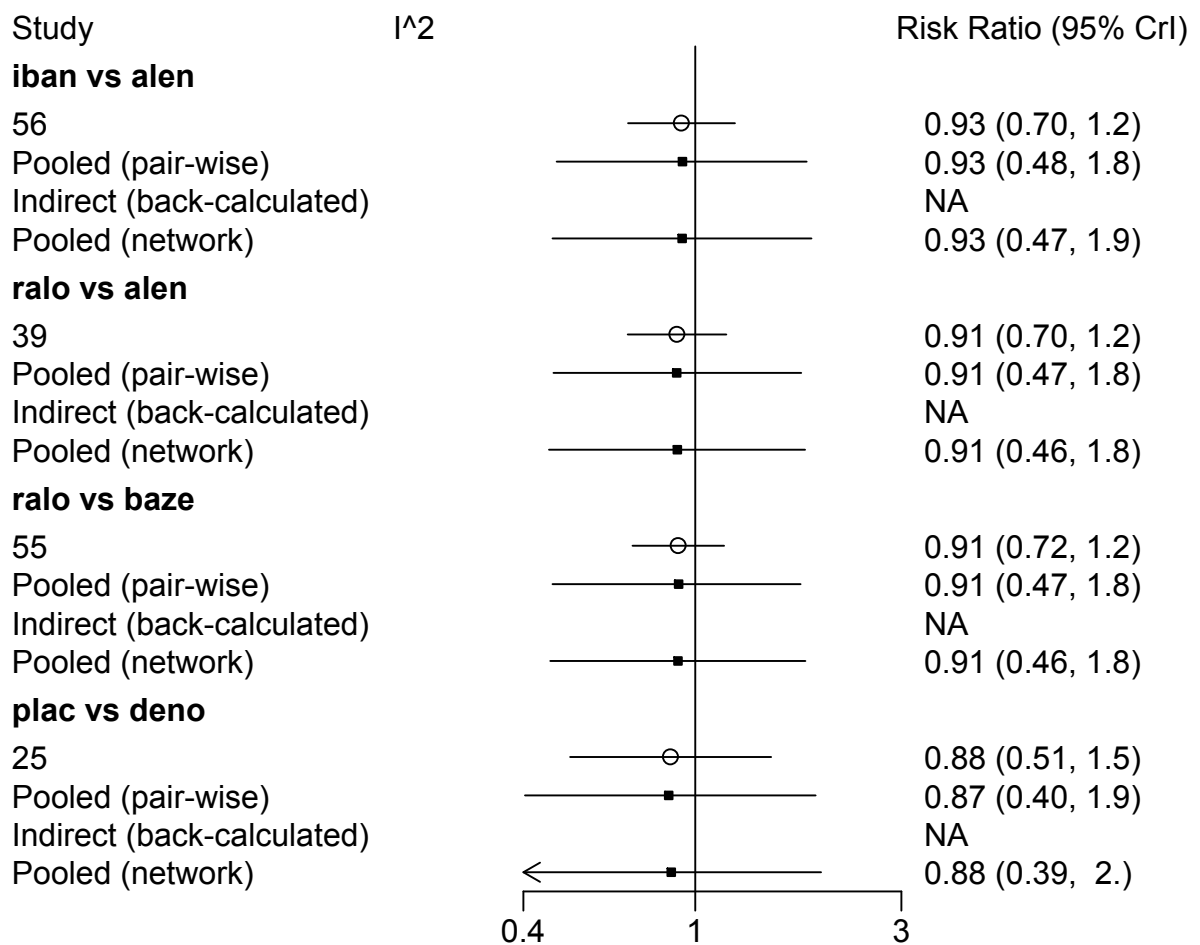

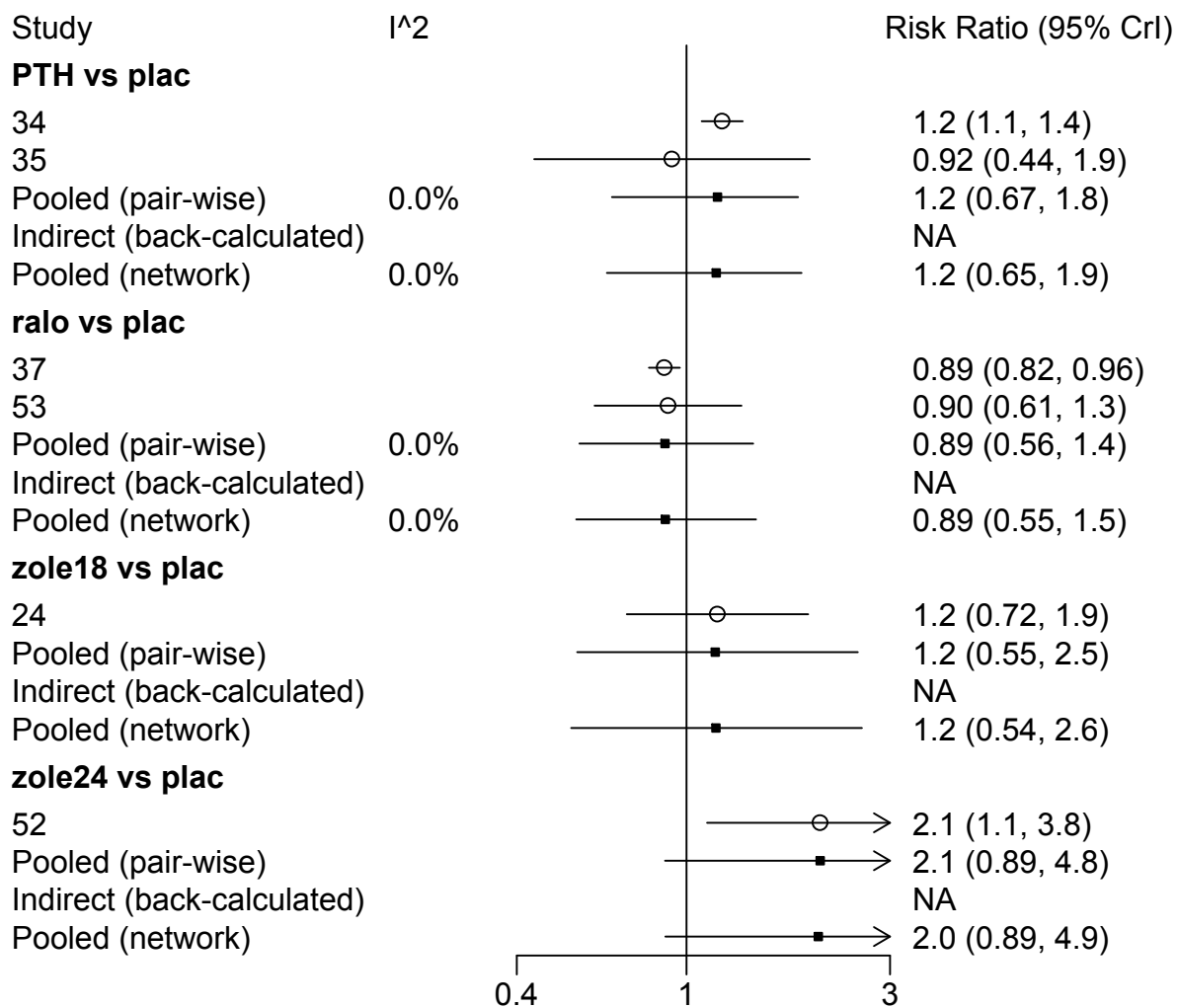

Supplement: S23 Appendix — (PDF) [file pone.0234123.s023.pdf]

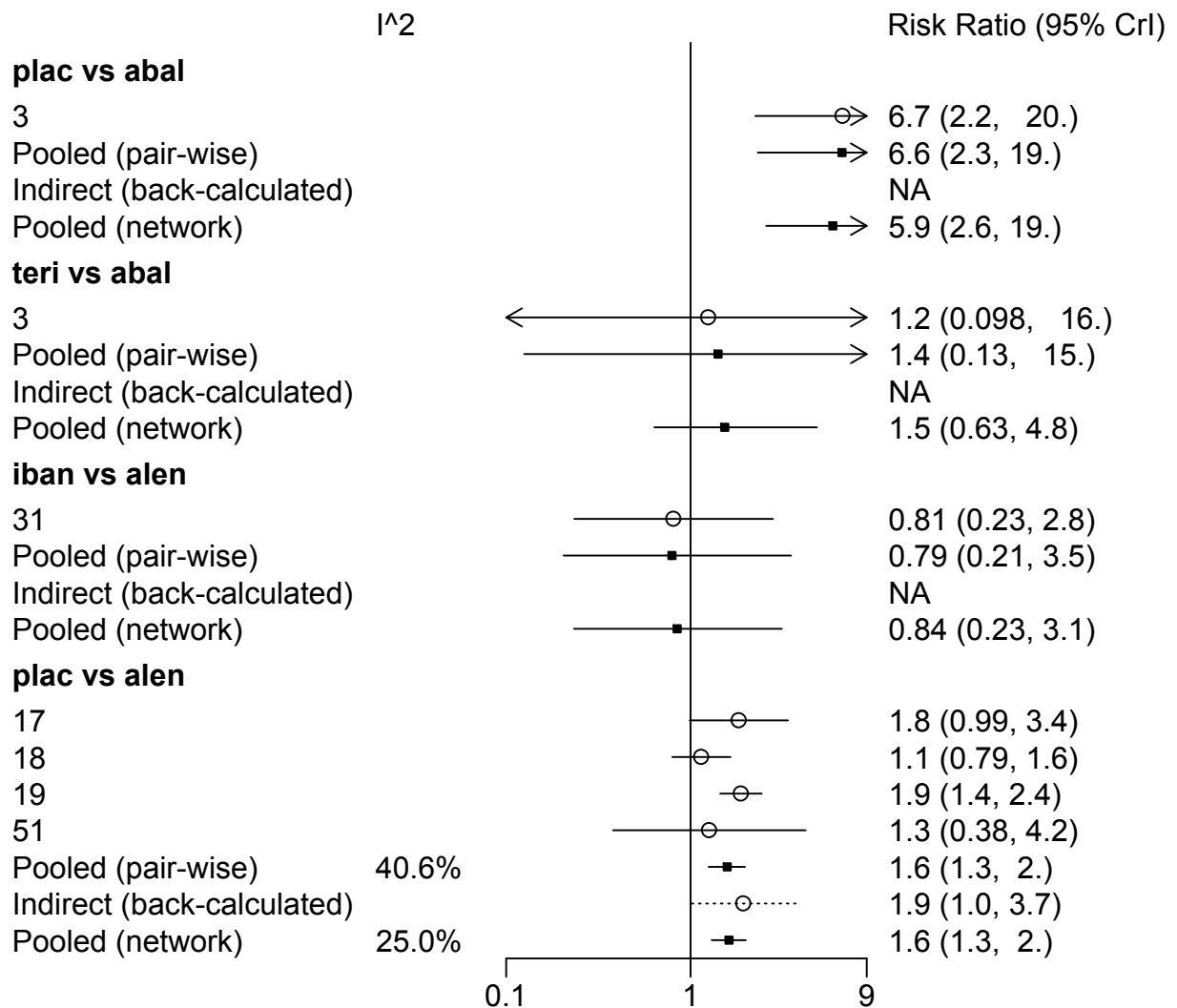

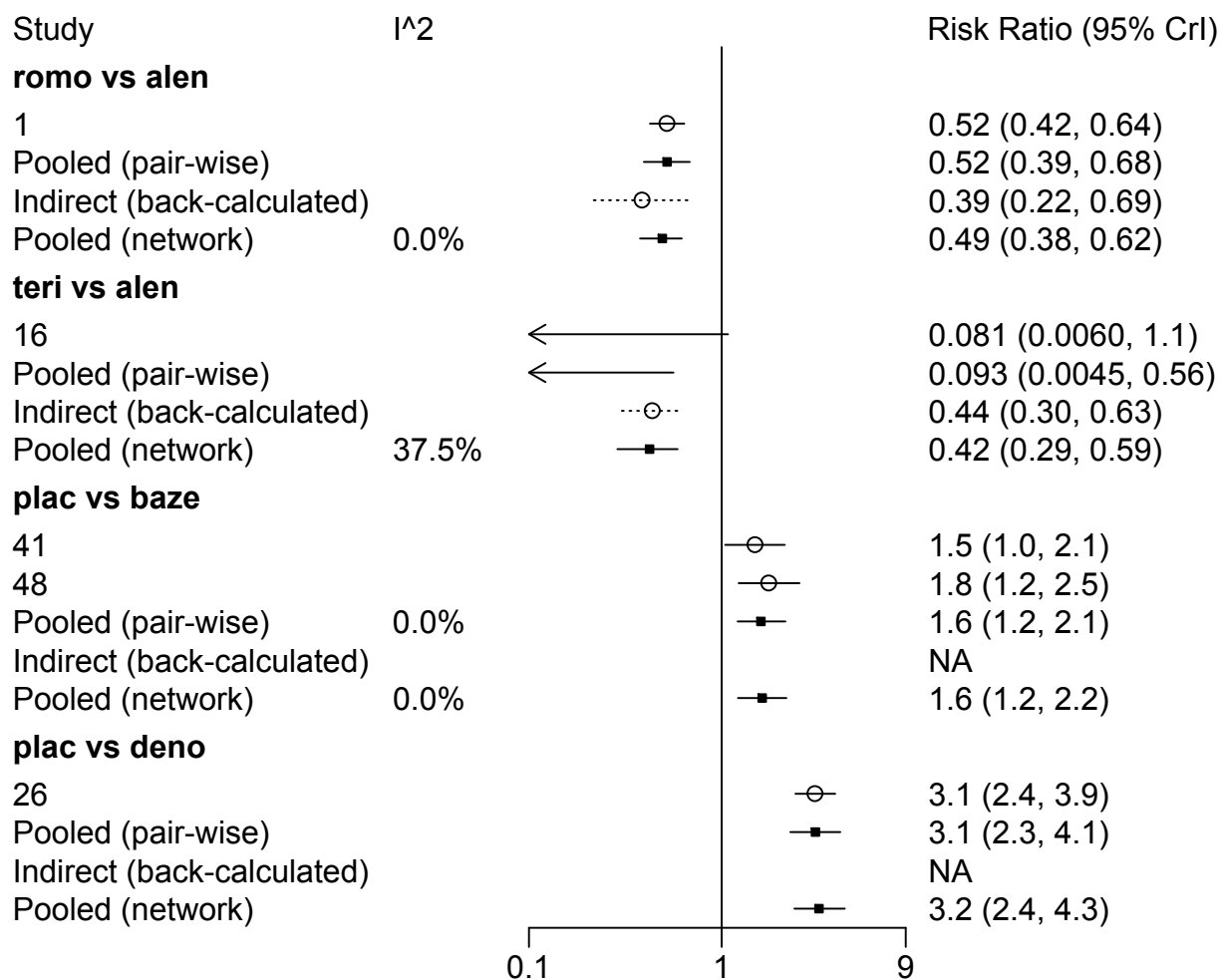

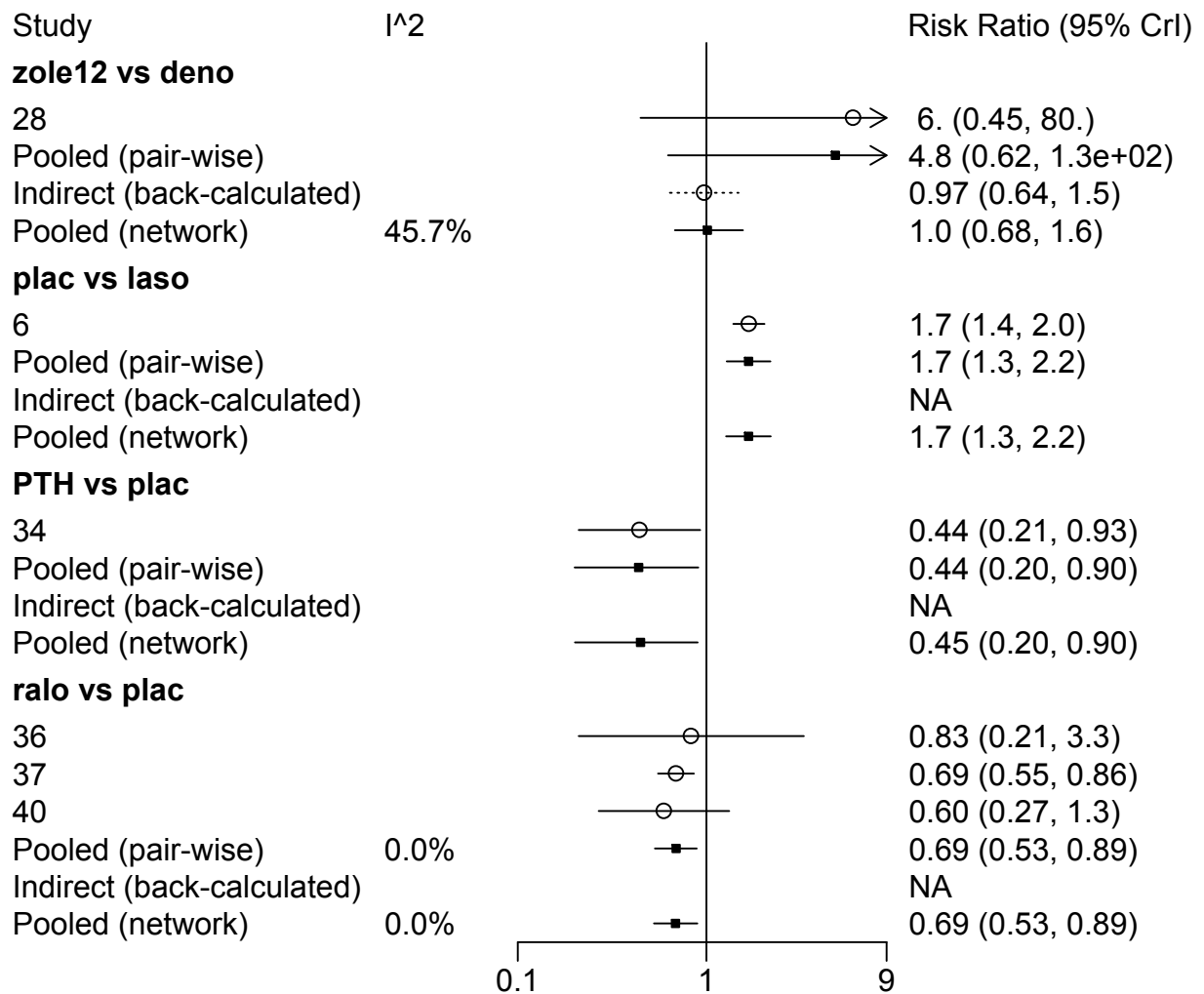

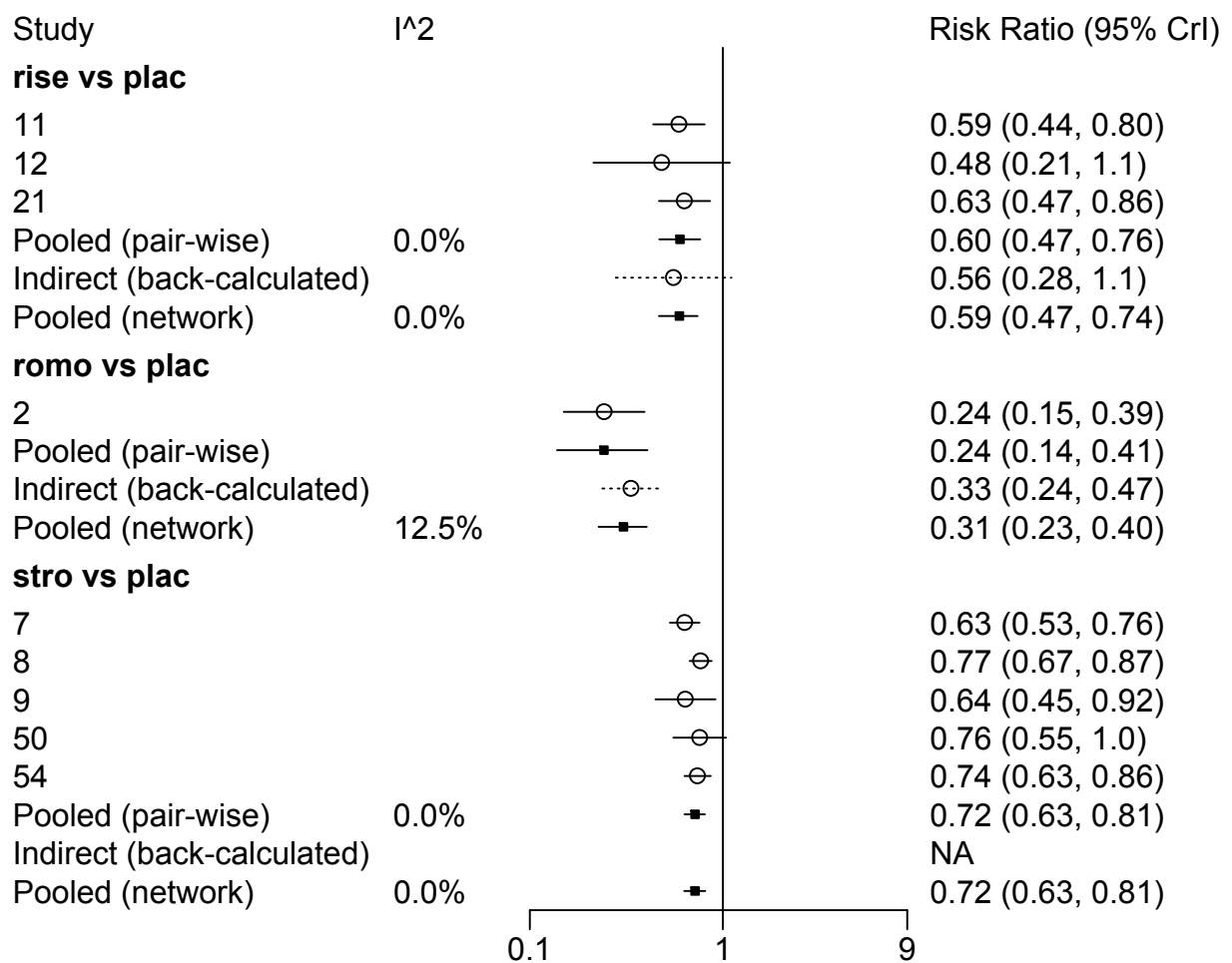

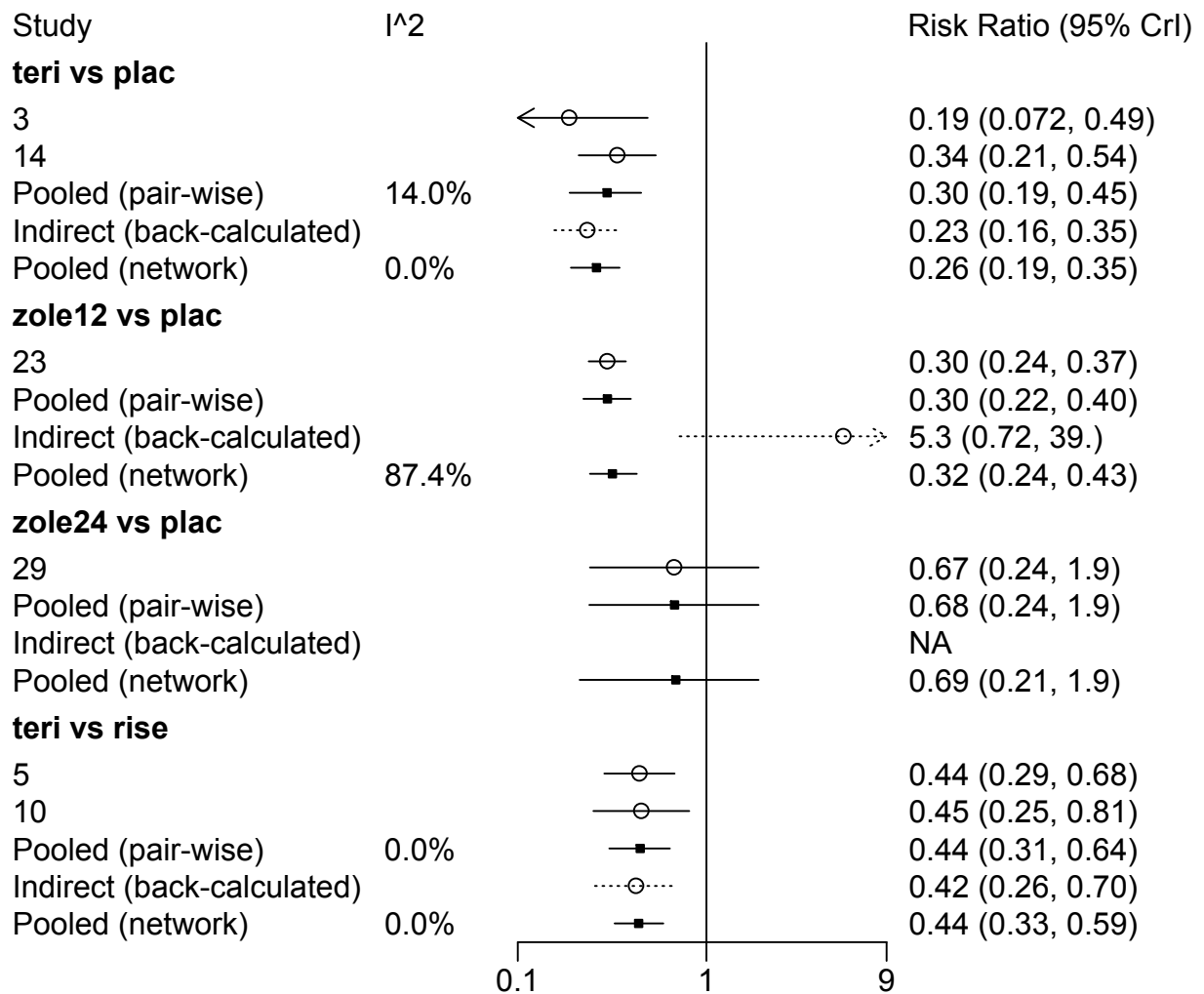

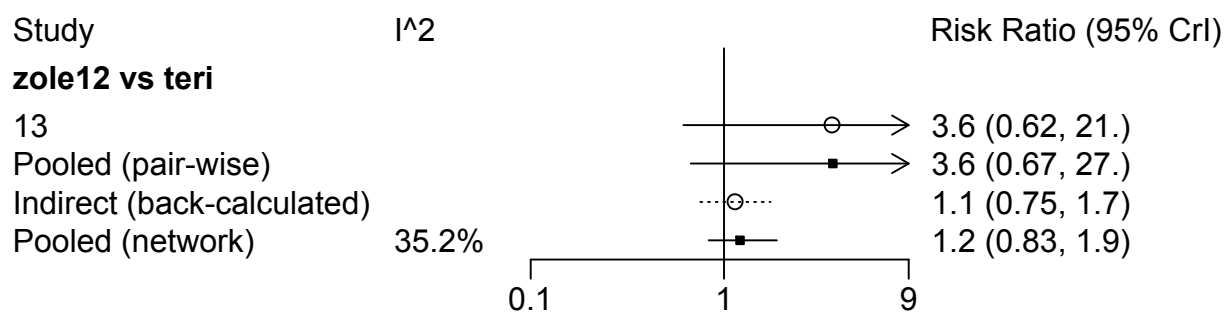

Supplement: S24 Appendix — (PDF) [file pone.0234123.s024.pdf]

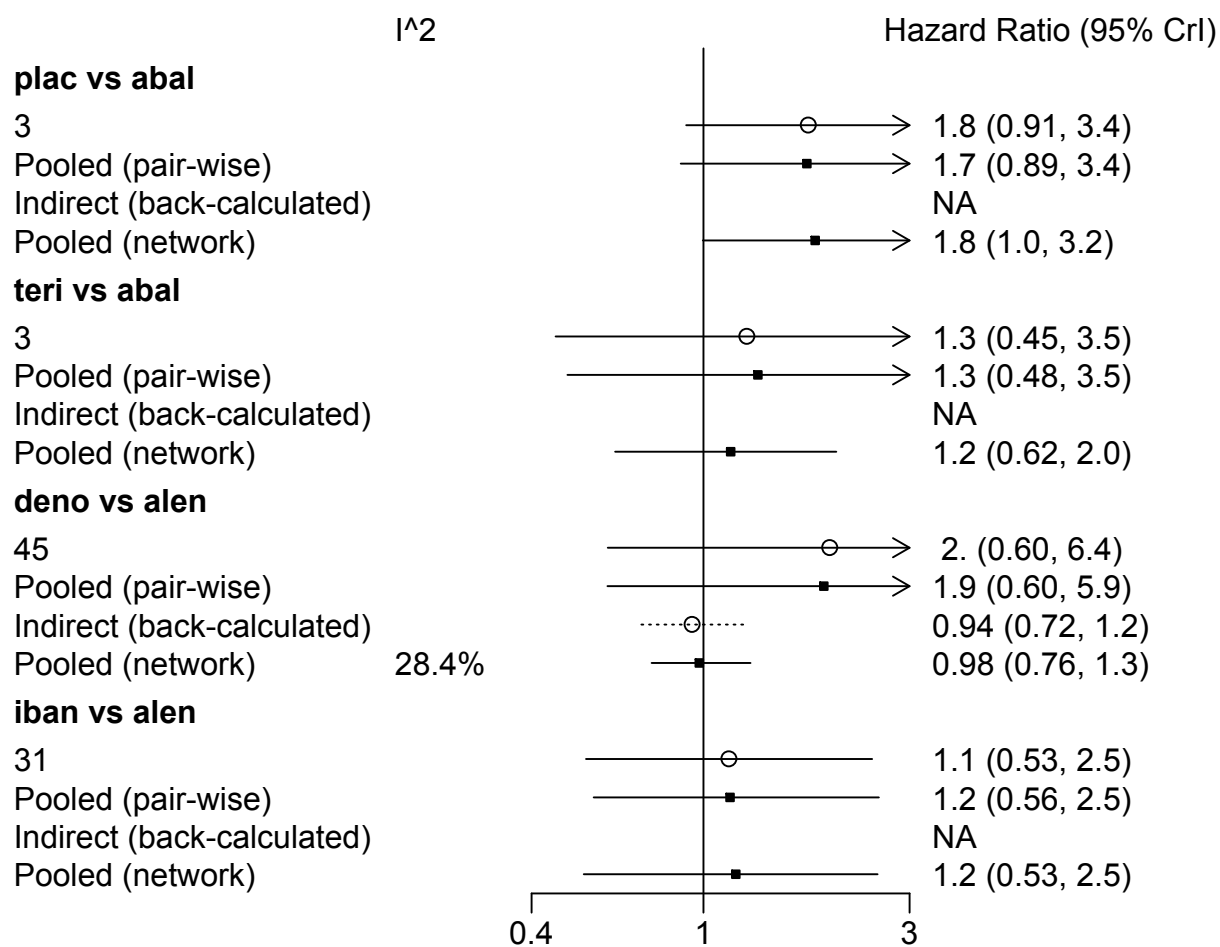

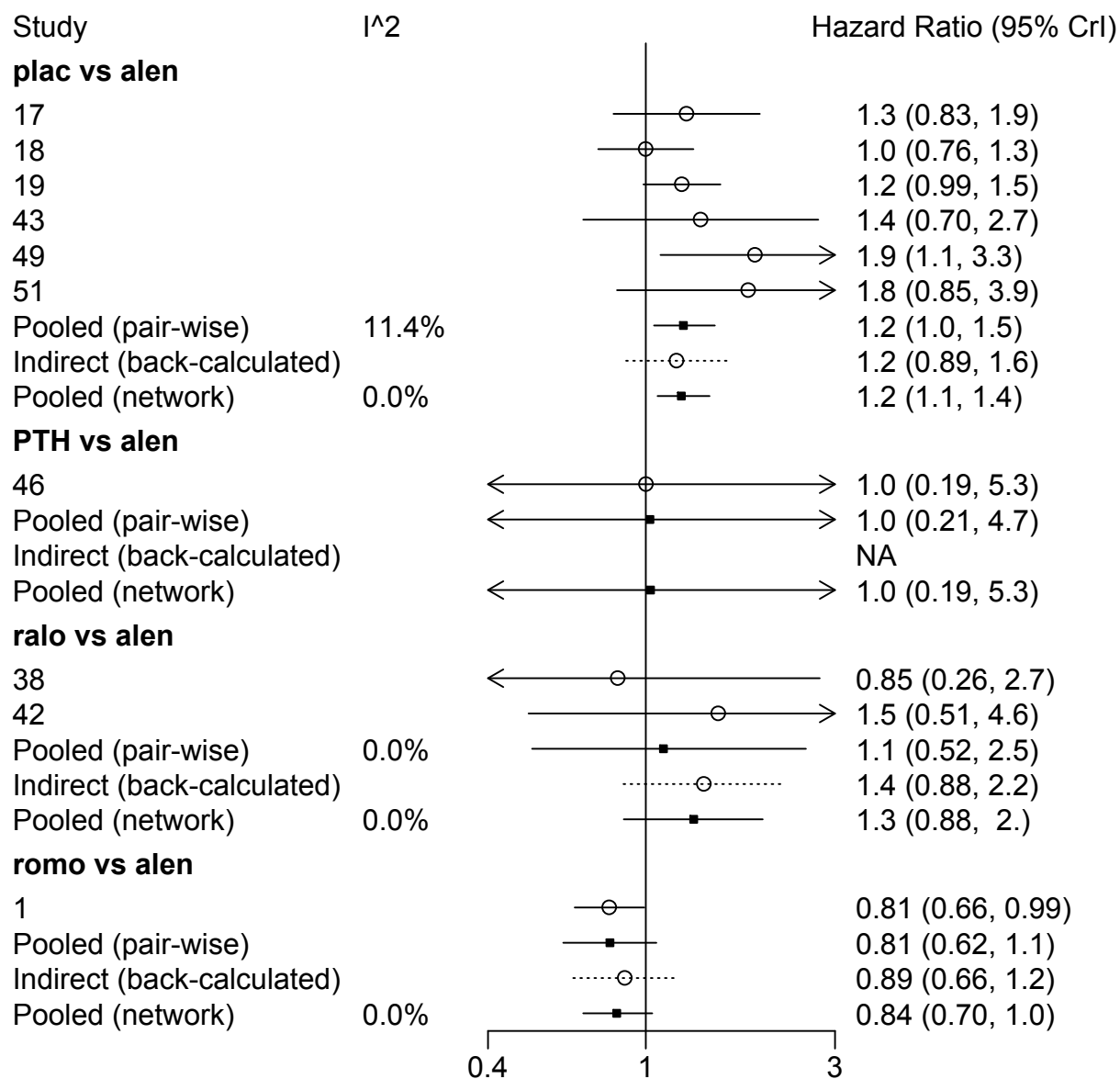

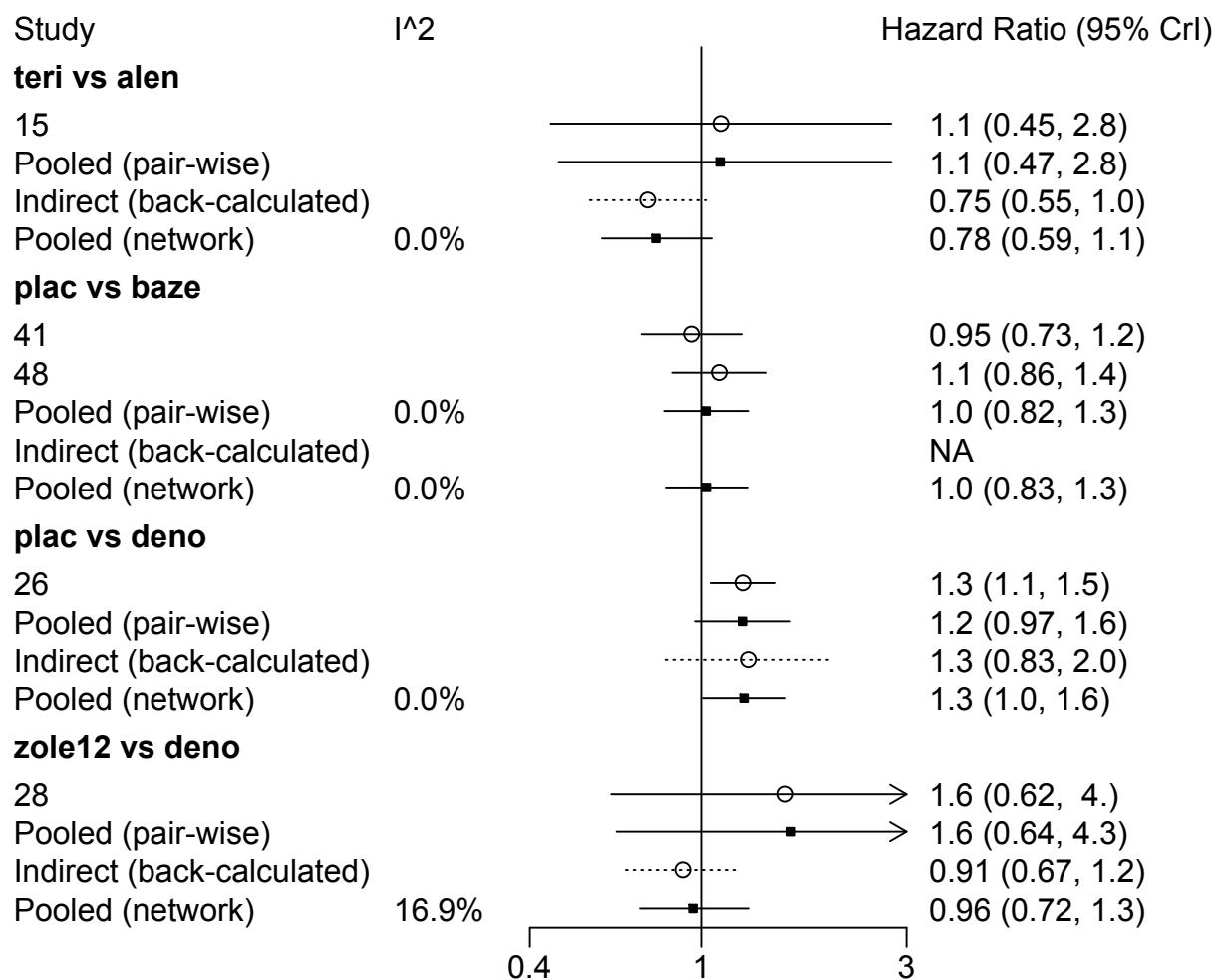

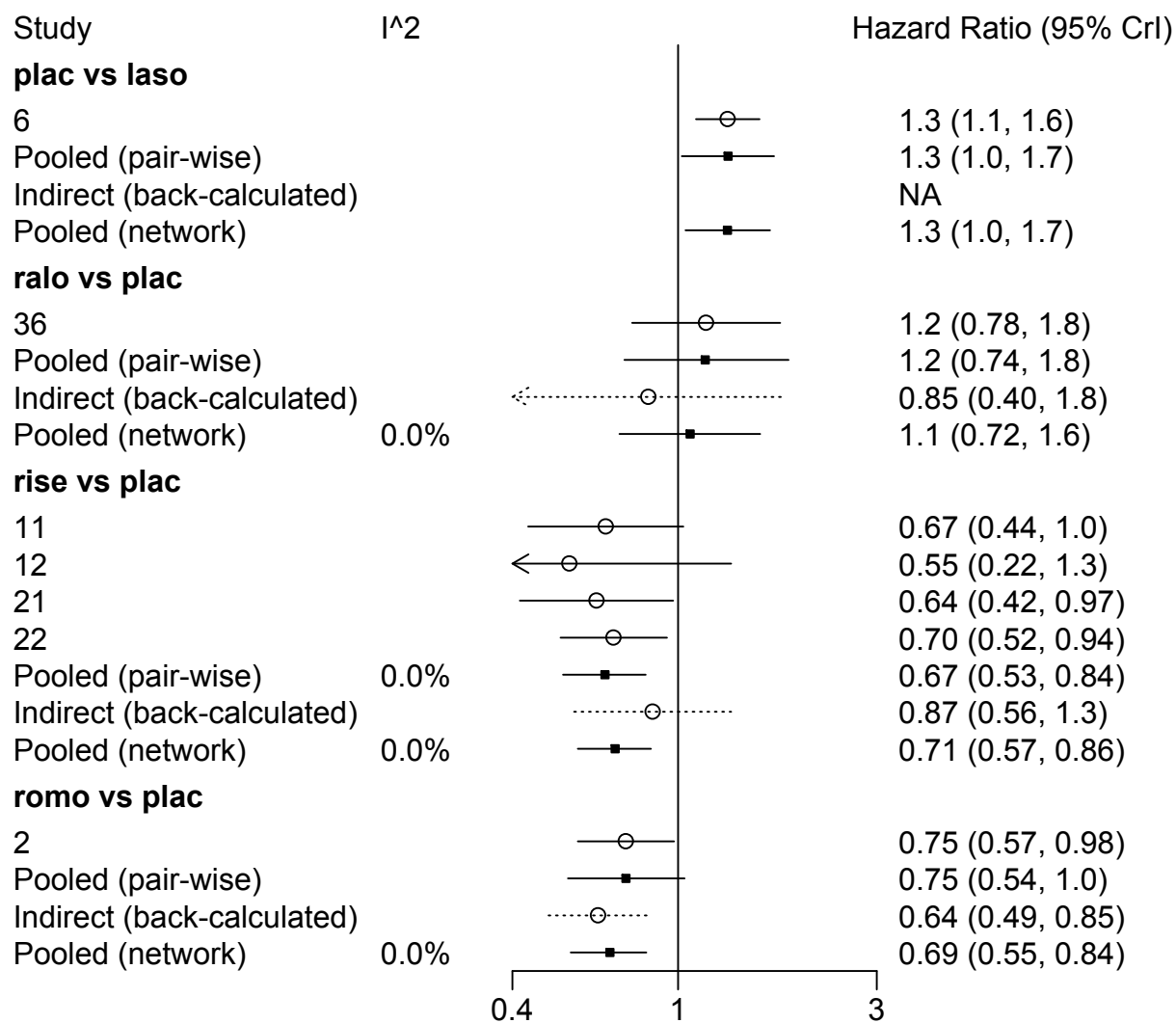

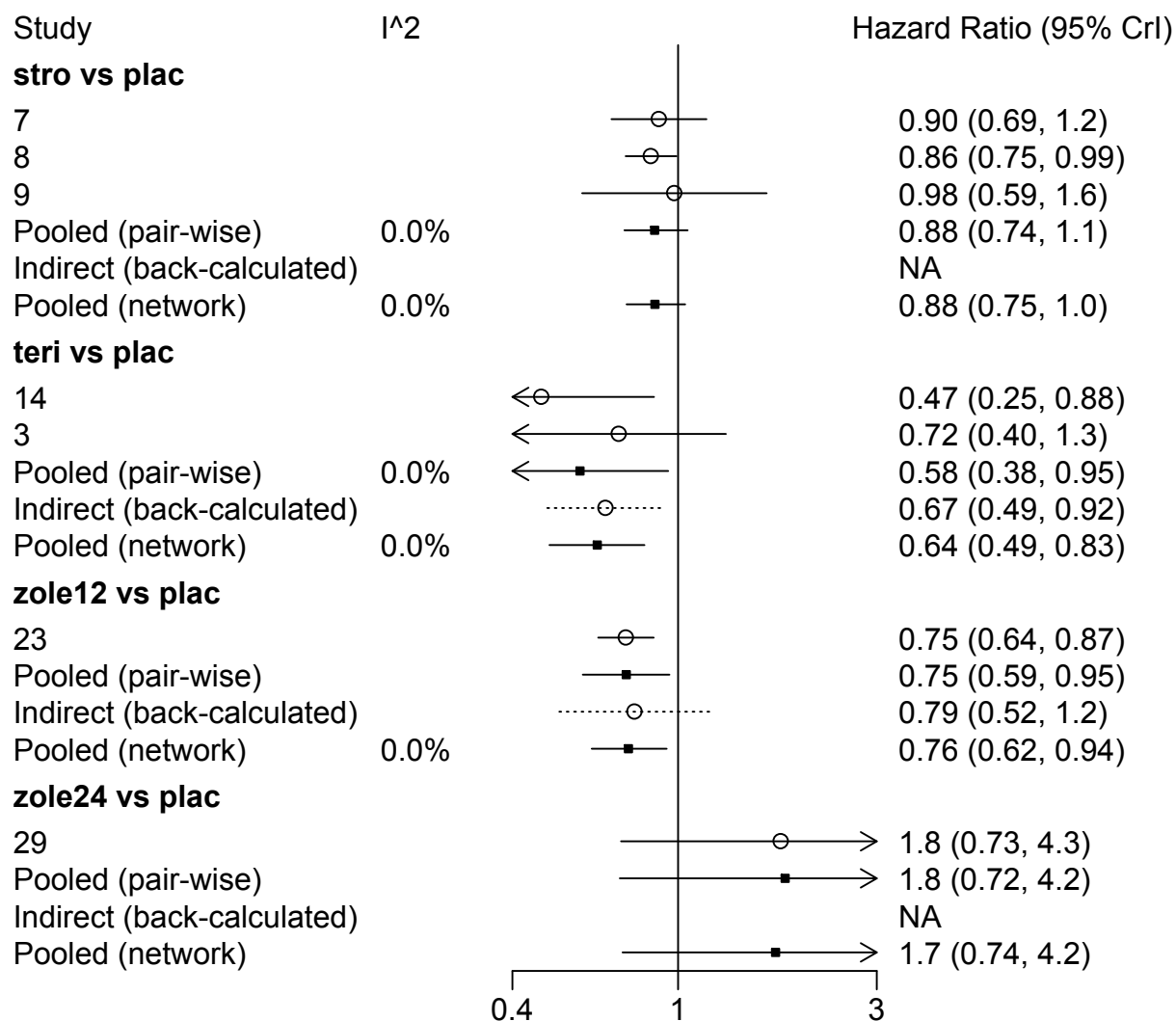

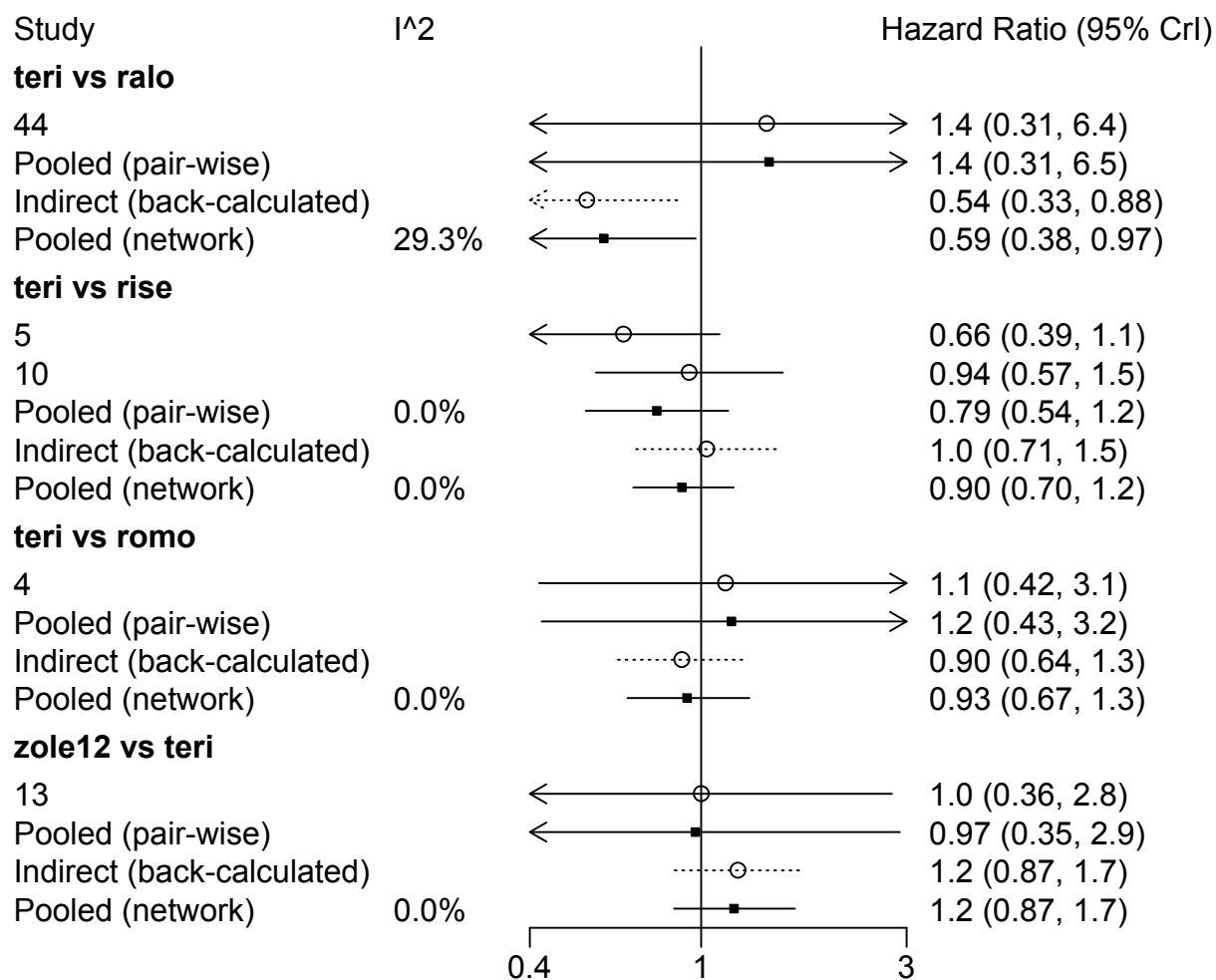

Supplement: S25 Appendix — (PDF) [file pone.0234123.s025.pdf]

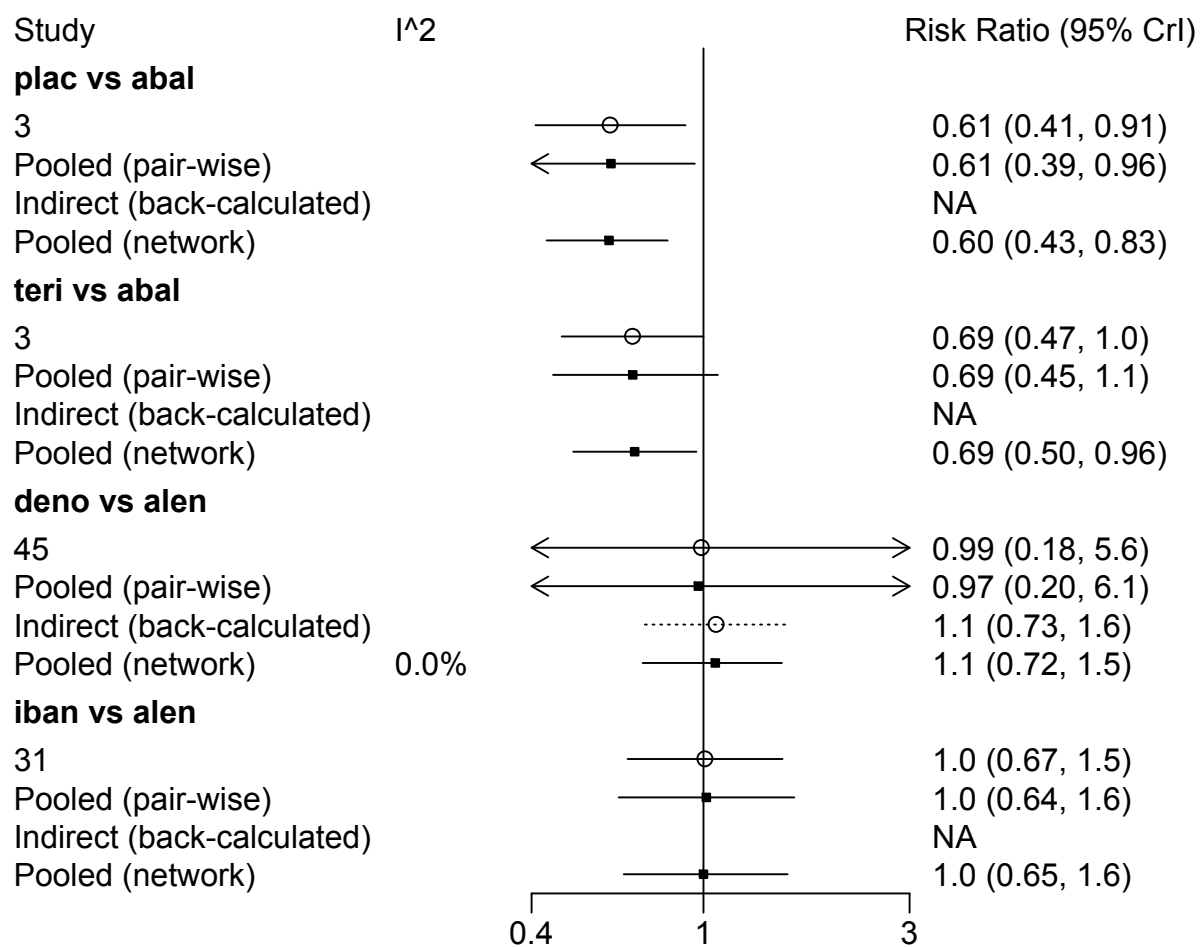

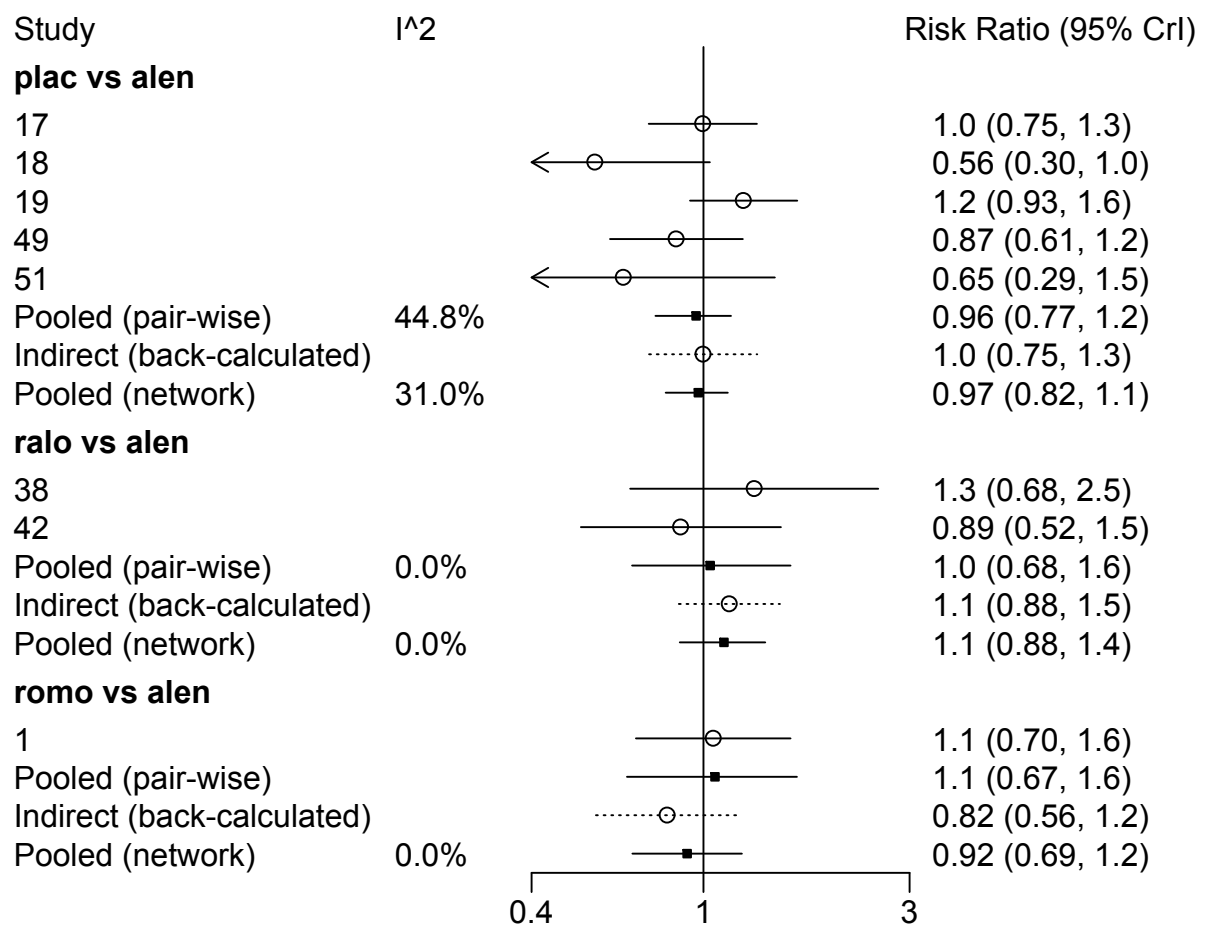

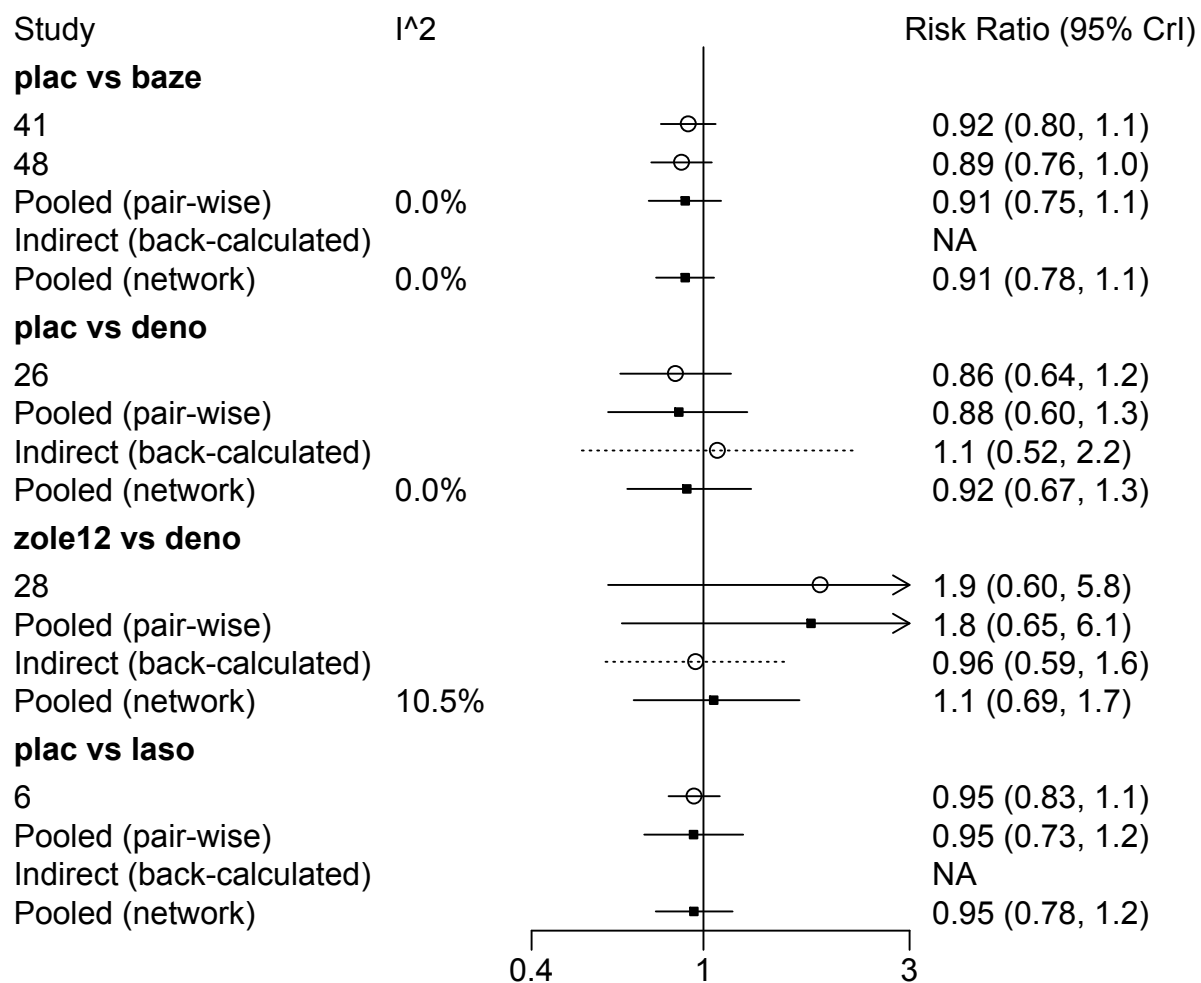

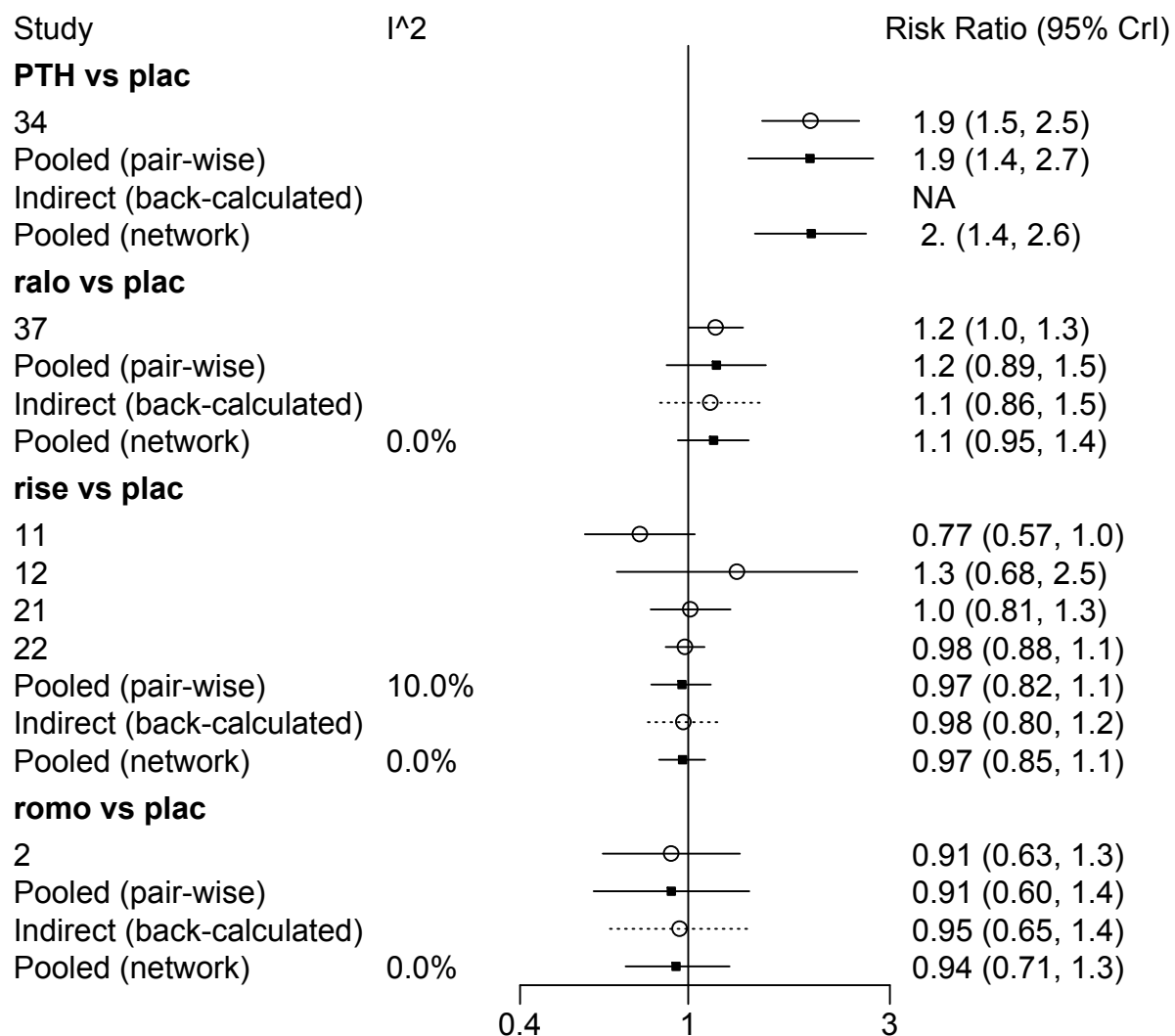

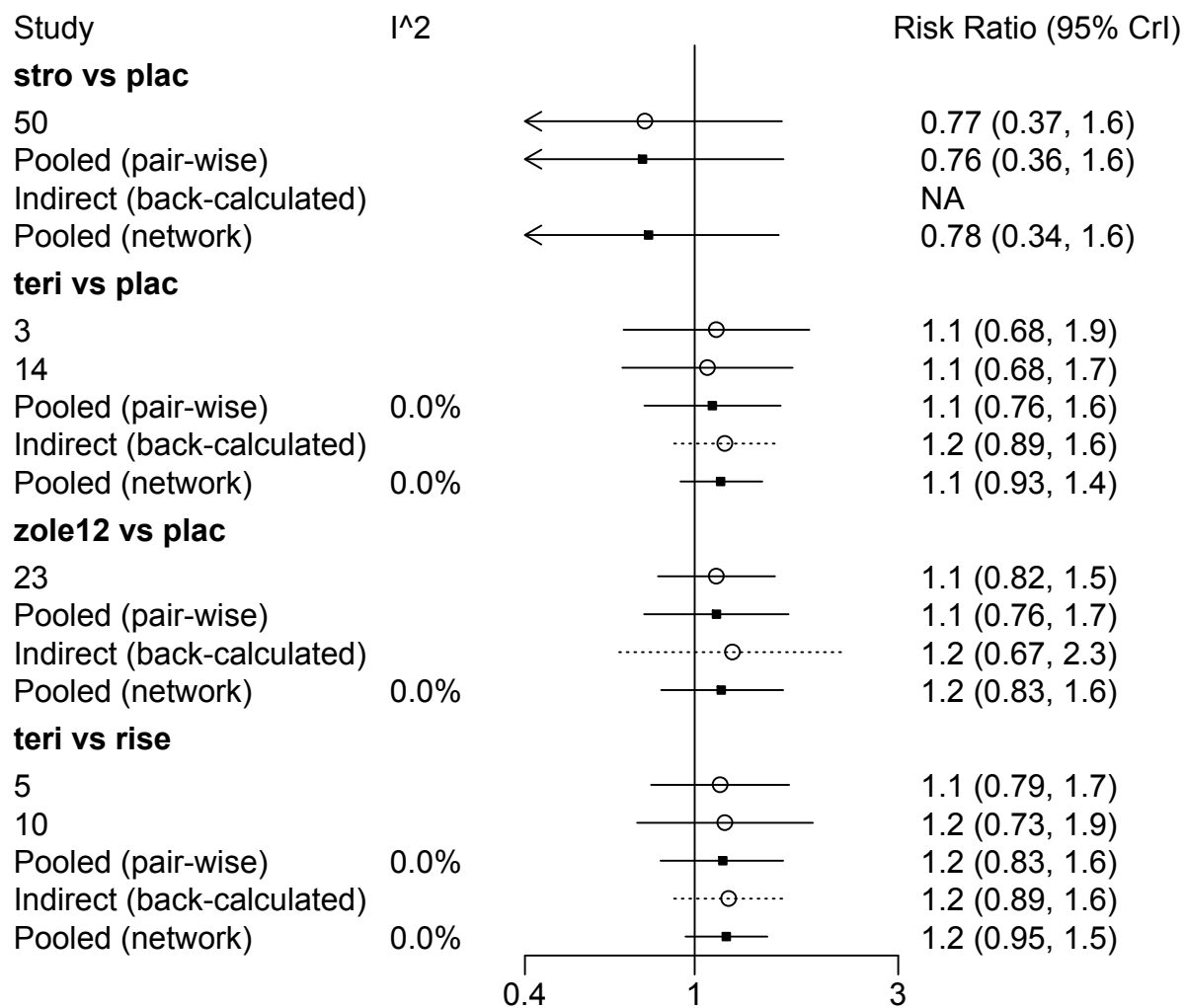

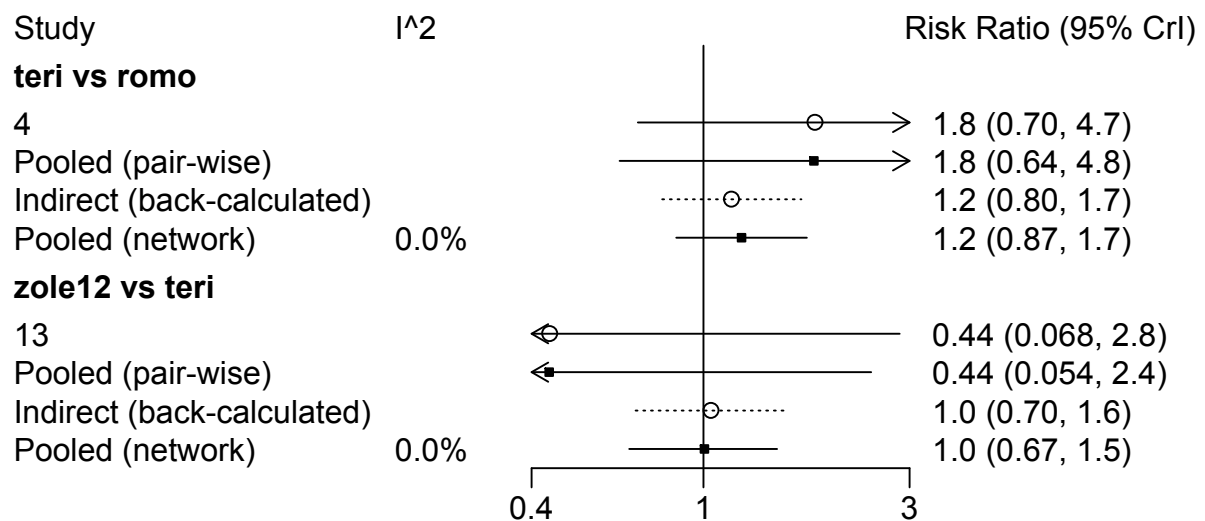

Supplement: S26 Appendix — (PDF) [file pone.0234123.s026.pdf]

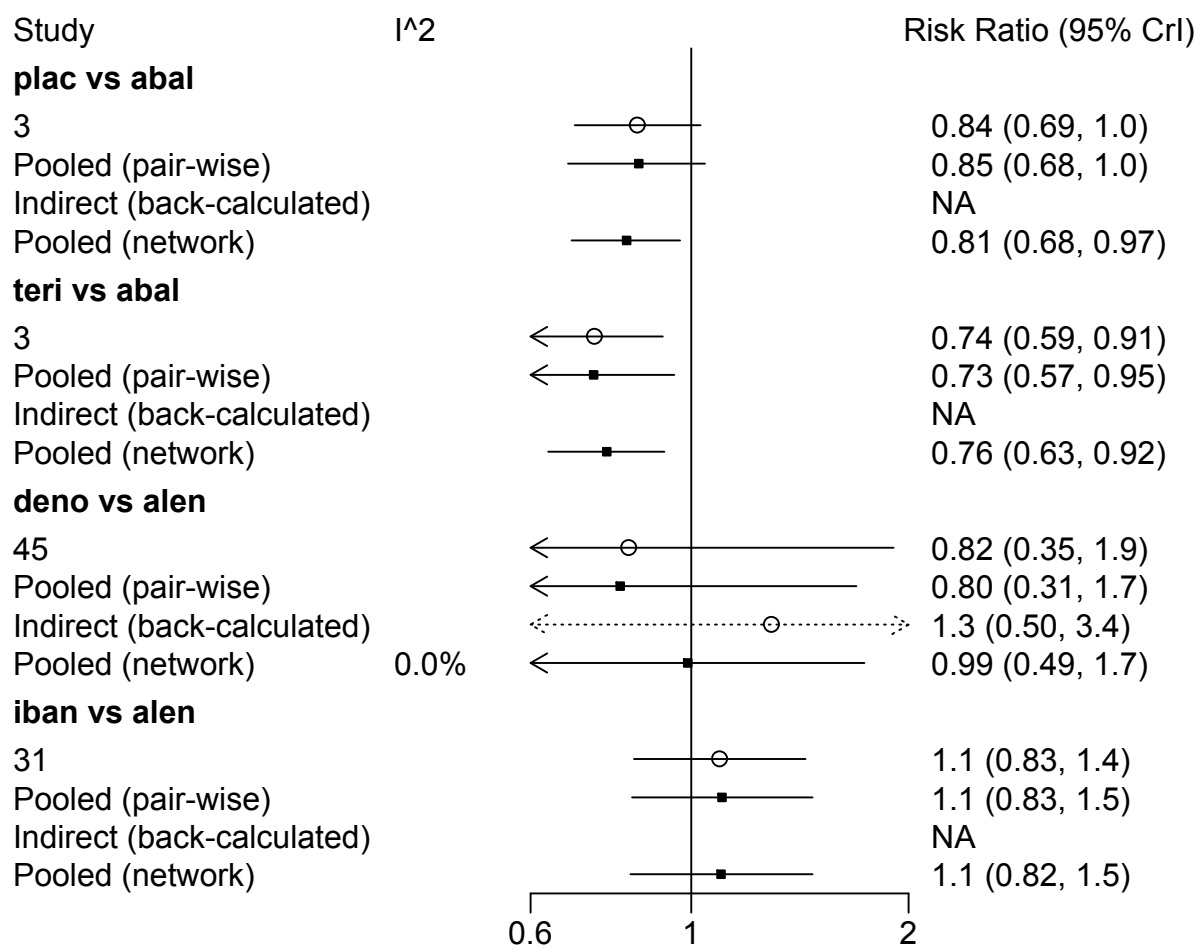

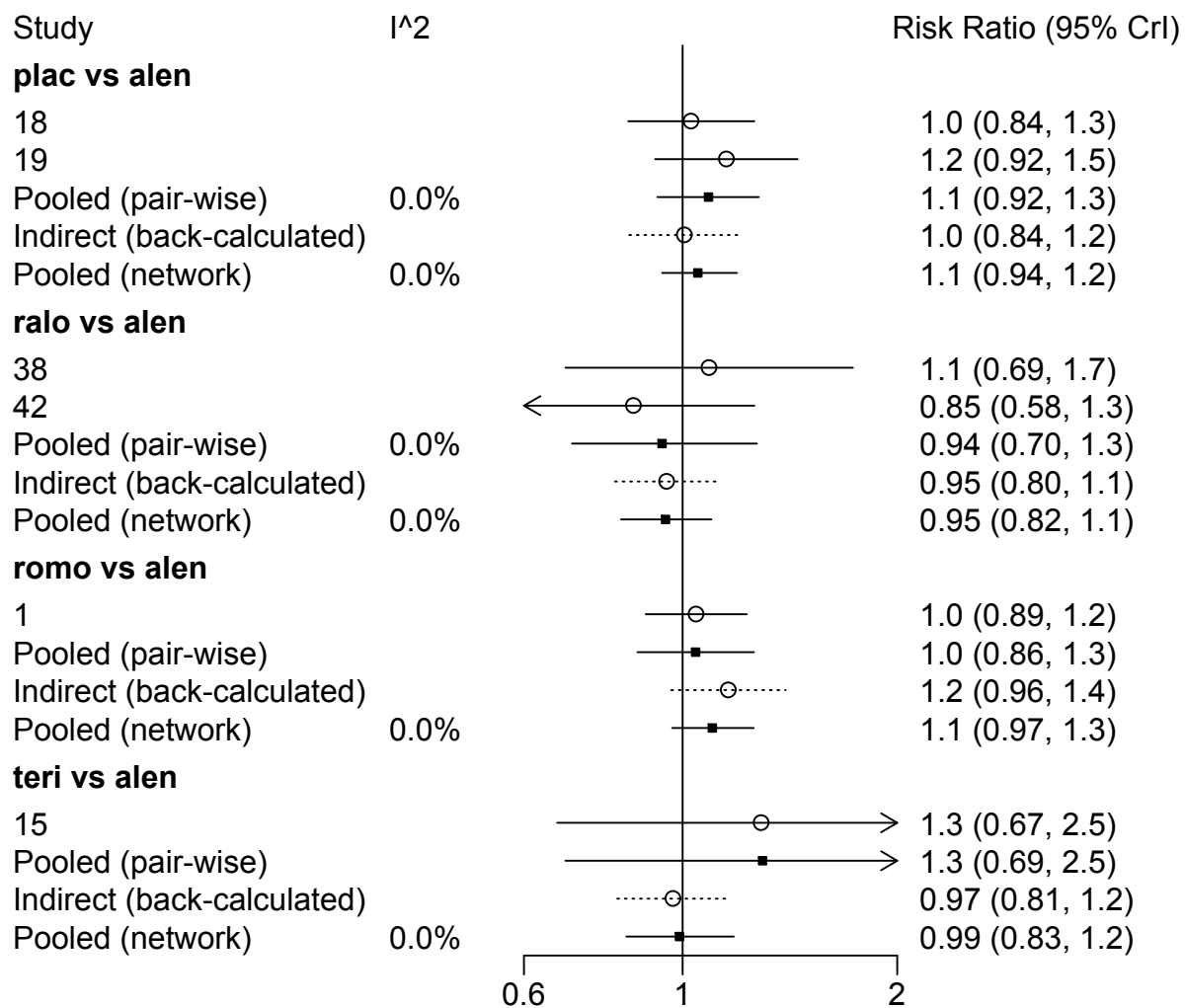

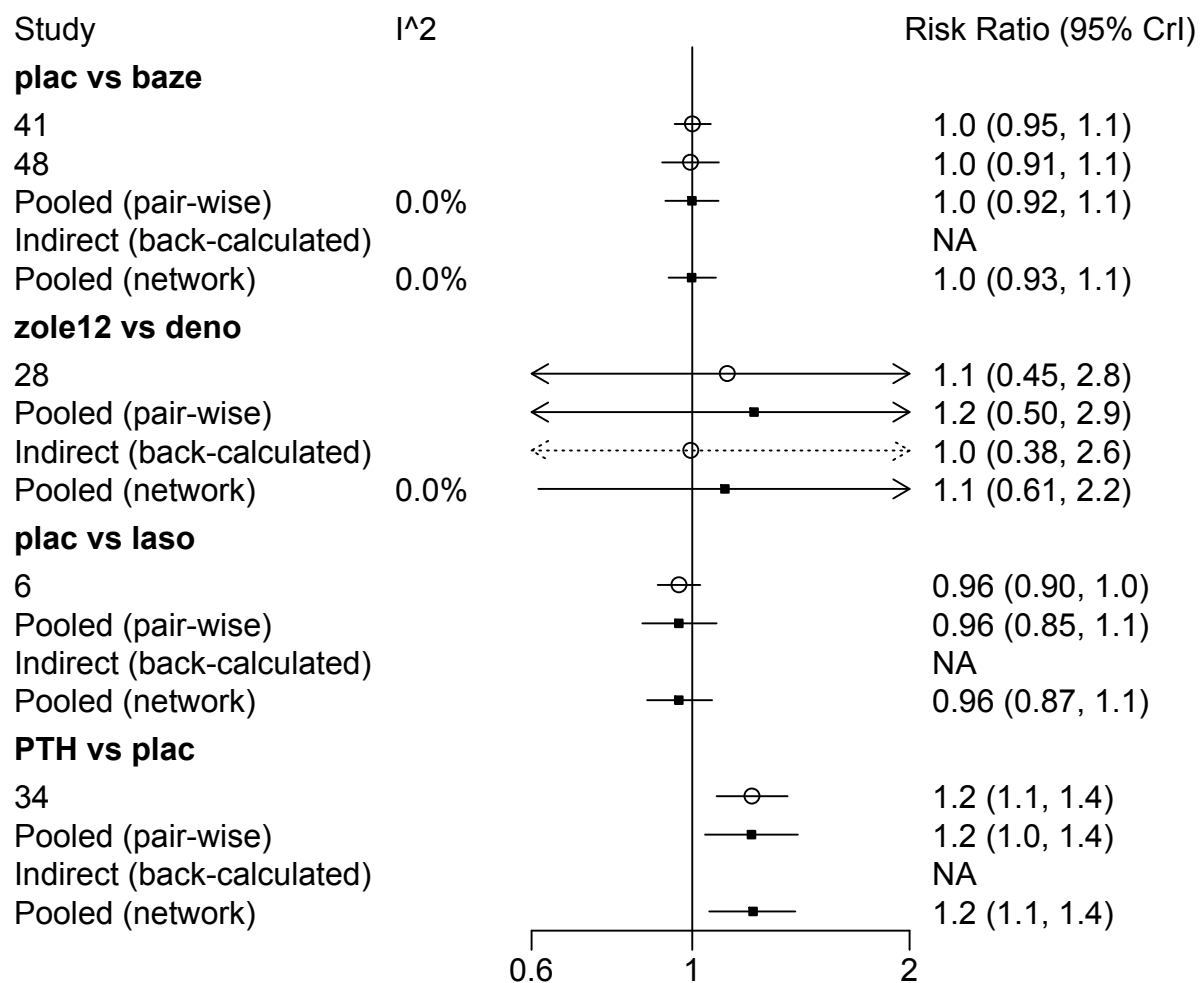

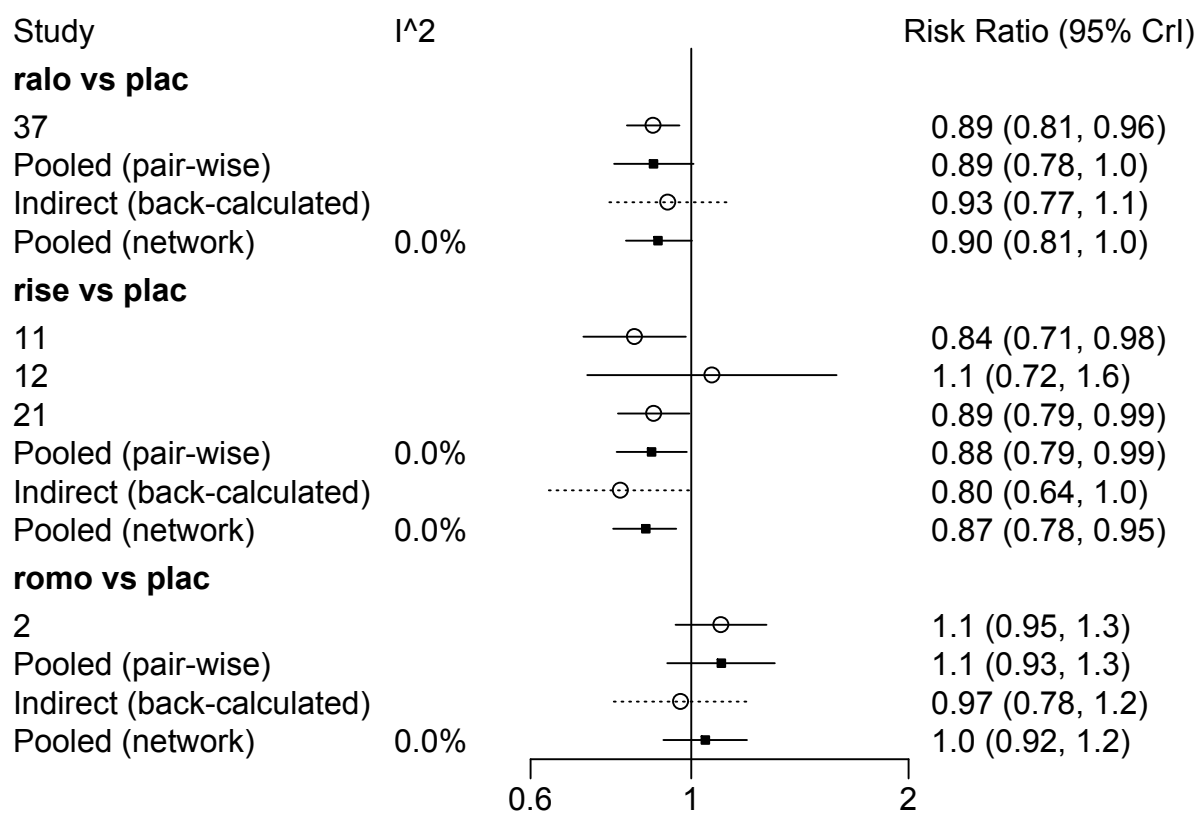

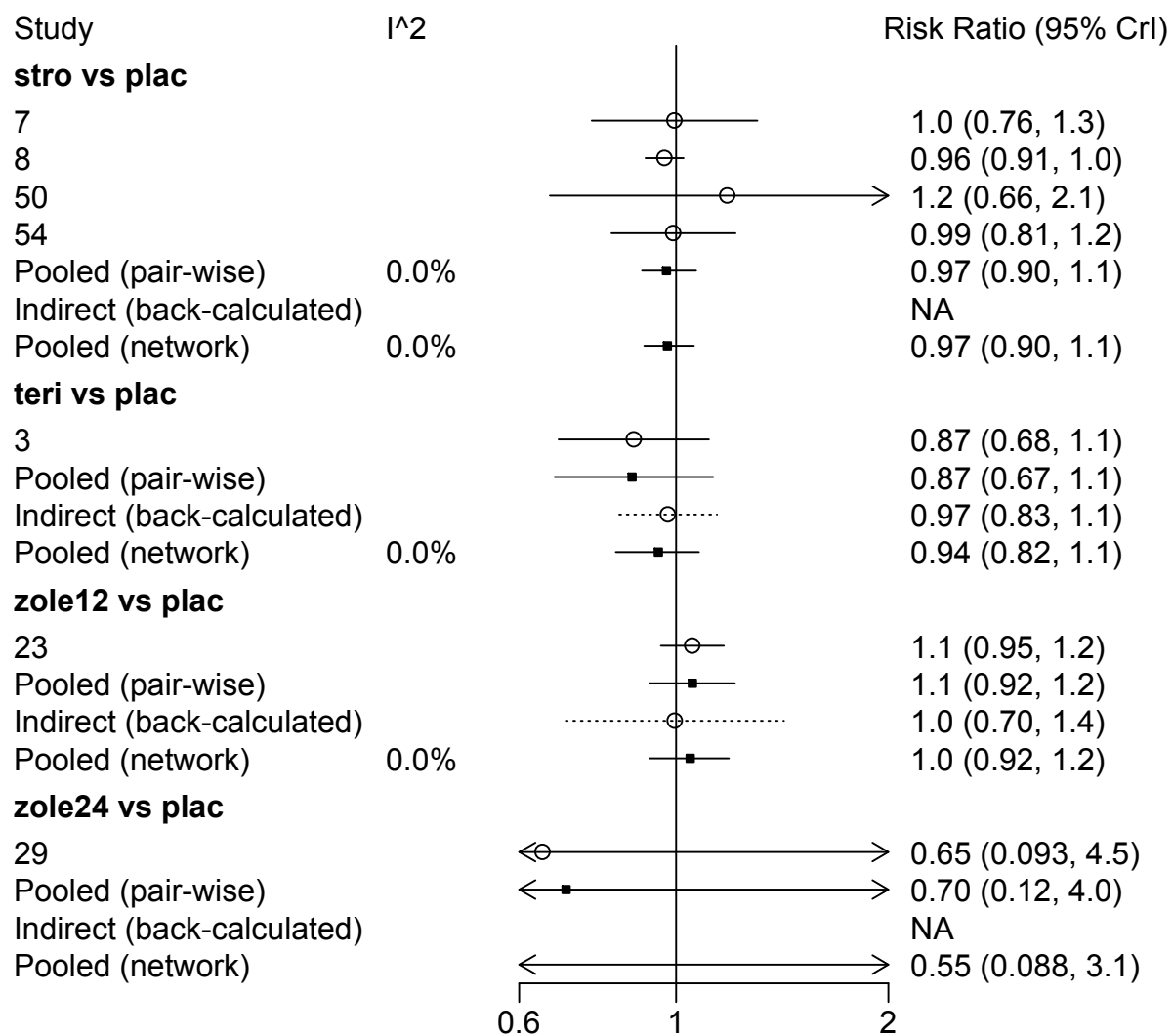

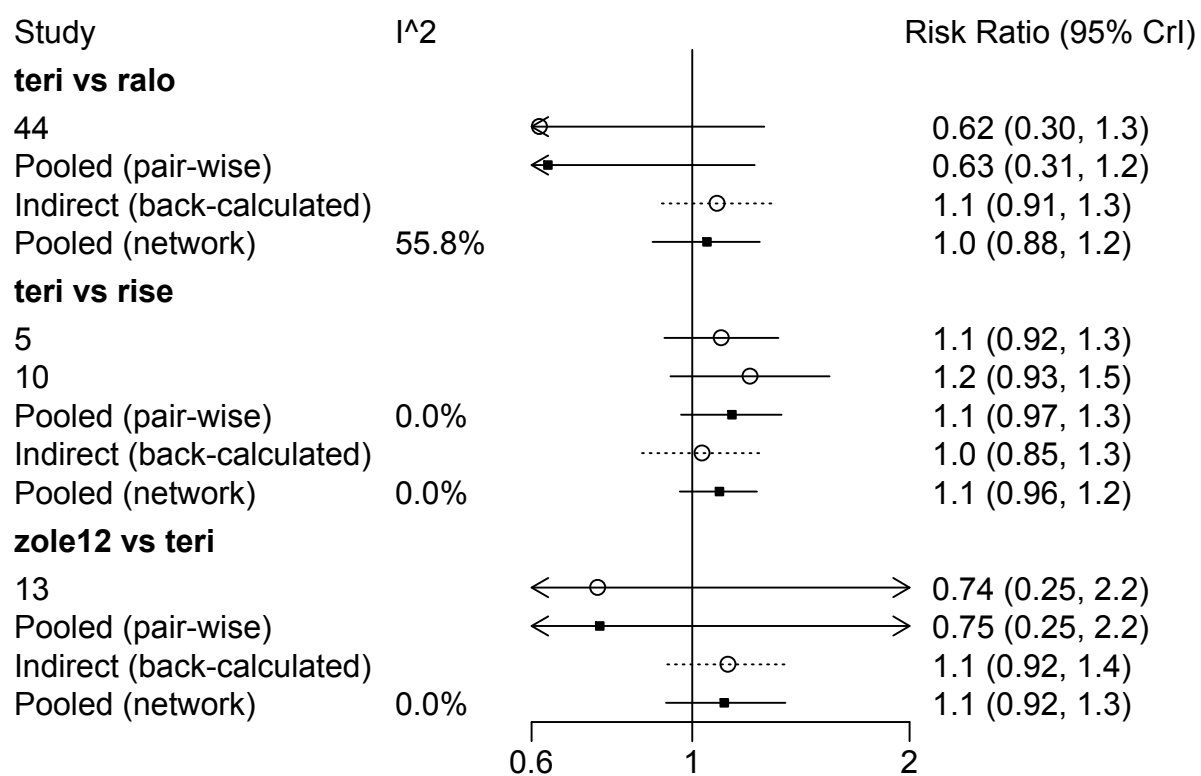

Supplement: S27 Appendix — (PDF) [file pone.0234123.s027.pdf]
