# Supplementary material for: Clinical efficacy and safety of drug interventions for primary and secondary prevention of osteoporotic fractures in postmenopausal women: Network meta-analysis followed by factor and cluster analysis
Source: PLoS One. 2020 Jun 3;15(6):e0234123. doi: 10.1371/journal.pone.0234123 (PMC7269244; doi:10.1371/journal.pone.0234123)
Supplement: S29 Appendix — (PDF) [file pone.0234123.s029.pdf]

```
* Encoding: UTF-8.

FACTOR
  /VARIABLES vf nvf tol acc
  /MISSING MEANSUB
  /ANALYSIS vf nvf tol acc
  /PRINT UNIVARIATE INITIAL CORRELATION SIG KMO EXTRACTION ROTATION FSCORE
  /CRITERIA MINEIGEN(1) ITERATE(25)
  /EXTRACTION PC
  /CRITERIA ITERATE(25)
  /ROTATION VARIMAX
  /SAVE REG(ALL)
  /METHOD=CORRELATION.
```

Factor Analysis

Descriptive Statistics

	Mean	Std. Deviation[a]	Analysis N[a]	Missing N
Vertebral fractures	.5000	.20485	10	3
Nonvertebral fractures	.5000	.23753	10	1
Tolerability	.5000	.20836	10	0
Acceptability	.5000	.18678	10	1

a. For each variable, missing values are replaced with the variable mean.

Correlation Matrix

		Vertebral fractures	Nonvertebral fractures	Tolerability	Acceptability
Correlation	Vertebral fractures	1.000	-.006	-.571	-.253
	Nonvertebral fractures	-.006	1.000	.225	-.391
	Tolerability	-.571	.225	1.000	.265
	Acceptability	-.253	-.391	.265	1.000
Sig. (1-tailed)	Vertebral fractures		.495	.090	.292
	Nonvertebral fractures	.495		.280	.149
	Tolerability	.090	.280		.229
	Acceptability	.292	.149	.229	

KMO and Bartlett's Test

Kaiser-Meyer-Olkin Measure of Sampling Adequacy.		.470
Bartlett's Test of Sphericity	Approx. Chi-Square	3.097
	df	6
	Sig.	.797

Communalities

	Initial	Extraction
Vertebral fractures	1.000	.718
Nonvertebral fractures	1.000	.826
Tolerability	1.000	.817
Acceptability	1.000	.746

Extraction Method: Principal Component Analysis.

Total Variance Explained

Component	Initial Eigenvalues			Extraction Sums of Squared ..	
	Total	% of Variance	Cumulative %	Total	% of Variance
1	1.751	43.770	43.770	1.751	43.770
2	1.356	33.907	77.678	1.356	33.907
3	.554	13.853	91.531		
4	.339	8.469	100.000		

Total Variance Explained

Component	Extraction Sums ...	Rotation Sums of Squared Loadings		
	Cumulative %	Total	% of Variance	Cumulative %
1	43.770	1.711	42.773	42.773
2	77.678	1.396	34.905	77.678
3				
4				

Extraction Method: Principal Component Analysis.

Component Matrix[a]

	Component	
	1	2
Vertebral fractures	-.833	-.153
Nonvertebral fractures	-.058	.907
Tolerability	.829	.359
Acceptability	.604	-.618

Extraction Method: Principal Component Analysis.

a. 2 components extracted.

Rotated Component Matrix[a]

	Component	
	1	2
Vertebral fractures	-.839	.120
Nonvertebral fractures	.233	.878
Tolerability	.901	.077
Acceptability	.376	-.778

Extraction Method: Principal Component Analysis.
Rotation Method: Varimax with Kaiser Normalization.

a. Rotation converged in 3 iterations.

Component Transformation Matrix

Component	1	2
1	.948	-.318
2	.318	.948

Extraction Method: Principal Component Analysis.
Rotation Method: Varimax with Kaiser Normalization.

Component Score Coefficient Matrix

	Component	
	1	2
Vertebral fractures	-.487	.044
Nonvertebral fractures	.181	.644
Tolerability	.533	.100
Acceptability	.182	-.541

Extraction Method: Principal Component Analysis.
Rotation Method: Varimax with Kaiser Normalization.
Component Scores.

Component Score Covariance Matrix

Component	1	2
1	1.000	.000
2	.000	1.000

Extraction Method: Principal Component Analysis.
Rotation Method: Varimax with Kaiser Normalization.
Component Scores.
