# Supplementary material for: Clinical efficacy and safety of drug interventions for primary and secondary prevention of osteoporotic fractures in postmenopausal women: Network meta-analysis followed by factor and cluster analysis
Source: PLoS One. 2020 Jun 3;15(6):e0234123. doi: 10.1371/journal.pone.0234123 (PMC7269244; doi:10.1371/journal.pone.0234123)
Supplement: S31 Appendix — (PDF) [file pone.0234123.s031.pdf]

```
* Encoding: UTF-8.

FACTOR
  /VARIABLES vf nvf tol acc
  /MISSING MEANSUB
  /ANALYSIS vf nvf tol acc
  /PRINT UNIVARIATE INITIAL CORRELATION SIG KMO EXTRACTION ROTATION FSCORE
  /CRITERIA MINEIGEN(1) ITERATE(25)
  /EXTRACTION PC
  /CRITERIA ITERATE(25)
  /ROTATION VARIMAX
  /SAVE REG(ALL)
  /METHOD=CORRELATION.
```

Factor Analysis

Descriptive Statistics

	Mean	Std. Deviation[a]	Analysis N[a]	Missing N
Vertebral fractures	.5000	.27227	15	0
Nonvertebral fractures	.5000	.24203	15	0
Tolerability	.5000	.22730	15	1
Acceptability	.5000	.23268	15	0

a. For each variable, missing values are replaced with the variable mean.

Correlation Matrix

		Vertebral fractures	Nonvertebral fractures	Tolerability	Acceptability
Correlation	Vertebral fractures	1.000	.796	-.480	-.401
	Nonvertebral fractures	.796	1.000	-.160	-.283
	Tolerability	-.480	-.160	1.000	.545
	Acceptability	-.401	-.283	.545	1.000
Sig. (1-tailed)	Vertebral fractures		.000	.035	.069
	Nonvertebral fractures	.000		.285	.153
	Tolerability	.035	.285		.022
	Acceptability	.069	.153	.022	

KMO and Bartlett's Test

Kaiser-Meyer-Olkin Measure of Sampling Adequacy.		.509
Bartlett's Test of Sphericity	Approx. Chi-Square	20.270
	df	6
	Sig.	.002

Communalities

	Initial	Extraction
Vertebral fractures	1.000	.906
Nonvertebral fractures	1.000	.940
Tolerability	1.000	.808
Acceptability	1.000	.733

Extraction Method: Principal Component Analysis.

Total Variance Explained

Component	Initial Eigenvalues			Extraction Sums of Squared ..	
	Total	% of Variance	Cumulative %	Total	% of Variance
1	2.353	58.837	58.837	2.353	58.837
2	1.033	25.833	84.670	1.033	25.833
3	.482	12.045	96.715		
4	.131	3.285	100.000		

Total Variance Explained

Component	Extraction Sums ...	Rotation Sums of Squared Loadings		
	Cumulative %	Total	% of Variance	Cumulative %
1	58.837	1.764	44.101	44.101
2	84.670	1.623	40.569	84.670
3				
4				

Extraction Method: Principal Component Analysis.

Component Matrix[a]

	Component	
	1	2
Vertebral fractures	.899	.314
Nonvertebral fractures	.757	.605
Tolerability	-.691	.574
Acceptability	-.703	.489

Extraction Method: Principal Component Analysis.

a. 2 components extracted.

Rotated Component Matrix[a]

	Component	
	1	2
Vertebral fractures	.879	-.367
Nonvertebral fractures	.968	-.056
Tolerability	-.131	.889
Acceptability	-.197	.833

Extraction Method: Principal Component Analysis.
Rotation Method: Varimax with Kaiser Normalization.

a. Rotation converged in 3 iterations.

Component Transformation Matrix

Component	1	2
1	.744	-.668
2	.668	.744

Extraction Method: Principal Component Analysis.
Rotation Method: Varimax with Kaiser Normalization.

Component Score Coefficient Matrix

	Component	
	1	2
Vertebral fractures	.487	-.029
Nonvertebral fractures	.631	.221
Tolerability	.153	.610
Acceptability	.094	.551

Extraction Method: Principal Component Analysis.
Rotation Method: Varimax with Kaiser Normalization.
Component Scores.

Component Score Covariance Matrix

Component	1	2
1	1.000	.000
2	.000	1.000

Extraction Method: Principal Component Analysis.
Rotation Method: Varimax with Kaiser Normalization.
Component Scores.
