# Supplementary material for: Clinical efficacy and safety of drug interventions for primary and secondary prevention of osteoporotic fractures in postmenopausal women: Network meta-analysis followed by factor and cluster analysis
Source: PLoS One. 2020 Jun 3;15(6):e0234123. doi: 10.1371/journal.pone.0234123 (PMC7269244; doi:10.1371/journal.pone.0234123)

A. Comparison-adjusted funnel plot of vertebral fractures in the primary prevention group.

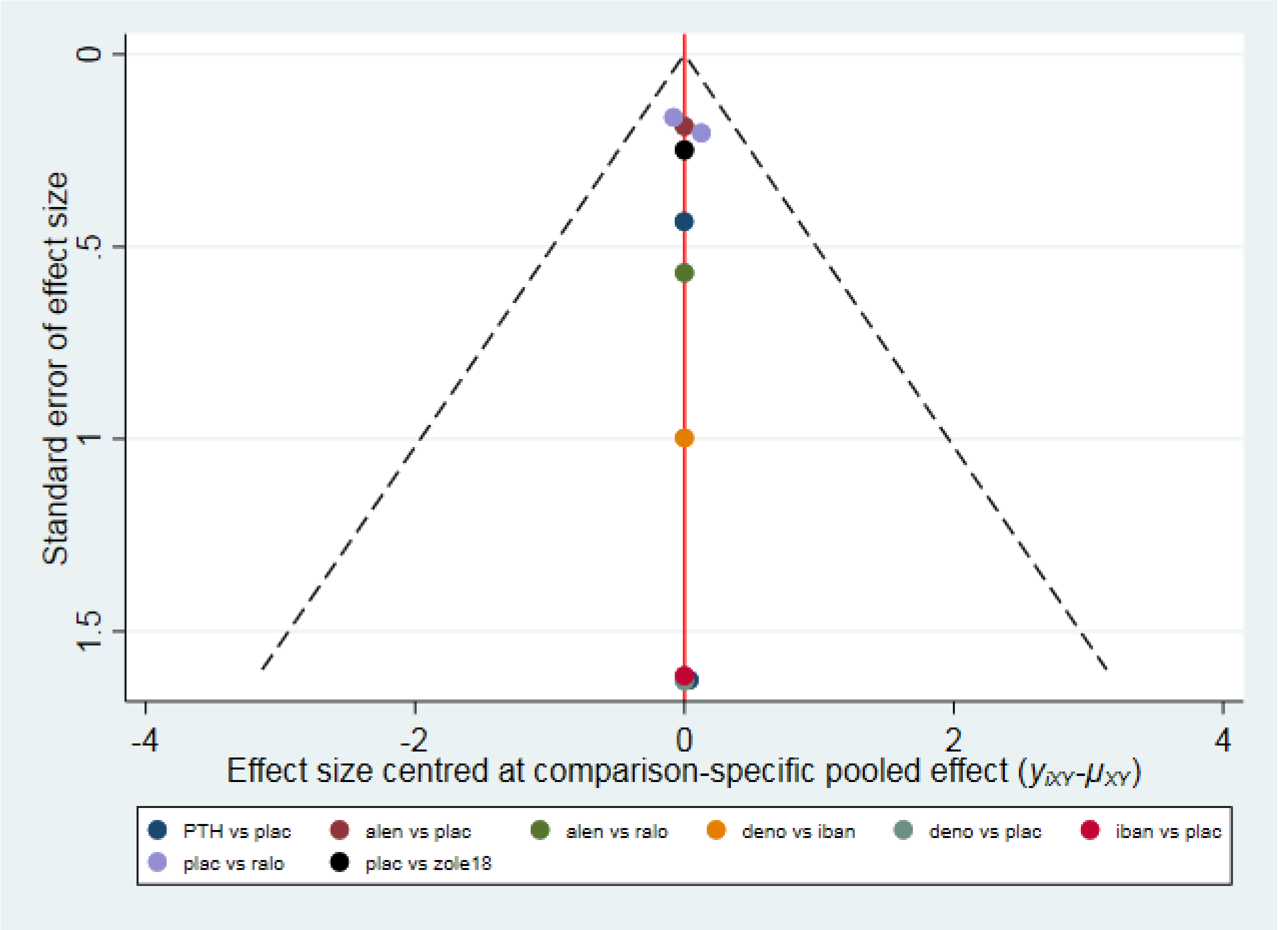

B. Comparison-adjusted funnel plot of nonvertebral fractures in the primary prevention group.

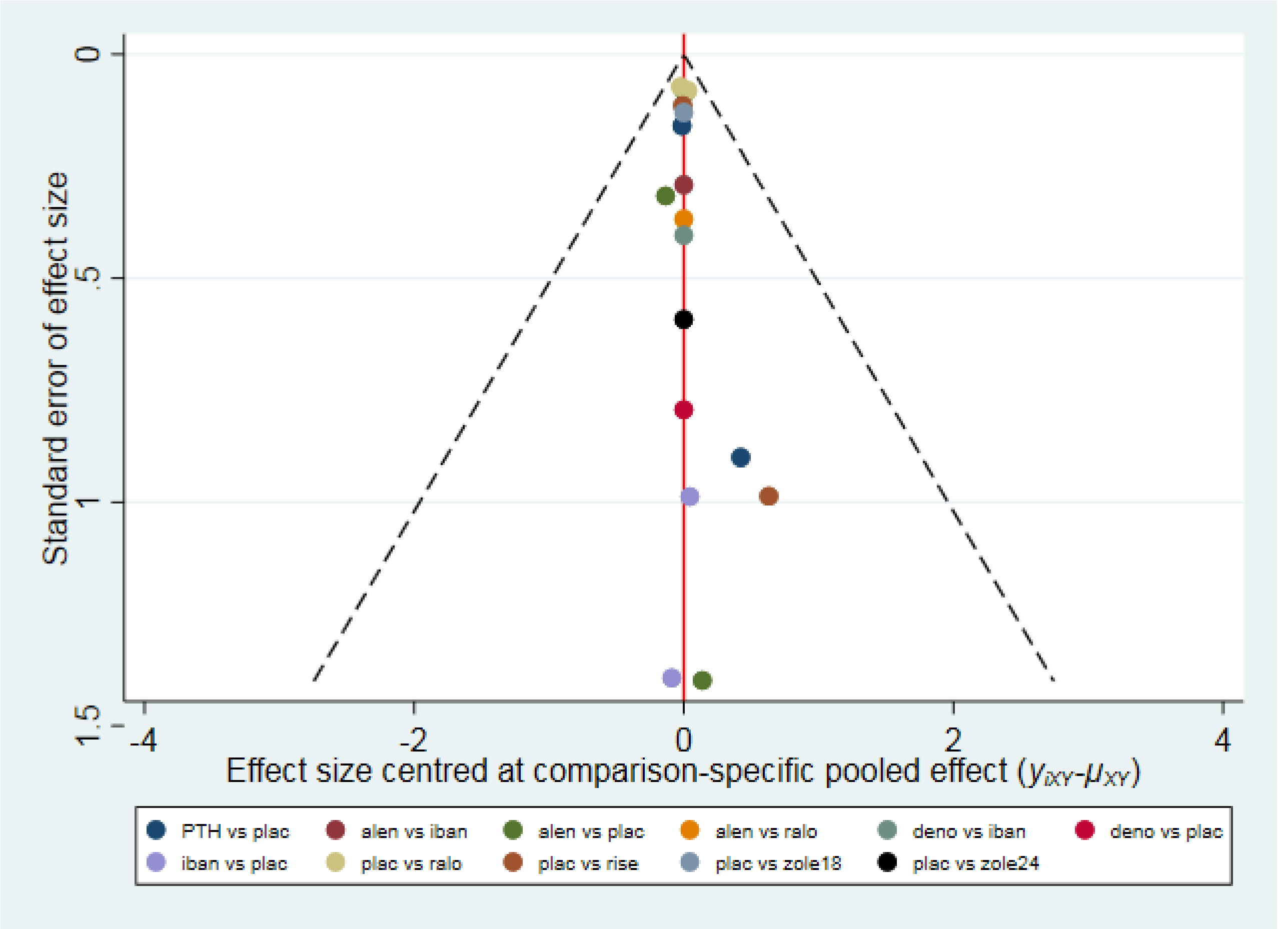

C. Comparison-adjusted funnel plot of tolerability in the primary prevention group.

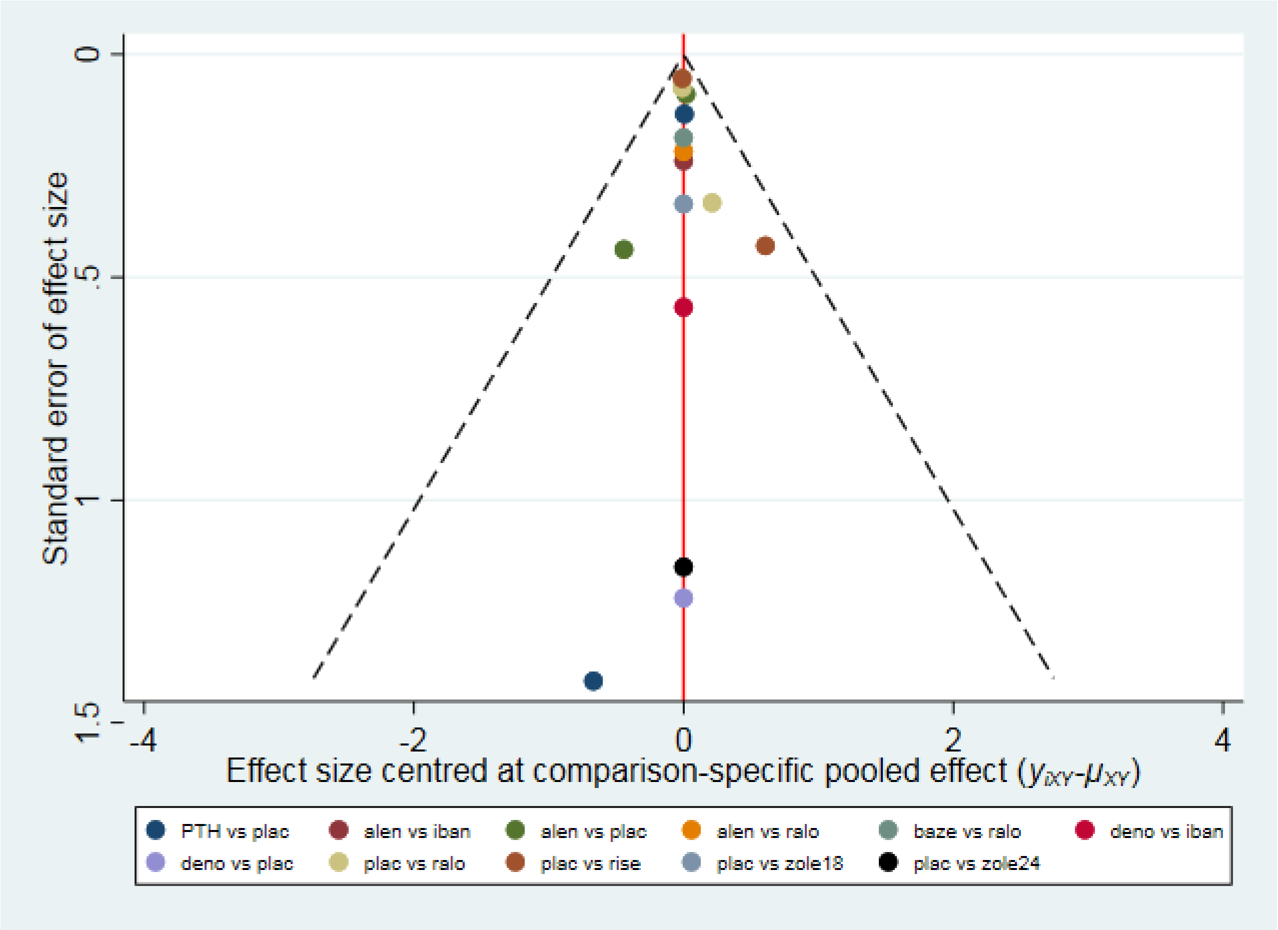

D.  Comparison-adjusted funnel plot of acceptability in the primary prevention group.

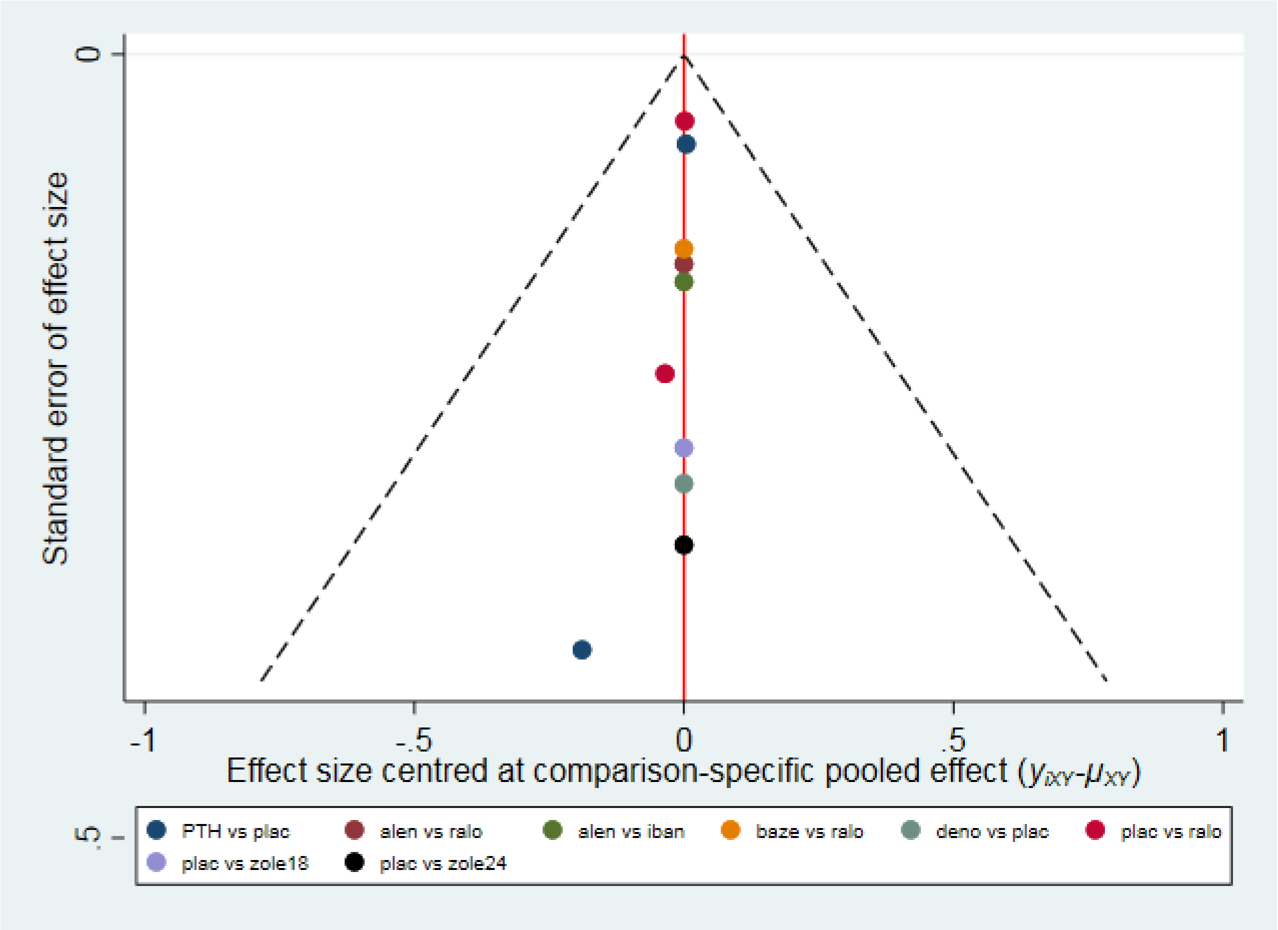

E. Comparison-adjusted funnel plot of vertebral fractures in the secondary prevention group.

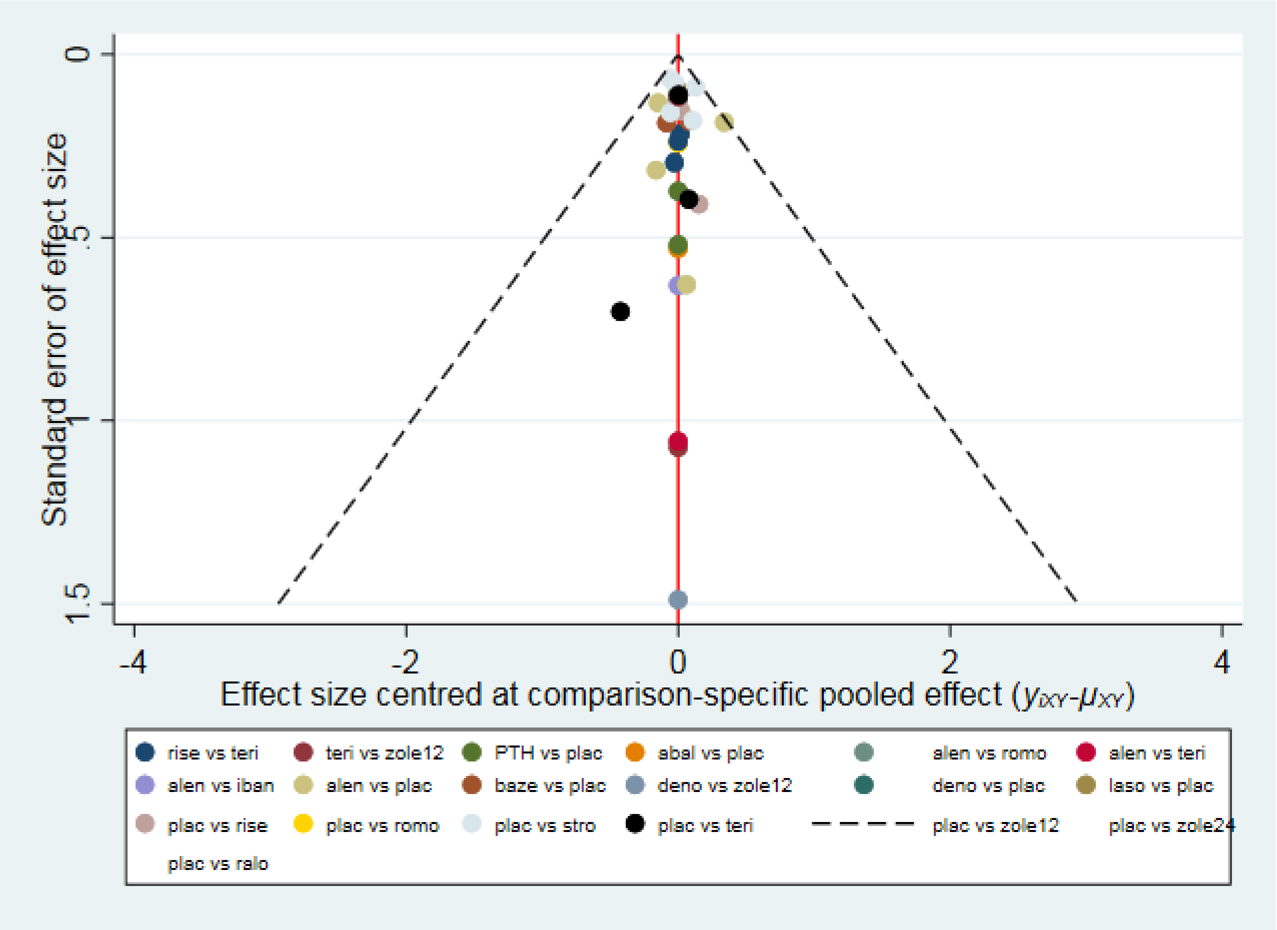

F. Comparison-adjusted funnel plot of nonvertebral fractures in the secondary prevention group.

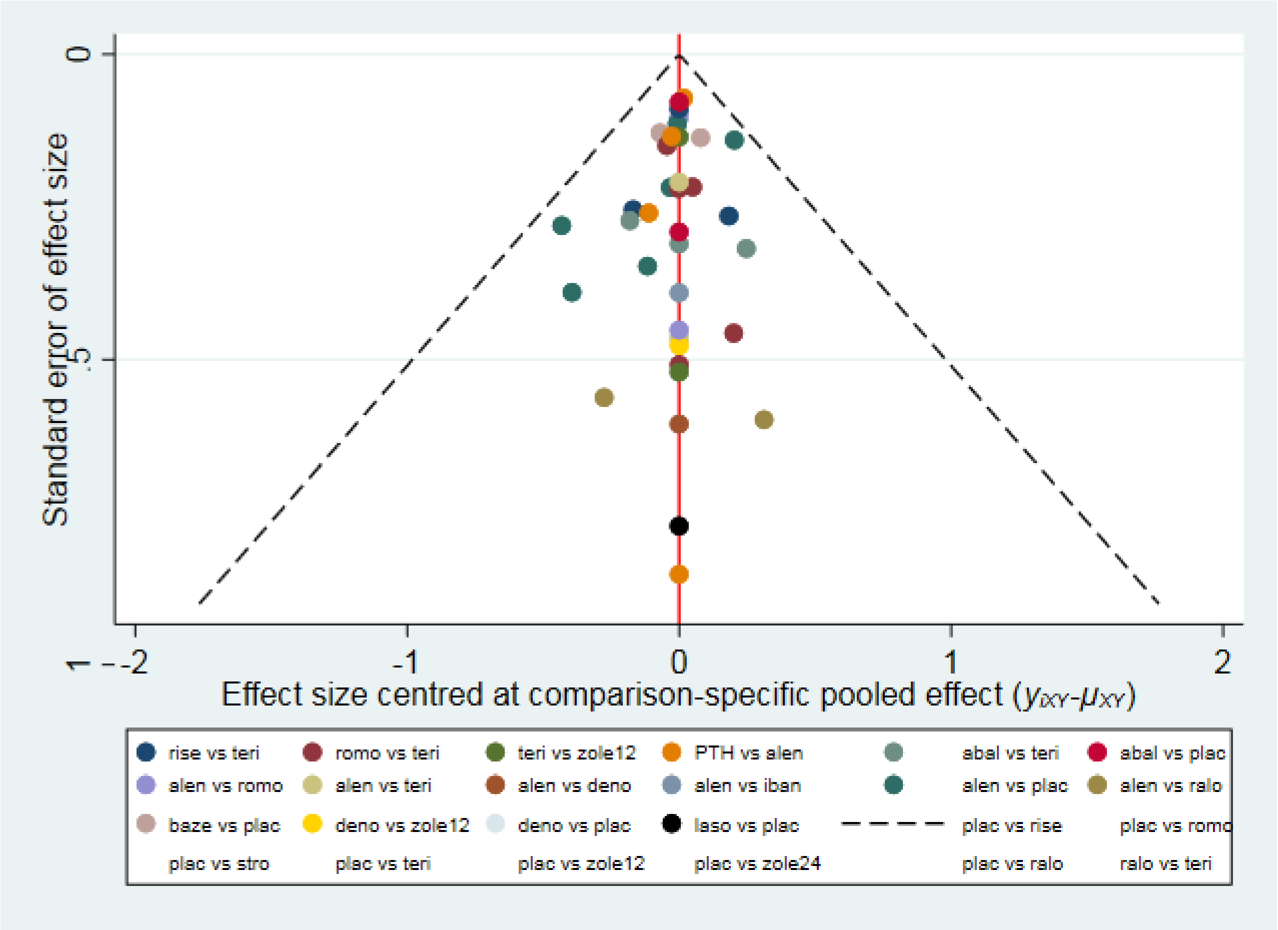

G. Comparison-adjusted funnel plot of tolerability in the secondary prevention group.

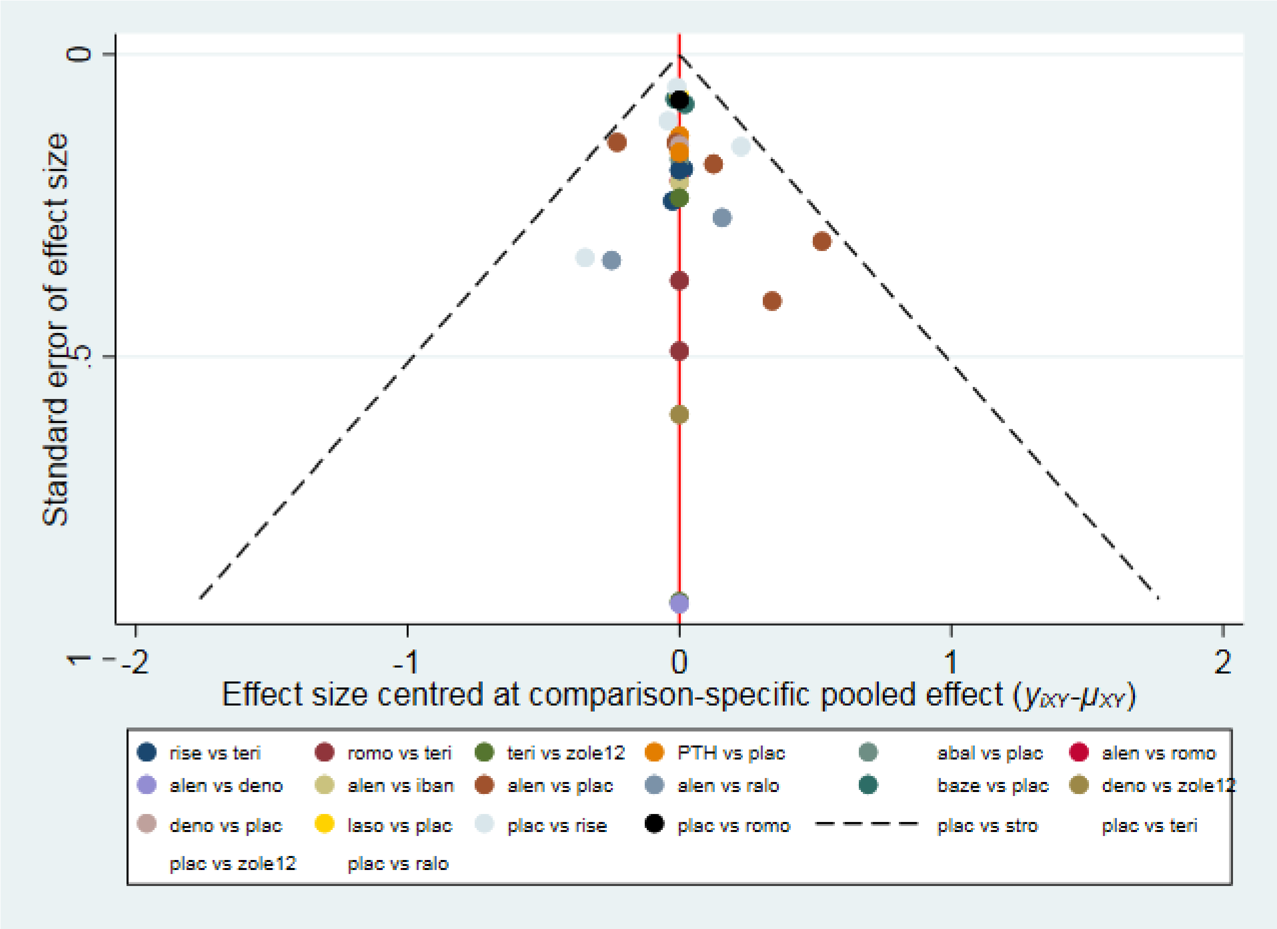

H. Comparison-adjusted funnel plot of acceptability in the secondary prevention group.

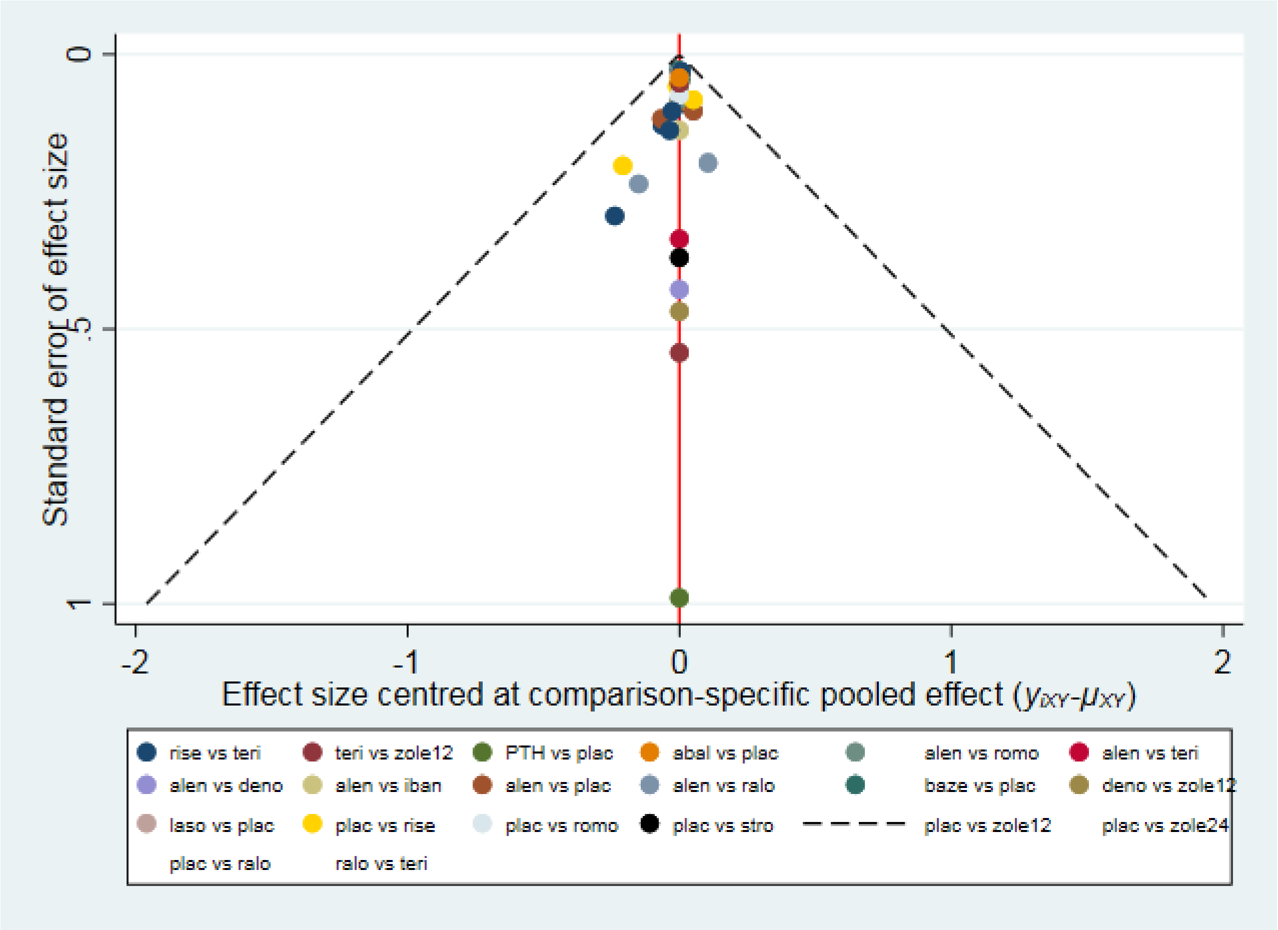

Supplement: S32 Appendix — (PDF) [file pone.0234123.s032.pdf]
