# Supplementary material for: Clinical efficacy and safety of drug interventions for primary and secondary prevention of osteoporotic fractures in postmenopausal women: Network meta-analysis followed by factor and cluster analysis
Source: PLoS One. 2020 Jun 3;15(6):e0234123. doi: 10.1371/journal.pone.0234123 (PMC7269244; doi:10.1371/journal.pone.0234123)
Supplement: Attachment — Submitted filename: Response to Reviewers.doc [file pone.0234123.s033.doc]

Dear Editor,

Thank you very much for sending us the valuable comments of the reviewers on our manuscript (No.: PONE-D-19-32748; Title: Clinical efficacy and safety of drug interventions for primary and secondary prevention of osteoporotic fractures in postmenopausal women: network meta-analysis followed by factor and cluster analysis), which have helped us to improve the quality of our paper greatly. We have thoroughly revised our manuscript according to those comments. All comments have been addressed and itemized as follows.

**Responses to Journal Requirements**

Question 1:

Please ensure that your manuscript meets PLOS ONE's style requirements, including those for file naming. The PLOS ONE style templates can be found at http://www.plosone.org/attachments/PLOSOne_formatting_sample_main_body.pdf and http://www.plosone.org/attachments/PLOSOne_formatting_sample_title_authors_affiliations.pdf.

Answer

We have revised title page, main body and references of our manuscript as well as file naming of supplementary materials according to PLOS ONE's style requirements.

Question 2:

In the Methods, please specify any assessment of risk of bias that may affect the cumulative evidence (e.g., publication bias, selective reporting within studies). Please ensure that the specific method of assessment (funnel plot, Egger's test, Begg's test, etc) is mentioned.

Answer

We have performed the detection of publication bias, and have added corresponding sentences into the methods, results, and discussion section in the revised manuscript.

Question 3:

Thank you for stating the following on the title page of your manuscript:

"Funding: No specific funding was received for this study."

We note that you have provided funding information that is not currently declared in your Funding Statement. However, funding information should not appear in the Acknowledgments section or other areas of your manuscript. We will only publish funding information present in the Funding Statement section of the online submission form.

Please remove any funding-related text from the manuscript and let us know how you would like to update your Funding Statement. Currently, your Funding Statement reads as follows: "NO".

Answer

No specific funding was received for this study, and accordingly, we have not provided funding information in the Funding Statement or in any section of the manuscript.

**Responses to Comments to the Author**

Question 1:

Is the manuscript technically sound, and do the data support the conclusions?

The manuscript must describe a technically sound piece of scientific research with data that supports the conclusions. Experiments must have been conducted rigorously, with appropriate controls, replication, and sample sizes. The conclusions must be drawn appropriately based on the data presented.

Reviewer #1: Partly

Answer

Although the process of data analyses in this paper was complex, we believe that every analysis step was logical and rigorous, which ensured the conclusions supported by the data.

Question 2:

Has the statistical analysis been performed appropriately and rigorously?

Reviewer #1: I Don't Know

Answer

The statistical analyses in this paper are complicated indeed. We performed network meta-analysis and calculated the SUCRA value for each treatment respectively in the primary prevention group and in the secondary prevention group at first. Then we did factor analysis based on the SUCRA values to obtain factor scores. At last, we conducted cluster analysis based on the factor scores to rank various interventions with both efficacy and safety taken into account. Although the statistical analyses are complex, we believe that they are logical and rigorous.

Question 3:

Have the authors made all data underlying the findings in their manuscript fully available?

The PLOS Data policy requires authors to make all data underlying the findings described in their manuscript fully available without restriction, with rare exception (please refer to the Data Availability Statement in the manuscript PDF file). The data should be provided as part of the manuscript or its supporting information, or deposited to a public repository. For example, in addition to summary statistics, the data points behind means, medians and variance measures should be available. If there are restrictions on publicly sharing data—e.g. participant privacy or use of data from a third party—those must be specified.

Reviewer #1: Yes.

Answer

None.

Question 4:

Is the manuscript presented in an intelligible fashion and written in standard English?

PLOS ONE does not copyedit accepted manuscripts, so the language in submitted articles must be clear, correct, and unambiguous. Any typographical or grammatical errors should be corrected at revision, so please note any specific errors here.

Reviewer #1: No.

Answer

We have made every effort to revise the manuscript in terms of English grammar, sentence structure, and spelling. If something wrong still exists in these aspects, please point out them and we will try our best to revise them again.

Question 5:

Review Comments to the Author

Please use the space provided to explain your answers to the questions above. You may also include additional comments for the author, including concerns about dual publication, research ethics, or publication ethics. (Please upload your review as an attachment if it exceeds 20,000 characters)

Reviewer #1: The authors have made a substantial job in summarizing evidence from RCTs on drug intervention for the prevention of osteoporotic fractures.

The manuscript is rather technical and some aspects of the analysis of data can be difficult to follow. The English of this article is rather good but could be further improved by English editing.

Answer:

Thank you very much for the positive comments.

The statistical analyses in this paper are complicated indeed. We performed network meta-analysis and calculated the SUCRA value for each treatment respectively in the primary prevention group and in the secondary prevention group at first. Then we did factor analysis based on the SUCRA values to obtain factor scores. At last, we conducted cluster analysis based on the factor scores to rank various interventions with both efficacy and safety taken into account. Although the statistical analyses are complex, we believe that they are logical and rigorous. Meanwhile, we have provided detailed methods and results in the manuscript for all data analyses to make them clear to readers. On the other hand, we have made every effort to revise the manuscript in terms of English grammar, sentence structure, and spelling. If something wrong still exists in these aspects, please point out them and we will try our best to revise them again.

Reviewer #1: I have some comments:

When judging the evidence (e.g. figure 5), is uncertainty taken properly into account? At the bottom of page 8, Cluster 1 and 2 are described, but these seem determined based on the estimates presented in the figure. Are the differences statistically significant?

Answer:

This is a good point.

Although the cluster analysis in the paper ensured maximum intracluster similarity and minimum intercluster similarity according to the maximum of clustering gain [1], it failed to tell us whether the difference among various clusters was statistically significant or not. This point has been added into the fourth point in the limitations section of the revised manuscript.

**References**

1.	Jung Y, Park H, Du D, Drake B. A Decision Criterion for the Optimal Number of Clusters in Hierarchical Clustering. J GLOBAL OPTIM. 2003; 25(1):91-111.

Reviewer #1: Tolerability is defined as withdrawals due to adverse events. However, it does not take into account the severity of the adverse effect. This is acknowledged by the authors in the discussion, but should it also be taken into account when summarizing the main findings concerning safety?

Answer:

This is a good point.

Given that the evidence summary section in the paper mainly stated key findings and comparisons with previous studies, we have added this point into the fifth point in the limitations section.

Reviewer #1: Figs 2A-2H are very nice, but it is unclear how they should be judged. ‘evidence loop in the network plot’ could be defined more clearly. Is it purely the visual inspection saying that there was an evidence loop or no.

Answer:

“Evidence loop in the network plot” means “closed loop in the network plot”, and that it exists in the network plot means the necessity of test for inconsistency. There is no closed loop in Fig 2D, which meant that it was not necessary to conduct test of inconsistency for the outcome of acceptability in the primary prevention group. In network plots, the size of every circle is proportional to the number of randomly assigned participants and indicates the sample size; and the width of the lines corresponds to the number of trials. Thus, those network plots also tell us: placebo was most commonly compared to other interventions in each network plot; on the contrary, the lines among active drugs were thin, and the number of those lines was limited, which suggested the lack of head-to-head trials comparing active drugs. Accordingly, we have revised the third paragraph in the results section and the legend of Fig 2. Meanwhile, the fact of the lack of head-to-head trials was clarified in the fourth point in the limitations section.

Reviewer #1: Did you consider including hip fracture as an additional endpoint. If not, this should be noted as a limitation of the study.

Answer:

This limitation of the paper has been added into the last point in the limitations section.

Question 6:

PLOS authors have the option to publish the peer review history of their article (what does this mean?). If published, this will include your full peer review and any attached files.

If you choose “no”, your identity will remain anonymous but your review may still be made public.

Do you want your identity to be public for this peer review? For information about this choice, including consent withdrawal, please see our Privacy Policy.

Reviewer #1: No

Answer

None.

Hopefully, we have made an appropriate revision based on these valuable comments and suggestions. Please let me know if there is anything else we need to do with the revision, and we’ll complete it as soon as possible.

We thank you wholeheartedly for your excellent work. Your kind assistance is greatly appreciated. We look forward to any future correspondence.

Yours sincerely,

Liangliang Ding

E-mail: 15887847798m0@sina.cn
