# Supplementary material for: Clinical efficacy and safety of drug interventions for primary and secondary prevention of osteoporotic fractures in postmenopausal women: Network meta-analysis followed by factor and cluster analysis
Source: PLoS One. 2020 Jun 3;15(6):e0234123. doi: 10.1371/journal.pone.0234123 (PMC7269244; doi:10.1371/journal.pone.0234123)
Supplement: Attachment — Submitted filename: Response to Reviewers.doc [file pone.0234123.s034.doc]

Dear Editor,

Thank you very much for sending us the valuable comments of the reviewers on our manuscript (No.: PONE-D-19-32748R1; Title: Clinical efficacy and safety of drug interventions for primary and secondary prevention of osteoporotic fractures in postmenopausal women: network meta-analysis followed by factor and cluster analysis), which have helped us to improve the quality of our paper greatly. We have thoroughly revised our manuscript according to those comments. All comments have been addressed and itemized as follows.

**Responses to Journal Requirements**

To enhance the reproducibility of your results, we recommend that if applicable you deposit your laboratory protocols in protocols.io, where a protocol can be assigned its own identifier (DOI) such that it can be cited independently in the future. For instructions see: http://journals.plos.org/plosone/s/submission-guidelines#loc-laboratory-protocols.

Answer

We have deposited the study protocol for this network meta-analysis in protocols.io, and the DOI of the study protocol has been presented in the first paragraph of the METHODS section.

**Responses to Comments to the Author**

Question 1:

If the authors have adequately addressed your comments raised in a previous round of review and you feel that this manuscript is now acceptable for publication, you may indicate that here to bypass the “Comments to the Author” section, enter your conflict of interest statement in the “Confidential to Editor” section, and submit your "Accept" recommendation.

Reviewer #1: (No Response)

Reviewer #2: (No Response)

Answer

None.

Question 2:

Is the manuscript technically sound, and do the data support the conclusions?

The manuscript must describe a technically sound piece of scientific research with data that supports the conclusions. Experiments must have been conducted rigorously, with appropriate controls, replication, and sample sizes. The conclusions must be drawn appropriately based on the data presented.

Reviewer #1: Yes

Reviewer #2: Yes

Answer

Thanks very much for these positive responses.

Question 3:

Has the statistical analysis been performed appropriately and rigorously?

Reviewer #1: I Don't Know

Reviewer #2: Yes.

Answer

This point has been detailed in the first response letter, and we believe the statistical analyses in this paper were logical and rigorous although they were complicated. Thanks very much for the positive response of Reviewer #2.

Question 4:

Have the authors made all data underlying the findings in their manuscript fully available?

The PLOS Data policy requires authors to make all data underlying the findings described in their manuscript fully available without restriction, with rare exception (please refer to the Data Availability Statement in the manuscript PDF file). The data should be provided as part of the manuscript or its supporting information, or deposited to a public repository. For example, in addition to summary statistics, the data points behind means, medians and variance measures should be available. If there are restrictions on publicly sharing data—e.g. participant privacy or use of data from a third party—those must be specified.

Reviewer #1: Yes

Reviewer #2: Yes.

Answer

Thanks very much for these positive responses.

Question 5:

Is the manuscript presented in an intelligible fashion and written in standard English?

PLOS ONE does not copyedit accepted manuscripts, so the language in submitted articles must be clear, correct, and unambiguous. Any typographical or grammatical errors should be corrected at revision, so please note any specific errors here.

Reviewer #1: Yes

Reviewer #2: Yes.

Answer

Thanks very much for these positive responses.

Question 6:

Review Comments to the Author

Please use the space provided to explain your answers to the questions above. You may also include additional comments for the author, including concerns about dual publication, research ethics, or publication ethics. (Please upload your review as an attachment if it exceeds 20,000 characters).

Reviewer #1: Thank you for the revised version of the manuscript.

I appreciate that you have included in the limitation section that:

'Meanwhile, the two safety outcomes failed to consider the severity of adverse effects.'

(I suggest omitting 'Meanwhile')

This limitation should also be indicated in the abstract and other places where the main findings are summarized. For example, when it is stated (abstract) that: 'Romosozumab, teriparatide, denosumab and risedronate, with the greatest composite scores, constituted the optimal cluster having both superior efficacy and superior safety', the words 'superior safety is a very strong statement. Many reads do not read the limitation section.

Answer:

We have omitted 'Meanwhile' in the sentence of 'Meanwhile, the two safety outcomes failed to consider the severity of adverse effects'.

We have added this limitation into the ABSTRACT section and into the second paragraph of the DISCUSSION section.

Reviewer #1: You have also mention some other very relevant limitation. They should also influence on the rest of the discussion.

It is stated on page 11: 'Fortunately, we have identified the newest evidence on zoledronate intravenously used at a dose of 5 mg per 18 months, which nowadays is the only one drug intervention that has the capability to significantly reduce both vertebral (RR 0.46) and nonvertebral fracture risk (HR 0.66) for primary prevention of PMO'

COMMENT: As you indicate in the limitation section, this could be due to lack of studies/statistical power. I therefor suggest that you reformulate to 'which is the only drug which has been shown to significantly reduce....'.

Answer:

We have revised this sentence. Meanwhile, we have revised the sentences relevant with this point in the ABSTRACT and CONCLUSIONS sections.

Reviewer #1: In the abstract, SUCRA should be defined.

Answer:

SUCRA has been defined in the ABSTRACT section.

Reviewer #2: This study provided a network meta-analysis (NMA) that enabled the authors to pool data from 57 individual studies that included evidence from both direct and indirect comparisons of the 15 interventions for two efficacy outcomes and two safety outcomes.

The surface under the cumulative ranking curve (SUCRA) is a numeric presentation of the overall ranking of an intervention and presents a single number associated with each treatment. SUCRA values range from 0 to 1. Higher SUCRA values indicate that the intervention was more likely to be in the top rank while lower SUCRA values indicate that the intervention likely to be in the lower rank.

Since the SUCRA values were used to rank the interventions as well as used in the subsequent factor and cluster analyses it is important to consider their quality. There are a few problem associated with SUCRA values (1) some of which the authors have addressed.

(1) Mbuagbaw, L., Rochwerg, B., Jaeschke, R. et al. Approaches to interpreting and choosing the best treatments in network meta-analyses. Syst Rev 6, 79 (2017). https://doi.org/10.1186/s13643-017-0473-z

First, the evidence on which the SUCRA rankings are based may be of very low quality and therefore untrustworthy. The authors have provided the Jadad scores along with the data corresponding to all of the selected studies. They have also provided visual representation of NMA point estimates and certainty intervals comparing estimates of each treatment against placebo in the main text as well as the comparisons among the treatments in the Supplemental files.

Second, there are typically several relevant outcomes. A treatment that is best in one outcome (say, an efficacy outcome) may be the worst in another outcome (for example, a safety outcome). The authors were able to combine efficacy and safety outcomes for the secondary prevention group via factor/cluster analyses to attempt to address this and thereby provide rankings for the interventions based on all outcomes.

All in all it appears that all of the methods associated with this NMA are appropriate well described. The presentations of the assessments of heterogeneity and publication bias are important aids for the interpretation of the results.

Answer:

Thanks very much for these positive comments.

Reviewer #2: There are a few problems that were not addressed and might be good to include in the Discussion or the Limitations section. Issues such as cost and a clinician’s familiarity with use of a particular treatment may also bear consideration when ranking interventions. Also, in the process of calculation, SUCRA does not consider the magnitude of differences in effects between treatments (e.g. the first ranked treatment may be only slightly, or a great deal better than the second ranked treatment). And finally, chance may explain any apparent difference between treatments, and SUCRA does not capture that possibility.

Answer:

The first point has been added into the last paragraph of the Clinical Implications section in the DISCUSSION section. The second and third points have been added into the last point of the Limitations section.

Question 7:

PLOS authors have the option to publish the peer review history of their article (what does this mean?). If published, this will include your full peer review and any attached files.

If you choose “no”, your identity will remain anonymous but your review may still be made public.

Do you want your identity to be public for this peer review? For information about this choice, including consent withdrawal, please see our Privacy Policy.

Reviewer #1: No

Reviewer #2: No

Answer

None.

Hopefully, we have made an appropriate revision based on these valuable comments and suggestions. Please let me know if there is anything else we need to do with the revision, and we’ll complete it as soon as possible.

We thank you wholeheartedly for your excellent work. Your kind assistance is greatly appreciated. We look forward to any future correspondence.

Yours sincerely,

Liangliang Ding

E-mail: 15887847798m0@sina.cn
